# Supplementary material for: Open-label, cluster randomised controlled trial and economic evaluation of a brief letter from a GP on unscheduled medical contacts associated with the start of the school year: the PLEASANT trial
Source: BMJ Open. 2018 Apr 20;8(4):e017367. doi: 10.1136/bmjopen-2017-017367 (PMC5914776; doi:10.1136/bmjopen-2017-017367)
Supplement: Supplementary file 1 [file bmjopen-2017-017367supp001.pdf]

1 APPENDICES

1.1 Appendix 1 . Trial Intervention

GP letterhead

< Address line 1>
< Address line 2>
< Address line 3>
< Address line 4>

<Insert Date>

Dear Parent

Please read this important letter regarding your child's asthma

It is really important that your child continues to take their asthma medication during the summer holidays. Returning to school is a time when asthma can get worse and make children and young people with asthma poorly. This may be due to contact with infections at the start of the new school year.

To reduce the chances of getting poorly when they return to school, your child should continue to take their asthma medication as prescribed by their GP or practice nurse. If your child has stopped taking their medication over the summer holidays it is important to start it again as soon as possible. If they are short of medication, or you are not sure of the proper dose, please get in touch with the practice.

Yours sincerely

<Name of Doctor>

1.2 Appendix 2. Changes to Protocol

**Changes to Protocol**		**REC approval date**	**Approved by**
Protocol Version 2 (14.05.15): This version included an additional secondary outcome to include data up to September 2014, to see if the effect from September 2013 is maintained when there is no study intervention thus extending the follow up period by one month (see section 2.1.5).	Agreed as a 2-month non-cost contact variation by HTA 02/02/2015	25th May 2014	NRES Committee Yorkshire & Humber – South Yorkshire

1.3 Appendix 3 - Practice Withdrawal and Adherence to Protocol

Table 4 provides the number of practices and the number of individuals aged 5-16 (the primary analysis population) included for each time period.

Table 4: Number of practices and individuals included within each time period

	**Letter**		**No letter**	
	**Practices**	**Individuals 5-16**	**Practices**	**Individuals 5-16**
Prescription uptake and scheduled medical contacts				
August 2013	68	5305	69	5586
August 2013-July 2014	58	4541	54	4549
August 2014	58	4541	54	4549
All medical contacts				
September 2013 (Primary study period)	68	5305	69	5586
September to December 2013 (extended study period)	65	5097	67	5384
September 2013-August 2014 (twelve month study period)	58	4541	54	4549
September 2014 (Echo sub-study)	57	4411	53	4438

1.4 Adherence to protocol

Of the 70 intervention practices, 2 did not send letters to any of the patients identified and 4 sent the intervention out late on the 6th, 8th, 12th and 23th of August. In addition, GPs were given discretion to withhold the letter from any children they believed were unsuitable candidates; among the remaining 64 practices (5222 individuals), letters were not sent to 786 children. These individuals were included in the primary ITT analyses but excluded from Per Protocol analyses.

1.5 Appendix 4 - Analysis of Respiratory Contacts

1.5.1 Analysis of unscheduled respiratory related contacts

The unscheduled medical respiratory related contacts are presented in Figure 3. The baseline data for the 12 months leading up to the intervention suggest that the practices randomised the intervention arm have more unscheduled respiratory contacts than the control arm. This feeds through into the summary statistics given in Table 5 which show more contacts in the intervention arm. When compared to Table 4 one can see that approximately 5% of medical contacts are respiratory-related in the analysis.

The adjusted analyses in Table 5 has the corresponding baseline term in the model. The analyses infer an increase in respiratory-related medical contacts. It should be noted though that the definition of a respiratory-related contact for the analysis included that a prescription for an asthma medication was given in the medical contact. The intervention has increased asthma prescriptions and so it is likely that a proportion of these contacts are associated with the increase in prescriptions.

To further explain the results, Table 6 breaks down of the type of respiratory-related contact by the time period. We can see here that in terms of total number of respiratory contacts the mean number of contacts per child is consistent between groups. It is likely that the intervention led to a greater proportion of respiratory contacts being coded as unscheduled. For September45% of all respiratory contacts in the intervention arm were coded as unscheduled compared with 35% in the control arm. This could be due to a prescription being collected in the contact – if the medical contact had no specific code to assign it as a scheduled contact, but had a prescription associated with it, then it would be coded as unscheduled.

Table 5. Analysis of unscheduled respiratory related medical contacts

	Time Period	Treatment Arm* Intervention (%)	Treatment Arm* Control (%)	Odds-Ratio+	95% Confidence Interval	Treatment Arm* Intervention (Mean)	Treatment Arm* Control (Mean)	Incidence Ratio+	95% Confidence Interval
All Children	Sep	5.3	4.2	1.30	1.03 to 1.66	0.06	0.05	1.30	1.02 to 1.66
	Sep-Dec	18.4	16.7	1.13	0.95 to 1.33	0.23	0.21	1.10	0.95 to 1.27
	Sep-Aug	38.0	35.3	1.05	0.87 to 1.33	0.57	0.53	1.04	0.90 to 1.20
Children Receiving Preventers	Sep	5.5	4.4	1.30	1.02 to 1.66	0.06	0.05	1.30	1.01 to 1.66
	Sep-Dec	19.2	17.4	1.13	0.96 to 1.34	0.24	0.22	1.10	0.95 to 1.28
	Sep-Aug	39.3	36.9	1.06	0.87 to 1.30	0.59	0.55	1.06	0.92 to 1.23

* the proportions and means are simple summary statistics

+ the odds-ratios and incidence ratios with the corresponding confidence intervals are from a formal statistical analysis allowing for covariates.

Table 6. Respiratory related medical contacts by type of contacts and time period

	Treatment Arm	Type of Contact: Total	Type of Contact: Scheduled	Type of Contact: Unscheduled
Sep	Intervention	0.13	0.07	0.06
	Control	0.13	0.09	0.05
Sep-Dec	Intervention	0.56	0.33	0.23
	Control	0.56	0.35	0.21
Sep-Aug	Intervention	1.43	0.86	0.57
	Control	1.44	0.91	0.53

Note that total contacts=scheduled+unscheduled. There is rounding error in the table for row 2 as 0.134=0.088+0.046
