# Supplementary material for: Open-label, cluster randomised controlled trial and economic evaluation of a brief letter from a GP on unscheduled medical contacts associated with the start of the school year: the PLEASANT trial
Source: BMJ Open. 2018 Apr 20;8(4):e017367. doi: 10.1136/bmjopen-2017-017367 (PMC5914776; doi:10.1136/bmjopen-2017-017367)
Supplement: Author's manuscript [file bmjopen-2017-017367.draft_revisions.pdf]

BMJ Open is committed to open peer review. As part of this commitment we make the peer review history of every article we publish publicly available.

When an article is published we post the peer reviewers' comments and the authors' responses online. We also post the versions of the paper that were used during peer review. These are the versions that the peer review comments apply to.

The versions of the paper that follow are the versions that were submitted during the peer review process. They are not the versions of record or the final published versions. They should not be cited or distributed as the published version of this manuscript.

BMJ Open is an open access journal and the full, final, typeset and author-corrected version of record of the manuscript is available on our site with no access controls, subscription charges or pay-per-view fees (http://bmjopen.bmj.com).

If you have any questions on BMJ Open's open peer review process please email info.bmjopen@bmj.com

BMJ Open

A Cluster Randomised Controlled Trial and Economic Evaluation of a Brief Letter from a GP on Asthma Episodes Associated with the Start of the School Year – The PLEASANT Trial

Journal:	*BMJ Open*
Manuscript ID	bmjopen-2017-017367
Article Type:	Research
Date Submitted by the Author:	25-Apr-2017
Complete List of Authors:	Julious, Steven; The University of Sheffield, Medical Statistics Group, ScHARR,<br>Horspool, Michelle; University of Sheffield, Medical Statistics Group, School of Health and Related Research (ScHARR),<br>Davis, Sarah; University of Sheffield, Health Economics and Decision Sciences, School of Health and Related Research (ScHARR)<br>Bortolami, Oscar; University of Sheffield, Medical Statistics Group, School of Health and Related Research (ScHARR)<br>Franklin, Matthew; University of Sheffield, Health Economics and Decision Sciences, School of Health and Related Research (ScHARR)<br>Smithson, Henry; University of Cork, Department of General Practice<br>Elphick, Heather; Sheffield Children's Hospital, Respiratory Department<br>Cooper, Cindy; University of Sheffield , Clinical Trials Research Unit, School of Health and Related Research (ScHARR)<br>Norman, Paul; University of Sheffield, Department of Psychology
Keywords:	Asthma < THORACIC MEDICINE, PRIMARY CARE, Cluster Trial, Randomised Controlled Trial, School-aged Children, Adherence

SCHOLARONE™
Manuscripts

A Cluster Randomised Controlled Trial and Economic Evaluation of a Brief Letter from a GP on Asthma Episodes Associated with the Start of the School Year – The PLEASANT Trial

Authors: Prof Steven A Julious[a], Dr Michelle J Horspool[a], Sarah Davis[b], Dr Oscar Bortolami[a], Dr Matthew Franklin[b], Prof W Henry Smithson[c], Prof Heather Elphick[d], Prof Cindy Cooper[e], Prof Paul Norman[f]

Author Institutions

[a] Medical Statistics Group, School of Health and Related Research (ScHARR), University of Sheffield, Sheffield, United Kingdom

[b] Health Economics and Decision Sciences, School of Health and Related Research (ScHARR), University of Sheffield, Sheffield, United Kingdom

[c] Department of General Practice, University of Cork, Cork, Ireland

[d] Respiratory Department, Sheffield Children's Hospital, Sheffield, United Kingdom

[e] Clinical Trials Research Unit, School of Health and Related Research (ScHARR), University of Sheffield, Sheffield, United Kingdom

[f] Department of Psychology, University of Sheffield, Sheffield, United Kingdom

Corresponding author:

Name: Prof Steven A Julious

Address: Medical Statistics Group, School of Health and Related Research, The University of Sheffield, Regent Court, Regent Street, Sheffield, S1 4DA

Tel: 0114 2220709

Fax: 0114 2220749

Email: s.a.julious@sheffield.ac.uk

For peer review only

ABSTRACT (298/300 words max)

Background: Asthma is known to be seasonal with peaks in school-aged children associated with the return to school following the summer vacation. A drop in prescription collection in August has been associated with an increase in the number of unscheduled contacts after the school return.

Objective: To assess whether a public health intervention delivered in general practice reduced unscheduled medical contacts.

Design: A cluster-randomised trial with a trial-based economic evaluation. The intervention group received a letter from their GP in late July outlining the importance of (re)taking asthma medication before the return to school. The control group was usual care

Setting: General practices in England and Wales

Participants: School ages children in 142 general practices.

Main Outcome: Proportion of children aged 5-16 who had an unscheduled contact in September. Secondary endpoints included collection of prescriptions in August and unscheduled contacts over 12 months. Economic endpoints were quality-adjusted life-years gained and health service costs.

Results: There was no evidence of effect (odds-ratio 1.09; 95% CI 0.96 to 1.25) on unscheduled contacts in September but for time intervals (Sep-Dec and Sep-Aug) the intervention reduced contacts: reducing total mean number of contacts per child over 12 months by 5%. The intervention increased the proportion of children collecting a prescription in August (odds-ratio 1.43; 95% CI 1.24 to 1.64) and scheduled contacts in August (odds-ratio 1.13; 95% CI 0.84 to 1.52).

The mean reduction in medical contacts informed the health economics. The economic analysis estimated that the intervention was cost saving - £36.07 per patient - with a high probability (96.3%) of being cost-saving.

For peer review only

Conclusions: The intervention succeeded in increasing children collecting prescriptions and having scheduled contacts in August. It did not reduce unscheduled care in September but after September there was evidence in favour of the intervention

Trial registration number: ISRCTN03000938

Key Words

Asthma, school-age children, primary care, cluster trial, general practice, randomised controlled trial, CPRD, unscheduled care, scheduled care, adherence

Funding details

This project was funded by the NIHR Health Technology Assessment programme

Strengths and limitations of this study

- The evaluation was a highly efficient study design using routine data to evaluate a general practice public health intervention designed with children with asthma and their parents
- The coding of the outcomes from the routine data did present challenges in the derivation of the outcomes with the assessment of adherence not possible.
- The intervention increased prescription update in the month prior to the return to school with 30% more prescriptions collected.
- There was no immediate effect in September but in the wide time intervals of September to December and September to August there was evidence of effect with a mean reduction in medical contacts
- The intervention was simple to implement and had good user acceptability and was cost saving: costing £1.34 per child to implement but saving £36.07 per child with a 96.3% probability of being cost saving

For peer review only

1 INTRODUCTION

Asthma episodes and deaths are known to be seasonal [1]. A number of reports have shown peaks in asthma episodes in school-aged children associated with the return to school following the summer vacation [2–10]. Children returning to school are exposed to a variety of novel respiratory insults including allergens and viruses, at a time of changing climactic conditions. It has previously been shown that viral infection and allergen exposure in allergen sensitised asthmatics are associated with increased hospital admissions for acute asthma [11].

Our previous research [12] confirmed the increase in unscheduled medical contacts with children with asthma being approximately twice as likely as controls to have an unscheduled medical contact with their doctor around the time of the return back to school. In the same study it was found that in August, immediately preceding the return back to school, there were 25% fewer prescriptions for inhaled corticosteroids, compared to July and September. Furthermore, patients who received a prescription for inhaled corticosteroids were less likely to have an unscheduled medical contact after the return to school.

Little is known about the factors that are associated with the drop in prescriptions in August. Research on adherence to paediatric asthma treatment in general has identified weak beliefs about the necessity of asthma medication as a key reason for non-adherence[13]. Given that asthma symptoms decline in the summer months this may lead to weaker beliefs about the necessity to take asthma medication. The GP letter was designed to address this belief by emphasising the importance of (re)taking asthma mediation prior to returning to school.

The current study is a cluster randomised trial to evaluate whether a letter sent from a GP at the start of the summer vacation reminding parents of children with asthma of the necessity of taking their asthma medication before the return back to school. The study evaluated whether the letter reduced unscheduled contacts after the return back to school and increased prescriptions in August

2 RESEARCH AIMS AND OBJECTIVES

The aim of the study was to assess if a general practice delivered public health intervention (a letter sent from the GP to parents/carers of school-aged children with asthma) can reduce the number of unscheduled medical contacts after the school return.

For peer review only

3 METHODS

3.1 Participants

Participants were school-aged children with asthma, aged between 4 and 16, registered with a general practitioner. The primary analysis population was the intention to treat population (ITT) among children aged between 5 years and 16 years of age.

The choice of the 5-16 age group as the primary analysis population is due to the difficulty associated with making a diagnosis of asthma among children below this age [14,15]. Patients aged 4-5 were analysed separately to those aged 5-16 and are not included in the paper. Additional analyses were restricted specifically to children who had received a prescription for steroid inhalers in the previous year.

3.2 Interventions

Sites were randomly allocated to either: Intervention Group - sending out the letter or Control Group - standard care (no letter)

The intervention was a letter sent from a GP to the parents/carers of children with asthma reminding them to maintain their children's medication and collect a prescription if they were running low (See Appendix 1). It also advised that should their child have stopped their medication it should be resumed as soon as possible

The letter template was developed based on standard letters already used in general practice and designed to address beliefs about the necessity of taking asthma medication before the return back to school. The wording of the letter had input from the study team, which includes a GP, Health Psychologist and Consultant Respiratory Paediatrician and was also discussed in detail at two patient and public events, that included school-aged children with asthma and their parents[16 17 18].

The intervention letters were sent out the week commencing 29th July 2013 to obviate the distraction of planning for family holidays and yet leave enough time for parents and children to renew prescriptions and gain benefit from the medication. The letter and the timing of the

For peer review only - http://bmjopen.bmj.com/site/about/guidelines.xhtml

letter was decided following discussion with the Patients and Public Involvement (PPI) group [17].

The details of the PPI consultation events will be discussed in Section 3.4.

3.3 Study Design

The study was a cluster randomised trial. [19] The effectiveness of the intervention was assessed on the basis of reduced unscheduled medical contacts after the return to school in September prescription uptake prior in August. The primary study period was 1st – 30th September 2013 after the return to school. The extended study period was 1st September - 31st December 2013, since asthma-related appointments are more frequent in these months for children with asthma. The full follow-up period was 12 calendar months from 1st September 2013 to 31st August 2014. Prescription uptake and scheduled medical contacts such as asthma reviews were evaluated during the periods August 2013 and August 2013-July 2014

The health economic analyses were based on a 12 month period from 1st August 2013 to 31st July 2014. The period starts a month earlier than the evaluation of medical contacts in order to incorporate the cost associated with delivering the intervention including any increase in prescriptions or medical contacts in response to the intervention that occurred during August 2013.

The primary outcome was the proportion of patients who had an unscheduled medical contact in September 2013.

The secondary outcomes evaluated included the number of unscheduled medical contacts in September 2013 and the number and proportion of any medical contacts (scheduled and unscheduled) in the same time interval as well as in the time intervals September-December 2013 and September 2013-August 2014. The analyses of the same outcomes were repeated for the other time intervals.

3.4 Patient Involvement

There were three PPI consulation events with children with asthma and their parents. The first consultation event was funded by a grant by NIHR Research Design Service for Yorkshire and the Humber prior to submission of the grant application in January 2011.

For peer review only - http://bmjopen.bmj.com/site/about/guidelines.xhtml

At this first consultation event it was agreed that a letter from their practice would be a useful reminder and not seen in any way as intrusive. A draft of the proposed letter was reviewed and the children fed back that they believed that the letter from their GP should be addressed to their parents rather than to themselves.

The second PPI consultation event was held after the grant was awarded in September 2012 [17]. At this meeting the intervention letter was finalised. The general feeling among the group was that the intervention did not adequately reflect the seriousness of asthma as a health condition. It was felt therefore that there was a danger that the intervention could be ignored by parents, or that the information it contained could be forgotten about. The letter was amended to reflect this input.

The consultation event also discussed the timing of the intervention and it was proposed to send the intervention the first week of August. The event also reviewed the lay summary for the study and provided input to the logo for the study.

Two parents also agreed to join the trial steering committee for the study. At the first trial steering committee meeting it was agreed to bring the timing of the intervention forward by a week to the end of July as asthma medication has a better chance of working the earlier they are used consistently.

A third PPI consultaiton event was held after the study had been completed which will be discussed in the discussion[20]. There is a web site where the PPI events are detailed (http://www.sheffield.ac.uk/scharr/sections/dts/ctru/pleasant/ppi Assessed 22 October 2016). There has also been a separate publication on the first two PPI consultation events [18]

3.5 Ethical approval and research governance

Ethical approval for the study was given by South Yorkshire Research Ethics Committee on 25th October 2012 (reference number 12/YH/04). NHS Permissions to conduct the study was obtained for all the Primary Care Trusts (PCTs) in England and Health Boards in Wales.

For peer review only - http://bmjopen.bmj.com/site/about/guidelines.xhtml

The trial was registered with the International Standard Randomised Controlled Trial Register (ISRCTN) reference number ISRCTN 03000938.

3.6 Setting

The setting was primary care with the unit of cluster being general practices. Site eligibility required practices to be using the Vision IT software and be part of Clinical Practice Research Datalink (CPRD). Site recruitment was conducted by CPRD and the NIHR Primary Care Research Network with the PLEASANT study team[21].

3.7 CPRD recruitment

A practice recruitment pack, consisting of a detailed study information sheet and an expression of interest (EoI) form, was sent to all 433 practices contributing to CPRD in England and Wales at the time of recruitment [22].

3.8 Randomisation and blinding

Randomisation was stratified by size of General Practice (i.e. the “list size”) to ensure that there was an equal sample size – in terms of number of school-age children with asthma – in each arm of the trial. The randomisation sequence was generated by a statistician based within the Sheffield CTRU, and allocation concealment was ensured by restricting access to the two CTRU statisticians. The study team were unblinded throughout the study but had no access to data until after a statistical analysis plan was developed and had no influence on data capture.

3.9 Data management

Data was collected through the CPRD which captures the coding for each consultation by staff in the practice. The medical consultations and diagnostic codes were reviewed to determine if each contact was a scheduled contact – such as a medicines review – or an unscheduled contact – such as an acute or an out of hours visit.

An independent GP adjudication panel was established to help in the coding. The adjudication panel met three times and did not have access to the randomisation group when reviewing the data. The adjudication panel reviewed and coded 4,600 unique terms into scheduled and unscheduled medical contacts. These terms accounted for 92% of all medical

For peer review only

contacts but 17% of all terms used in the study. Terms not coded by the adjudication panel were coded as unscheduled. In addition, 7.9% of all contacts did not have any terms to indicate consultation type or diagnosis and free text was used in the database system to which the study team had no access. The adjudication panel advised to code these contacts as unscheduled.

3.10 Statistical methods

3.10.1 Analysis populations

The study was designed to detect a difference of 5% (30 v 25%) with 90% power and two sided significance level of 5%, with an intra-class correlation (ICC) of 0.03 to account for clustering. Based on this we estimated that we required 70 practices per arm. It was anticipated that the sample size of 140 practices would equate to approximately 14,000 school-age children with asthma.

Each of the outcomes were evaluated on each of subpopulations: children aged 5-16 (the primary analysis population) and children aged 5-16 and who have prescriptions for steroid preventer. The analyses were restricted specifically to children who had received a prescription for preventer inhalers in the previous year as this was intended to identify the treatment effect in the population likely to receive most benefit.

The primary analyses of effectiveness were performed on both ITT with analyses also conducted on the per protocol (PP). The health economic analyses were based on the PP population. ITT analyses comprised all practices for whom data were obtained by study period. The two criteria for exclusion from PP analyses were: practices that did not send intervention as requested. In such cases, the entire practice data was excluded from PP analyses and Individual children who were not sent the intervention letter. GPs were given discretion to withhold the letter from any children they believed were unsuitable. In such cases, the individual was excluded from PP analyses.

For peer review only

3.10.2 Analytical methods

The proportion of children having an unscheduled medical contact was analysed separately for each time period using logistic regression with the individual's age, gender, number of contacts the previous September as covariates, the trial arm (intervention or control) as a fixed effect, and the design/cluster effect of general practice as a random effect. The proportion of children having a prescription within each time period was analysed in the same manner. The number of unscheduled medical contacts made in each period by the children as well as the number of prescriptions ordered within a time period, were both analysed using a random effects negative binomial model in which the same covariates as above were included. [23]

3.11 Health economic methods

An economic evaluation was undertaken to compare the incremental cost per quality adjusted life year (QALY) of the reminder letter versus standard care. The perspective of the analysis was that of the NHS (primary and secondary care resource-use based on available CPRD data and associated costs). We assumed the intervention would have no impact on quality of life (utility) beyond 4 months or mortality so QALYs were calculated for the four month post-intervention time period. Costs were calculated for one year post-intervention. The overall time horizon was one year from the intervention and therefore no discounting was applied. QALYs were estimated using the area under the curve (AUC) method[24] by assigning utility values to exacerbation-related contacts – a systematic review identified these utility values[25]. Bootstrapped costs were evaluated 12 months post-intervention with one year linear regression-based baseline adjustment (BA). Previous authors have recommended the use of BA analysis for economic evaluations to control for baseline differences between trial arms which may not be controlled for by the randomisation process,[26 27 28] only baseline costs were controlled for because other baseline aspects were not sufficiently present in CPRD (such as baseline utility).

Resource-use included unscheduled and scheduled contacts in the year following intervention were included in the economic analysis to capture any change in healthcare resource-use in response to the letter. Prescriptions in the year following the intervention for asthma medications used in the management of chronic asthma and asthma medications used in the treatment of acute exacerbations were included to establish if the cost of prescriptions had

For peer review only

increased in response to the letter intervention. It was considered necessary to include costs in the year following intervention to distinguish between an increase in the number of scheduled contacts and a change in the timing of the scheduled contacts.

The cost of the letter intervention was included for intervention practices with no cost included for practices in the control arm as standard care was assumed to be the same in both intervention and control practices.

Full details of the methods used in the economic analysis will be published in a separate paper.

3.12 Trial oversight

A Trial Steering Committee (TSC) was established to give oversight to the study. The TSC consisted of an independent chair (GP), two independent members (academic GP and statistician) and two lay members (parents of children with asthma) along with the Principal Investigator and key staff within the CTRU (as non-voting members). The role of the TSC was to provide supervision of the protocol, statistical analysis plan and to provide advice on and monitor progress of the trial.

4 TRIAL RESULTS

4.1 Recruitment and participant flow

In total, 142 practices agreed to take part in the study [22]. Of these practices, one (a control group practice with 99 children with asthma) withdrew consent after the start of the study for the data to be extracted and stored by the CPRD (independent of the study); this practice was excluded from all analyses. In total, 70 practices (comprising 5917 individuals) were randomised to the intervention (letter), and 71 practices (6262 individuals) to control. The CONSORT diagram is given in Figure 1

4.2 Baseline characteristics

The descriptive statistics of the 12179 subjects and 141 practices are included in Table 1a and Table 1b. Summaries reported stratified by intervention type and overall.

For peer review only

4.3 Number of participants and analysis subsets

For each study period, analyses were based only on practices that contributed data to the entirety of that period. In other words, if practices stopped submitting data to CPRD before the end of a given follow-up period they were excluded from all analyses for that time period. GP practices no longer being able to contribute to the CPRD during the 12 month follow up period due to their leaving the Vision IT system. Details of the practices within the study during each time period are given in Appendix 3.

Figure 1 shows the flow of subjects for the primary analysis population (aged 5-16). Of the 456 practices invited, 433 were through the CPRD and 23 were through the primary care research network and joined the CPRD [22]. There were zero GP exclusions in the arm that did not send letters, as it is impossible for the GPs to exclude individuals from receiving letters when no one in that arm is receiving letters.

4.4 Clinical results

In the primary analysis the proportion of individuals who had at least one unscheduled medical contact in September was 45.2% in the intervention arm, compared with 43.7% in the control arm (adjusted odds ratio (OR) = 1.09, 95% CI 0.96 to 1.25 (see Table 2). In terms of means contacts the number of unscheduled contacts are comparable (incidence rate ratio IRR=1.02 95% CI 0.94 to 1.12). The results are comparable for children receiving preventer medication.

The results for the incidence rate ratios across the time periods are given in Figure 2.

After September there was evidence of a reduction in the mean number medical contacts. The incidence ratio declines as longer time periods are analysed (see Table 2) suggesting that the short-term increase in unscheduled contacts in September is gradually outweighed by decreases in unscheduled and scheduled contacts in the longer-term. The total number of medical contacts in the twelve months is reduced by 5% (IRR=0.95 with 95% CI 0.91 to 0.99). In the control group the total number of contacts is 12.08 per child and so a 5% reduction from 12.08 would equate to a mean reduction of 0.60 contacts per child.

For peer review only

The intervention (letter) was associated with an increased uptake of prescriptions in the month of August 2013 – see Table 3. In August, 876 (16.5%) had at least one prescription compared with 703 (12.6%) in the control group (adjusted odds ratio 1.43, 95% CI 1.24 to 1.64); the total number of prescriptions was also higher (adjusted incidence rate ratio 1.31, 95% CI 1.17 to 1.48). Scheduled contacts made in August 2013 also increased (adjusted odds ratio 1.13, 95% CI 0.84 to 1.52).

There is evidence of user acceptability of the intervention with over half the practices in the intervention (13 out of 24) who responded to a survey saying they repeated the intervention the year following the study [29]

4.5 Health economic results

The full results of the economic evaluation will be published in a separate paper so only key BA base-case results are provided here. The average cost per child of sending the intervention was £1.34 per child. The fall in medical contacts over one year described in the clinical results led through into the health economic assessment. A mean reduction in costs per child of £36.07 was estimated and there was 96.3% certainty of the intervention being cost saving. The economic evaluation estimated a mean QALY loss of 0.00017 which is practically zero.

5 DISCUSSION

PLEASANT was a highly efficient innovative study design as it used routine data for all outcomes and the delivery of the intervention was centrally automated through the CPRD which makes for a highly efficient study design. By our own estimation a substantial six figure sum is saved compared to a trial where GP practices would need to be visited to collect the data.

Previous work has shown an increased in the number of unscheduled medical contacts by children in autumn months (September to December), which may be due to the start of the new school term [30]. By sending a letter at the start of the school holidays to remind children

For peer review only

of the importance of taking their medication, it was hypothesised that the increase may be averted. More specifically, it was predicted that a reminder letter would lead to a greater uptake of inhaler prescriptions in August that, in turn, would lead to increased adherence and, finally, fewer unscheduled medical appointments.

There was evidence of an impact on the first part of this pathway as the intervention group demonstrated a higher uptake of prescriptions in August 2013. They also had an increase in scheduled contacts in the same month. The data is not available to confirm actual medicine usage and so it is unclear whether the increased uptake also translated into an increased usage.

The original plan was to assess this through the medicines procession ratio – which estimates the time a child has collected medication for over the time the child should have collected time for. This could not be estimated for these data however due to the inadequate recording in the routine data of the prescription data. Our analysis of the PLEASANT dataset suggests that further work is required to determine how to assess adherence using such data.

The primary endpoint was unscheduled medical contacts in September 2013, which coincided with the start of the new school term. There was no evidence of a reduction in the intervention group. In fact there was an increase in the proportion of children who had an unscheduled medical contact in September.

The increase could be caused by GPs needing to see certain patients before giving a new prescription if they had not had a prescription recently. Evidence to support this is a post hoc observation that for children who had collected a prescription within the last 3 months prior to the start of the study there was less evidence of an increase in unscheduled contacts in September - 55.2% in the intervention arm compared to 54.3% on control. This compared patients whose last prescription was 3-6 months prior to the start of the study where the excess was greater - 42.1% in the intervention arm against 39.7% on control.

The way the unscheduled contacts were coded could have also impacted on the outcome. The intervention increased prescription update and collection of a prescription for asthma medication was used to definition of an unscheduled medical contact.

Despite lack of reduction in the proportion of children having unscheduled contacts in September, both the total number of contacts per child (i.e. scheduled plus unscheduled) and unscheduled contacts were lower in the intervention group than in the control over the extended study period (September to December 2013) and the full year (September 2013 to August 2014). Although the effects were not statistically significantly, the minimal cost associated with the intervention meant the intervention was found to have a high probability of being cost-saving overall.

With such a relatively low cost intervention, £1.34 per child, and an average cost for an unscheduled surgery visit circa £50, an intervention would only need to reduce the number of contacts by 3 per year for an average practice with 85 asthmatic children to be cost neutral. The evidence from the trial is that contacts are reduced 0.6 per child in the 12 months after intervention, or 51 per year for an average practice of 85 children.

The economic analysis (which used data over a 12 month period from August 2013 to July 2014) estimated a mean cost saving across the base case of £36.07 per child. So, although the study did not have a significant effect for the primary endpoint, the minimal cost associated with the intervention meant the intervention was found to have a high probability (96.3%) of being cost-saving overall.

In the UK alone there are over one million children with asthma. The intervention thus has potential to provide health service savings if implemented.

The results were discussed with children with asthma and their parents at a PPI consultation event[20]. At the event it was fed back the savings per child was an important result and the advice was if the impact of the intervention would have been greater if it had been repeated over a number of years. The letter could then assist parents and children as they plan for the school return each year.

There is evidence of good user acceptability with over half the practices who responded to a survey repeating the intervention the year after the study. Once the intervention is set up for one year the costs then associated with sending it out are less due to many of the school age children with asthma being the same from year to year

For peer review only

6 CONCLUSIONS

The intervention succeeded in increasing the number of children collecting a prescription in August, along with the proportion of children who had scheduled contacts in the same month.

The intervention did not reduce unscheduled care as expected in September, which was the primary endpoint, however, over a longer time period there is evidence that the intervention reduced medical contacts. This is reflected in the health economic evaluation which overall showed that the intervention had a high probability of giving a cost saving.

With the strong evidence from the trial of an increase in August of both prescription collection and evidence of cost reduction practices may wish to implement the intervention. Particularly practices with high rates of unscheduled medical care.

7 REFERENCES

1. Campbell, M. J., Holgate, S. T. & Johnston, S. L. Trends in asthma mortality. *BMJ* **315,** 1012 (1997).
2. Storr, J. & Lenney, W. School holidays and admissions with asthma. *Arch. Dis. Child.* **64,** 103–7 (1989).
3. Grech, V., Balzan, M. & Distefano, S. Paediatric Wheezy Admissions at and around School Holiday Periods. *Malta Med. J.* **16,** 23–26 (2004).
4. Kimes, D. *et al.* Temporal dynamics of emergency department and hospital admissions of pediatric asthmatics. *Environ. Res.* **94,** 7–17 (2004).
5. Kimbell-Dunn, M., Pearce, N. & Beasley, R. Seasonal variation in asthma hospitalizations and death rates in New Zealand. *Respirology* **5,** 241–6 (2000).
6. Gergen, P. J., Mitchell, H. & Lynn, H. Understanding the seasonal pattern of childhood asthma: results from the National Cooperative Inner-City Asthma Study (NCICAS). *J. Pediatr.* **141,** 631–6 (2002).
7. Harju, T., Keistinen, T., Tuuponen, T. & Kivelä, S. L. Seasonal variation in childhood asthma hospitalisations in Finland, 1972-1992. *Eur. J. Pediatr.* **156,** 436–9 (1997).
8. Julious, S. A., Osman, L. M. & Jiwa, M. Increases in asthma hospital admissions associated with the end of the summer vacation for school-age children with asthma in two cities from England and Scotland. *Public Health* **121,** 482–4 (2007).

For peer review only

9. Silverman, R. A., Stevenson, L. & Hastings, H. M. Age-related seasonal patterns of emergency department visits for acute asthma in an urban environment. *Ann. Emerg. Med.* **42,** 577–586 (2003).

10. Fleming, D. M., Cross, K. W., Sunderland, R. & Ross, A. M. Comparison of the seasonal patterns of asthma identified in general practitioner episodes, hospital admissions, and deaths. *Thorax* **55,** 662–5 (2000).

11. Murray, C. S. *et al.* Study of modifiable risk factors for asthma exacerbations: virus infection and allergen exposure increase the risk of asthma hospital admissions in children. *Thorax* **61,** 376–82 (2006).

12. Julious, S. A., Campbell, M. J., Bianchi, S. M. & Murray-Thomas, T. Seasonality of medical contacts in school-aged children with asthma: association with school holidays. *Public Health* **125,** 769–76 (2011).

13. Drotar, D; Bonner, M. Influences on Adherence to Pediatric Asthma Treatment: A Review of Correlates and Predictors. *J. Dev. Behav. Pediatr* **30,** 574–82 (2009).

14. British Thoracic Society / Scottish Intercollegiate Guideline. *British Guideline on the Management of Asthma: A national clinical guideline.* (2012). at <https://www.brit-thoracic.org.uk/document-library/clinical-information/asthma/btssign-guideline-on-the-management-of-asthma/>

15. Bush, A. Diagnosis of asthma in children under five. *Prim. Care Respir. J.* **16,** 7–15 (2007).

16. Julious SA, Lai J, Boote J, Elphick H, S. H. Report of a patient and public involvement consultation event with children with asthma and their parents to review a proposal for a primary care intervention around the September school return. *Universtity Sheff. ScHARR Rep. Ser. ISBN 1 900752 18 2* **25,** ISBN 1 900752 18 2 (2011).

17. Julious, SA, Horspool M J, Boote, J. PLEASANT TRIAL: 2nd Consultation event with children with asthma and their parents. *Univ. Sheffield. ScHARR Rep. Ser. No 28. ISBSN 1 900752 14 X* (2012). at <https://www.shef.ac.uk/scharr/sections/dts/ctru/pleasant/ppi/sept2012>

18. Boote, J. *et al.* PPI in the PLEASANT trial: involving children with asthma and their parents in designing an intervention for a randomised controlled trial based within primary care. *Prim. Health Care Res. Dev.* 1–13 (2016).

For peer review only

doi:10.1017/S1463423616000025

19. Horspool, M. J. *et al.* Preventing and lessening exacerbations of asthma in school-age children associated with a new term (PLEASANT): study protocol for a cluster randomised control trial. *Trials* **14,** 297 (2013).

20. Simpson, RM, Julious, SA, Mooney, C, Norman, P. PLEASANT TRIAL: 3rd Consultation event with children with asthma and their parents. *Univ. Sheff. ScHARR Rep. Ser. No 32. ISBN 1900752344.* (2015). at <http://www.sheffield.ac.uk/polopoly_fs/1.541036!/file/PLEASANT_PPIReport_v2.pdf>

21. Horspool, M. *et al.* Preventing and Lessening Exacerbations of Asthma in School-aged children Associated with a New Term (PLEASANT): Recruiting Primary Care Research Sites-the PLEASANT experience. *NPJ Prim. care Respir. Med.* **25,** 15066 (2015).

22. Horspool, M. J. *et al.* Preventing and Lessening Exacerbations of Asthma in School-aged children Associated with a New Term (PLEASANT): Recruiting Primary Care Research Sites-the PLEASANT experience. *NPJ Prim. care Respir. Med.* **25,** 15066 (2015).

23. Julious, S. A., Osman, L. M. & Jiwa, M. Increases in asthma hospital admissions associated with the end of the summer vacation for school-age children with asthma in two cities from England and Scotland. *Public Health* **121,** 482–4 (2007).

24. Drummond, MF; Sculpher, MJ; Torrance, GW; O'Brien, BJ; Stoddart, G. *Methods for the economic evaluation of health care programme*. (Oxford University Press, 2005).

25. Kua, W. S. & Davis, S. *School of Health And Related Research Health Economics & Decision Science (HEDS) Discussion Paper Series Systematic review of health st ate utilities in children with asthma*. at <https://www.shef.ac.uk/scharr/sections/heds/discussion-papers/16-12-1.588796>

26. Hunter, Rachael Maree; Baio, Gianluca; Butt, T. An Educational Review of the Statistical Issues in Analysing Utility Data for Cost-Utility Analysis. *Pharmacoeconomics* **33,** 355–66 (2015).

27. Dyfig, R. C. H. Methods for the collection of resource use data within clinical trials: a systematic review of studies funded by the UK Health Technology Assessment

For peer review only

program. *Value Heal.* **13,** 867–82 (2010).

28. van Asselt, Antoinette; van Mastrigt, Ghislaine; Dirksen, Carmen; Arntz, Arnoud; Severens, Johan; Kessels, A. How to Deal with Cost Differences at Baseline. *Pharmacoeconomics* **27,** 519–28 (2009).

29. Hatfield I, Julious SA, Davis S, Horspool MJ, Norman P, M. C. An assessment of the resources used by General Practices in the intervention arm of the PLEASANT study in sending out the intervention. *Univ. Sheffield. ScHARR Rep. Ser. No 30 ISBN 1 900752 24 7* (2015). at <https://www.shef.ac.uk/polopoly_fs/1.529487!/file/AnalysisofthePLEASANTQuestionnaire.pdf>

30. Julious, S. A., Campbell, M. J., Bianchi, S. M. & Murray-Thomas, T. Seasonality of medical contacts in school-aged children with asthma: association with school holidays. *Public Health* **125,** 769–76 (2011).

31. Yong, Y. V. & Shafie, a. a. Economic evaluation of enhanced asthma management: A systematic review. *Value Heal.* **16,** A377 (2013).

For peer review only

Study Registraton

ISRCTN03000938 - assigned 19/10/2012

Contributors

Steven A. Julious, Professor Medical Statistics; Michelle J Horspool, Trial Manager; Mike Bradburn, Senior Statistician; Amanda Loban, Data Manager; Sarah Davis, Health Economist; Matthew Franklin, Health Economist, together produced the first draft of the report

The following conceived of or designed the work: Steven A. Julious, Professor of Medical Statistics; Michelle J Horspool, Trial Manager; Sarah Davis, Health Economist; W Henry Smithson, Prof. Primary Care; Healther Elphick, Consultant Respiratory Paediatrician; Paul Norman, Prof Health Psychology; Cindy L Cooper, Sheffield CTRU Director.

The following were involved in the interpretation of data for the work: Amanda Loban, Data Manager; Steven A Julious, Prof Medical Statistics; Michelle Horspool, Trial Manager;; W Henry Smithson; Prof. Primary Care.

The following were involved in the analysis of data: Oscar Bortolami, Medical Statistician; Sarah Davis, Health Economist; Matthew Franklin, Health Economist.

The following drafted the paper: Steven A. Julious, Professor Medical Statistics; Michelle J Horspool, Trial Manager; Sarah Davis, Health Economist; Matthew Franklin, Health Economist;

The following reviewed the work critically for important intellectual content. Steven A. Julious, Professor Medical Statistics; Michelle J Horspool, Trial Manage, Oscar Bortolami, Medical Statistician; Sarah Davis, Health Economist; Matthew Franklin, Health EconomistW Henry Smithson, Prof Primary Care; Heather Elphick, Consultant Respiratory Paediatrician; Paul Norman, Prof of Health Psychology; Cindy L Cooper, CTRU Director.

All authors agree to be accountable for all aspects of the work in ensuring that questions related to the accuracy or integrity of any part of the work are appropriately investigated and resolved.

Declaration of interests

We have read and understood BMJ policy on declaration of interests and declare that we have no competing interests

For peer review only

All authors have completed the ICMJE uniform disclosure form at www.icmje.org/coi_disclosure.pdf and declare: no support from any organisation for the submitted work; no financial relationships with any organisations that might have an interest in the submitted work in the previous three years; no other relationships or activities that could appear to have influenced the submitted work.

Funding for this study was provided by the Health Technology Assessment programme of the National Institute for Health Research Programme (Grant 11/01/10)

Exclusive License

The Corresponding Author has the right to grant on behalf of all authors and does grant on behalf of all authors, a worldwide licence to the Publishers and its licensees in perpetuity, in all forms, formats and media (whether known now or created in the future), to i) publish, reproduce, distribute, display and store the Contribution, ii) translate the Contribution into other languages, create adaptations, reprints, include within collections and create summaries, extracts and/or, abstracts of the Contribution, iii) create any other derivative work(s) based on the Contribution, iv) to exploit all subsidiary rights in the Contribution, v) the inclusion of electronic links from the Contribution to third party material where-ever it may be located; and, vi) licence any third party to do any or all of the above

Transparency declaration

The lead author, and manuscript guarantor, affirms that this manuscript is an honest, accurate, and transparent account of the study being reported; that no important aspects of the study have been omitted; and that any discrepancies from the study as planned (and, if relevant, registered) have been explained.

Data Sharing

Access to patient level data is provided by the CPRD for health research purposes and is dependent on approval of a study protocol by the MHRA Independent Scientific Advisory Committee (ISAC). More information on ISAC and the protocol submission process can be found at: www.cprd.com/isac (date accessed 18 April 2017)

Sponsor

For peer review only

The study was sponsored by Sheffield Clinical Commissioning Group. The authors has freedom to act in the writing and submission of the paper.

Acknowledgments

We gratefully acknowledge the hard work, support and advice from the following: Hilary Pinnock, (Professor, University of Edinburgh for her advice; Gerry McCann, Zaynah Gurreebun, Rachael Williams, Robin May and Jennifer Campbell, Clinical Practice Research Datalink (Clinical Practice Research Datalink, CPRD) for their contribution to site recruitment and to Tjeer Van Staa (CPRD) for his advice on CPRD; Cara Mooney, David White (University of Sheffield) for support with study set up, site recruitment and site set up and close down;Dan Beever and Helen Wakefield for administrative and clerical support; Wei Sun Kua, for her Health Economic contribution; Jonathan Boote, as PPI Lead; Mike Bradburn and Neil Shephard for the statistical input and Amanda Loban for data management

We are very grateful to the children and parents who took part in the three patient and public events through all stages of the study for their advice and input.

We would like to especially thank the GP adjudication panel Dr Mark Boon (Conisbrough Group Practice), Dr Karen Forshaw (Bentley Surgery), Dr Julie Hackney (The Avenue Surgery) for their advice, steer and valued contributions.

We offer special thanks to the members of our oversight committee: Trial Steering Committee: Dr Steve Holmes (Independent Chair, General Practitioner), Prof Andrew Wilson (Independent Academic General Practitioner, University of Leicester), Dr Martyn Lewis (Independent Statistician, Keele University), Zaida Bibi (Independent Parent Representative), and Camilla Mills (Independent Parent Representative).

The views expressed in this report are those of the authors and not necessarily those of the National Institute for Health Research Health Technology Assessment programme. Any errors are the responsibility of the authors.

For peer review only

Trial Summary

What is already known about this topic? We have not identified any other studies which have examined the economic benefits of a simple postal intervention in asthma patients and therefore it is difficult to compare our results to those of existing published studies. Yong and Shafie [31] have published a systematic review which looked more broadly at non-pharmacological interventions that aimed to enhance asthma management. The interventions included by Yong and Shafie varied from educational and self-management interventions to environmental interventions. Whilst the PLEASANT intervention letter could be considered to be a simple form of patient education, the educational interventions included by Yong and Shafie were all more intensive than the postal intervention used in PLEASANT, and the population was not restricted to school-aged children, making comparisons difficult. However, the broader evidence reviewed by Yong and Shafie suggests that non-pharmacological interventions which aim to improve an individual's management of their asthma have the potential to be cost-effective.

What are the new findings? The intervention in PLEASANT caused an increase in prescription collection in August as well as scheduled medical contacts. It did not then reduce medical contacts in September but after September there was evidence of a fall in medical contacts which followed through in the economic analysis to give a high probability of the intervention being cost-saving.

How might this influence practice? The increase in prescriptions and scheduled contacts in August could lead to individual GP practices wishing to implement the intervention. Evidence from the trial suggests this would decrease overall costs associated with the asthma management and reduce unscheduled care.

Table 1: Descriptive statistics of patients and surgeries

a) Descriptive statistics of gender (frequencies and percentages reported) and age (mean, SD, median, interquartile range and range reported). Statistics produced at subject level.

Variable	Letter (N=5917)	No Letter (N=6262)	Total (N=12179)
Gender	Male (%)<br>3505 (59.24)<br><br>Female (%)<br>2412 (40.76)	Male (%)<br>3749 (59.87)<br><br>Female (%)<br>2513 (40.13)	Male (%)<br>7254 (59.56)<br><br>Female (%)<br>4925 (40.44)
Age	Mean (SD)<br>10.51 (3.29)<br>Median (IQR)<br>10.80 (7.88 - 15.97)<br>Range<br>4.05 - 15.97	Mean (SD)<br>10.55 (3.30)<br>Median (IQR)<br>10.89 (7.80 - 15.97)<br>Range<br>4.05 - 15.97	Mean (SD)<br>10.53 (3.30)<br>Median (IQR)<br>10.89 (7.80 - 15.97)<br>Range<br>4.05 - 15.97

b) Descriptive statistics of size (mean, SD, median, interquartile range and range reported). Statistics produced at surgery level.

Variable	Letter (N=70)	No Letter (N=71)	Total (N=141)
Size	Mean (SD)<br>85 (44)<br>Median (IQR)<br>80 (49 - 114)<br>Range<br>4-209	Mean (SD)<br>88 (64)<br>Median (IQR)<br>75 (41 - 107)<br>Range<br>10-293	Mean (SD)<br>86 (55)<br>Median (IQR)<br>76 (45-113)<br>Range<br>4-293

For peer review only

Table 2. Analysis of unscheduled and total medical contacts

A. For all children in the intent to treat population.

	Time Period	Treatment Arm* Intervention (%)	Treatment Arm* Control (%)	Odds-Ratio+	95% Confidence Interval	Treatment Arm* Intervention (Mean)	Treatment Arm* Control (Mean)	Incidence Ratio+	95% Confidence Interval
Unscheduled Contacts	Sep	45.2	43.7	1.09	0.96 to 1.25	0.81	0.81	1.02	0.94 to 1.12
	Sep-Dec	80.1	79.1	1.10	0.96 to 1.26	3.19	3.32	0.98	0.93 to 1.04
	Sep-Aug	93.1	93.3	0.97	0.82 to 1.15	9.08	9.37	0.97	0.95 to 1.04
Total Contacts	Sep	57.8	58.4	0.99	0.80 to 1.22	1.05	1.10	0.97	0.87 to 1.07
	Sep-Dec	89.3	88.4	1.06	0.89 to 1.27	4.31	4.43	0.95	0.90 to 1.02
	Sep-Aug	96.6	96.4	0.89	0.71 to 1.12	11.52	12.08	0.95	0.91 to 0.99

* the proportions and means are simple summary statistics

+ the odds-ratios and incidence ratios with the corresponding confidence intervals are from a formal statistical analysis allowing for covariates.

B. For children receiving preventer medication in the intent to treat population.

	Time Period	Treatment Arm* Intervention (%)	Treatment Arm* Control (%)	Odds-Ratio+	95% Confidence Interval	Treatment Arm* Intervention (Mean)	Treatment Arm* Control (Mean)	Incidence Ratio+	95% Confidence Interval
Unscheduled Contacts	Sep	46.3	45.4	1.07	0.94 to 1.23	0.83	0.84	1.01	0.92 to 1.10
	Sep-Dec	81.3	81.4	1.04	0.90 to 1.21	3.27	3.44	0.97	0.92 to 1.03
	Sep-Aug	93.9	94.6	0.84	0.69 to 1.02	9.31	9.71	0.98	0.92 to 1.14
Total Contacts	Sep	59.1	60.4	0.97	0.79to 1.21	1.08	1.14	0.96	0.86 to 1.07
	Sep-Dec	90.4	90.5	0.98	0.81 to 1.19	4.43	4.70	0.95	0.89to 1.01
	Sep-Aug	97.1	97.3	0.81	0.64 to 1.01	11.82	12.53	0.96	0.90 to 1.12

* the proportions and means are simple summary statistics

+ the odds-ratios and incidence ratios with the corresponding confidence intervals are from a formal statistical analysis allowing for covariates.

For peer review only

Table 3. Analysis of prescription and scheduled contacts for August.

		Treatment Arm*				Treatment Arm*			
		Intervention (%)	Control (%)	Odds-Ratio+	95% Confidence Interval	Intervention (Mean)	Control (Mean)	Incidence Ratio+	95% Confidence Interval
Prescriptions	All Children	16.5	12.6	1.43	1.24 to 1.64	0.17	0.13	1.31	1.17 to 1.48
	Preventer	17.3	13.4	1.41	1 23to 1.63	0.18	0.14	1.30	1.16 to 1.47
Scheduled Contacts	All Children	14.3	13.9	1.13	0.84 to 1.52	0.17	0.16	1.17	1.06 to 1.29
	Preventer	14.8	14.4	1.14	0.84 to 1.54	0.18	0.17	1.17	1.06 to 1.29

* the proportions and means are simple summary statistics

+ the odds-ratios and incidence ratios with the corresponding confidence intervals are from a formal statistical analysis allowing for covariates.

For peer review only

8 APPENDICES

8.1 Appendix 1 . Trial Intervention

GP letterhead

< Address line 1>

< Address line 2>

< Address line 3>

< Address line 4>

<Insert Date>

Dear Parent

Please read this important letter regarding your child's asthma

It is really important that your child continues to take their asthma medication during the summer holidays. Returning to school is a time when asthma can get worse and make children and young people with asthma poorly. This may be due to contact with infections at the start of the new school year.

To reduce the chances of getting poorly when they return to school, your child should continue to take their asthma medication as prescribed by their GP or practice nurse. If your child has stopped taking their medication over the summer holidays it is important to start it again as soon as possible. If they are short of medication, or you are not sure of the proper dose, please get in touch with the practice.

Yours sincerely

<Name of Doctor>

For peer review only

For peer review only - http://bmjopen.bmj.com/site/about/guidelines.xhtml

8.2 Appendix 2. Changes to Protocol

Changes to Protocol		REC approval date	Approved by
Protocol Version 2 (14.05.15): This version included an additional secondary outcome to include data up to September 2014, to see if the effect from September 2013 is maintained when there is no study intervention thus extending the follow up period by one month (see section 2.1.5).	Agreed as a 2-month non-cost contact variation by HTA 02/02/2015	25th May 2014	NRES Committee Yorkshire & Humber – South Yorkshire

For peer review only

For peer review only - http://bmjopen.bmj.com/site/about/guidelines.xhtml

8.3 Appendix 3 - Practice Withdrawal and Adherence to Protocol

Table 4 provides the number of practices and the number of individuals aged 5-16 (the primary analysis population) included for each time period.

Table 4: Number of practices and individuals included within each time period

	**Letter**		**No letter**	
	**Practices**	**Individuals 5-16**	**Practices**	**Individuals 5-16**
Prescription uptake and scheduled medical contacts				
August 2013	68	5305	69	5586
August 2013-July 2014	58	4541	54	4549
August 2014	58	4541	54	4549
All medical contacts				
September 2013 (Primary study period)	68	5305	69	5586
September to December 2013 (extended study period)	65	5097	67	5384
September 2013-August 2014 (twelve month study period)	58	4541	54	4549
September 2014 (Echo sub-study)	57	4411	53	4438

8.4 Adherence to protocol

Of the 70 intervention practices, 2 did not send letters to any of the patients identified and 4 sent the intervention out late on the 6th, 8th, 12th and 23th of August. In addition, GPs were given discretion to withhold the letter from any children they believed were unsuitable candidates; among the remaining 64 practices (5222 individuals), letters were not sent to 786 children. These individuals were included in the primary ITT analyses but excluded from Per Protocol analyses.

For peer review only - http://bmjopen.bmj.com/site/about/guidelines.xhtml

Figure 1. CONSORT Diagram

Enrollment

GP practices approached (n=456 practices)

Randomized (n=141 practices; n=12179 individuals)

Experimental

Allocated to intervention (n=70 practices; n=5917 individuals)
- Received allocated intervention (n=4436 individuals)
- Did not receive allocated intervention as per protocol (n=1481 individuals)
  - GP exclusions (n=786 individuals)
  - Letter sent late or not at all (n=6 practices; n=695 individuals)

Control

Allocated to intervention (n=71 practices; 6262 individuals)
- Received allocated intervention (n=6262)
- Did not receive allocated intervention (n=0)

Allocation

Follow-Up

Experimental – Discontinued intervention:
- Withdrawal <2014-09-30 (n=13 practices; n=1506 individuals)

Control – Discontinued intervention:
- Withdrawal <2014-09-30 (n=18 practices; n=1824 individuals)

Analysis

Experimental – Analysed:

		ITT	PP
Prescription uptake & scheduled medical contacts	**Aug 13**	N_P = 68<br>P_I = 5305<br>P_O = 612*ƚ	N_P = 62<br>P_I = 4203<br>P_O = 1714*ƚ
	**Aug 13-Jul 14**	N_P = 58<br>P_I = 4541<br>P_O = 764	N_P = 54<br>P_I = 3641<br>P_O = 562
	**Aug 14**	N_P = 58<br>P_I = 4541<br>P_O = 0	N_P = 54<br>P_I = 3641<br>P_O = 0
All medical contacts	**Sept 13**	N_P = 68<br>P_I = 5305<br>P_O = 612*ƚ	N_P = 62<br>P_I = 4203<br>P_O = 1714*ƚ
	**Sept-Dec 13**	N_P = 65<br>P_I = 5097<br>P_O = 208	N_P = 59<br>P_I = 4021<br>P_O = 182
	**Sept 13-Aug 14**	N_P = 58<br>P_I = 4541<br>P_O = 556	N_P = 54<br>P_I = 3641<br>P_O = 380
	**Sept 14**	N_P = 57<br>P_I = 4411<br>P_O = 130	N_P = 54<br>P_I = 3641<br>P_O = 0

N_P = Number of practices

P_I = Patients in analysis

P_O = New patients out of analysis (withdrawals), when compared to previous period

* = In comparison to those in the experimental arm (n=5917)

** = In comparison to those in the control arm (n=6262)

ƚ = These figures include withdrawals & patients aged 4.

Control – Analysed:

		ITT	PP
Prescription uptake & scheduled medical contacts	**Aug 13**	N_P = 69<br>P_I = 5586<br>P_O = 676**ƚ	N_P = 69<br>P_I = 5586<br>P_O = 676**ƚ
	**Aug 13-Jul 14**	N_P = 54<br>P_I = 4549<br>P_O = 1037	N_P = 54<br>P_I = 4549<br>P_O = 1037
	**Aug 14**	N_P = 54<br>P_I = 4549<br>P_O = 0	N_P = 54<br>P_I = 4549<br>P_O = 0
All medical contacts	**Sept 13**	N_P = 69<br>P_I = 5586<br>P_O = 676**ƚ	N_P = 69<br>P_I = 5586<br>P_O = 676**ƚ
	**Sept-Dec 13**	N_P = 67<br>P_I = 5384<br>P_O = 202	N_P = 67<br>P_I = 5384<br>P_O = 202
	**Sept 13-Aug 14**	N_P = 54<br>P_I = 4549<br>P_O = 835	N_P = 54<br>P_I = 4549<br>P_O = 835
	**Sept 14**	N_P = 53<br>P_I = 4438<br>P_O = 111	N_P = 53<br>P_I = 4438<br>P_O = 111

For peer review only - http://bmjopen.bmj.com/site/about/guidelines.xhtml

For peer review only

Figure 2. Mean differences in medical contacts by time interval

a. Unscheduled medical contacts

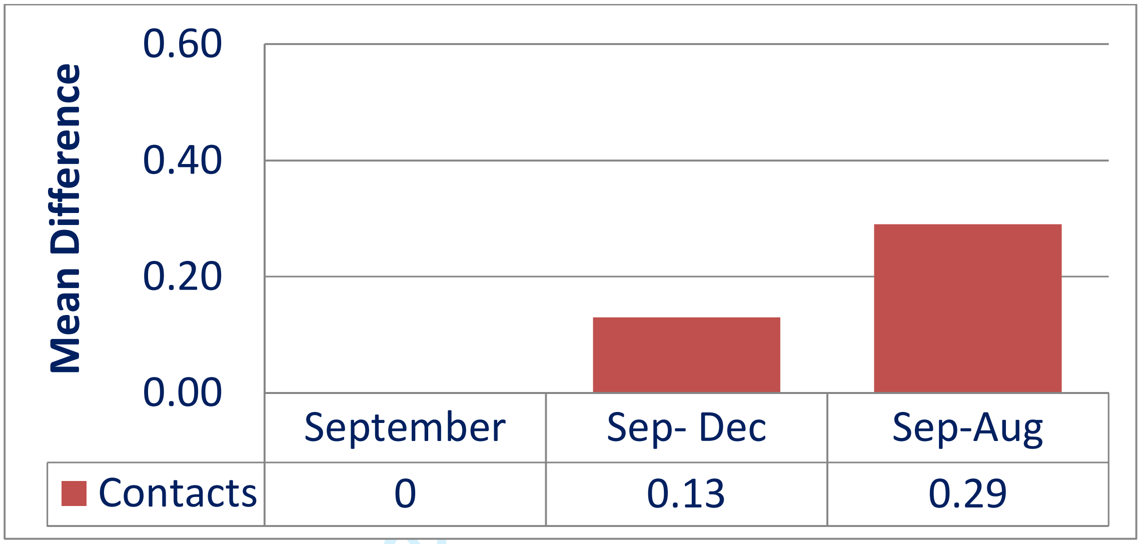

b. Total medical contacts

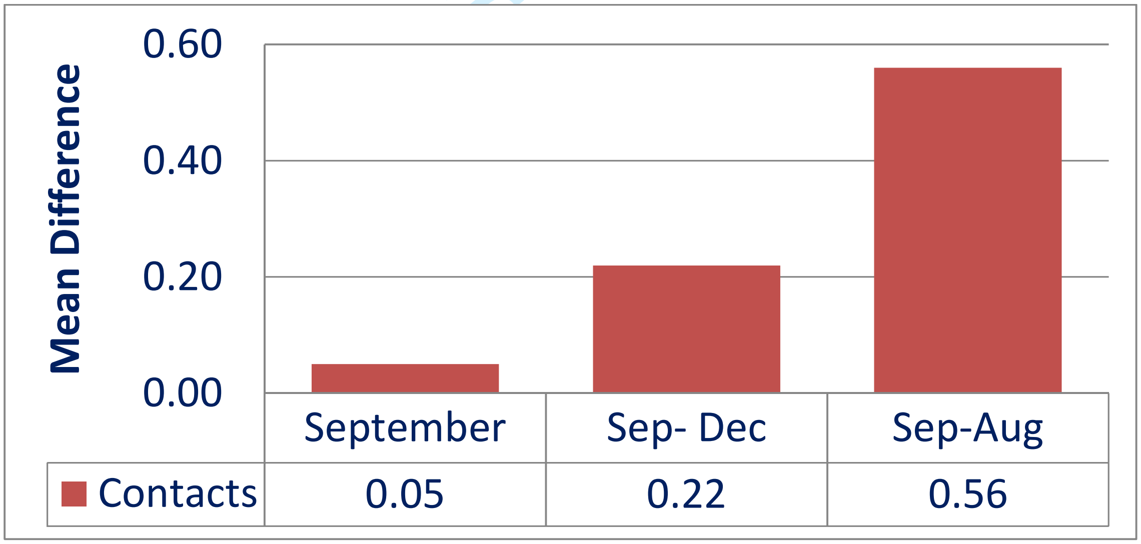

Table 1: CONSORT 2010 checklist of information to include when reporting a cluster randomised trial

Section/Topic	Item No	Standard Checklist item	Extension for cluster designs	Page No *
**Title and abstract**				
	1a	Identification as a randomised trial in the title	Identification as a cluster randomised trial in the title	1
	1b	Structured summary of trial design, methods, results, and conclusions (for specific guidance see CONSORT for abstracts)[1,2]	See table 2	2-8
**Introduction**				
**Background and objectives**	2a	Scientific background and explanation of rationale	Rationale for using a cluster design	4
	2b	Specific objectives or hypotheses	Whether objectives pertain to the the cluster level, the individual participant level or both	4
**Methods**				
**Trial design**	3a	Description of trial design (such as parallel, factorial) including allocation ratio	Definition of cluster and description of how the design features apply to the clusters	6
	3b	Important changes to methods after trial commencement (such as eligibility criteria), with reasons		**5**
**Participants**	4a	Eligibility criteria for participants	Eligibility criteria for clusters	5
	4b	Settings and locations where the data were collected		**8**
**Interventions**	5	The interventions for each group with sufficient details to allow replication, including how and when they were actually administered	Whether interventions pertain to the cluster level, the individual participant level or both	5
**Outcomes**	6a	Completely defined pre-specified primary and secondary outcome measures, including how and	Whether outcome measures pertain to the cluster level, the individual participant level or both	6

		when they were assessed		
	6b	Any changes to trial outcomes after the trial commenced, with reasons		**14**
**Sample size**	7a	How sample size was determined	Method of calculation, number of clusters(s) (and whether equal or unequal cluster sizes are assumed), cluster size, a coefficient of intracluster correlation (ICC or *k*), and an indication of its uncertainty	9
	7b	When applicable, explanation of any interim analyses and stopping guidelines		**N/A**
**Randomisation:**				
**Sequence generation**	8a	Method used to generate the random allocation sequence		**7**
	8b	Type of randomisation; details of any restriction (such as blocking and block size)	Details of stratification or matching if used	8
**Allocation concealment mechanism**	9	Mechanism used to implement the random allocation sequence (such as sequentially numbered containers), describing any steps taken to conceal the sequence until interventions were assigned	Specification that allocation was based on clusters rather than individuals and whether allocation concealment (if any) was at the cluster level, the individual participant level or both	8
**Implementation**	10	Who generated the random allocation sequence, who enrolled participants, and who assigned participants to interventions	Replace by 10a, 10b and 10c	8
	10a		Who generated the random allocation sequence, who enrolled clusters, and who assigned clusters to interventions	8
	10b		Mechanism by which individual participants were included in clusters for the purposes of the trial (such as complete	8

			enumeration, random sampling)	
	10c		From whom consent was sought (representatives of the cluster, or individual cluster members, or both), and whether consent was sought before or after randomisation	11
**Blinding**	11a	If done, who was blinded after assignment to interventions (for example, participants, care providers, those assessing outcomes) and how		**8-9**
	11b	If relevant, description of the similarity of interventions		**N/A**
**Statistical methods**	12a	Statistical methods used to compare groups for primary and secondary outcomes	How clustering was taken into account	9-11
	12b	Methods for additional analyses, such as subgroup analyses and adjusted analyses		**9-11**
**Results**				
**Participant flow (a diagram is strongly recommended)**	13a	For each group, the numbers of participants who were randomly assigned, received intended treatment, and were analysed for the primary outcome	For each group, the numbers of clusters that were randomly assigned, received intended treatment, and were analysed for the primary outcome	11
	13b	For each group, losses and exclusions after randomisation, together with reasons	For each group, losses and exclusions for both clusters and individual cluster members	11
**Recruitment**	14a	Dates defining the periods of recruitment and follow-up		**6**
	14b	Why the trial ended or was stopped		**N/A**
**Baseline data**	15	A table showing baseline demographic and clinical	Baseline characteristics for the individual and cluster levels as	24

For peer review only - http://bmjopen.bmj.com/site/about/guidelines.xhtml

		characteristics for each group	applicable for each group	
**Numbers analysed**	16	For each group, number of participants (denominator) included in each analysis and whether the analysis was by original assigned groups	For each group, number of clusters included in each analysis	22
**Outcomes and estimation**	17a	For each primary and secondary outcome, results for each group, and the estimated effect size and its precision (such as 95% confidence interval)	Results at the individual or cluster level as applicable and a coefficient of intracluster correlation (ICC or k) for each primary outcome	22
	17b	For binary outcomes, presentation of both absolute and relative effect sizes is recommended		**25-26**
**Ancillary analyses**	18	Results of any other analyses performed, including subgroup analyses and adjusted analyses, distinguishing pre-specified from exploratory		**25-26**
**Harms**	19	All important harms or unintended effects in each group (for specific guidance see CONSORT for harms[3])		**N/A**
**Discussion**				
**Limitations**	20	Trial limitations, addressing sources of potential bias, imprecision, and, if relevant, multiplicity of analyses		**13-15**
**Generalisability**	21	Generalisability (external validity, applicability) of the trial findings	Generalisability to clusters and/or individual participants (as relevant)	13-15
**Interpretation**	22	Interpretation consistent with results, balancing benefits and harms, and considering other relevant evidence		**13-15**
**Other information**				
**Registration**	23	Registration number and		**21**

For peer review only

		name of trial registry	
**Protocol**	24	Where the full trial protocol can be accessed, if available	**18, Ref 19**
**Funding**	25	Sources of funding and other support (such as supply of drugs), role of funders	**21**

** Note: page numbers optional depending on journal requirements*

For peer review only

BMJ Open

An Open-Label Cluster Randomised Controlled Trial and Economic Evaluation of a Brief Letter from a GP on Asthma Episodes Associated with the Start of the School Year – The PLEASANT Trial

Journal:	*BMJ Open*
Manuscript ID	bmjopen-2017-017367.R1
Article Type:	Research
Date Submitted by the Author:	11-Oct-2017
Complete List of Authors:	Julious, Steven; The University of Sheffield, Medical Statistics Group, ScHARR,<br>Horspool, Michelle; University of Sheffield, Medical Statistics Group, School of Health and Related Research (ScHARR),<br>Davis, Sarah; University of Sheffield, Health Economics and Decision Sciences, School of Health and Related Research (ScHARR)<br>Bortolami, Oscar; University of Sheffield, Medical Statistics Group, School of Health and Related Research (ScHARR)<br>Franklin, Matthew; University of Sheffield, Health Economics and Decision Sciences, School of Health and Related Research (ScHARR)<br>Smithson, Henry; University of Cork, Department of General Practice<br>Elphick, Heather; Sheffield Children's Hospital, Respiratory Department<br>Cooper, Cindy; University of Sheffield , Clinical Trials Research Unit, School of Health and Related Research (ScHARR)<br>Norman, Paul; University of Sheffield, Department of Psychology<br>Simpson, Rebecca; University of Sheffield , Medical Statistics Group, School of Health and Related Research (ScHARR)
**Primary Subject Heading**:	Respiratory medicine
Secondary Subject Heading:	Paediatrics, Public health, General practice / Family practice, Health economics, Medical management
Keywords:	Asthma < THORACIC MEDICINE, PRIMARY CARE, Cluster Trial, Randomised Controlled Trial, School-aged Children, Adherence

SCHOLARONE™
Manuscripts

An Open-Label Cluster Randomised Controlled Trial and Economic Evaluation of a Brief Letter from a GP on Asthma Episodes Associated with the Start of the School Year – The PLEASANT Trial

Authors: Prof Steven A Julious[a], Dr Michelle J Horspool[a], Sarah Davis[b], Dr Oscar Bortolami[a], Dr Matthew Franklin[b], Prof W Henry Smithson[c], Prof Heather Elphick[d], Prof Cindy Cooper[e], Prof Paul Norman[f], Rebecca M Simpson[a]

Author Institutions

[a] Medical Statistics Group, School of Health and Related Research (ScHARR), University of Sheffield, Sheffield, United Kingdom

[b] Health Economics and Decision Sciences, School of Health and Related Research (ScHARR), University of Sheffield, Sheffield, United Kingdom

[c] Department of General Practice, University of Cork, Cork, Ireland

[d] Respiratory Department, Sheffield Children's Hospital, Sheffield, United Kingdom

[e] Clinical Trials Research Unit, School of Health and Related Research (ScHARR), University of Sheffield, Sheffield, United Kingdom

[f] Department of Psychology, University of Sheffield, Sheffield, United Kingdom

Corresponding author:

Name: Prof Steven A Julious

Address: Medical Statistics Group, School of Health and Related Research, The University of Sheffield, Regent Court, Regent Street, Sheffield, S1 4DA

Tel: 0114 2220709

Fax: 0114 2220749

Email: s.a.julious@sheffield.ac.uk

For peer review only

ABSTRACT (300/300 words max)

Background: Asthma is seasonal with peaks in exacerbation rates in school-aged children associated with the return to school following the summer vacation. A drop in prescription collection in August is associated with an increase in the number of unscheduled contacts after the school return.

Objective: To assess whether a public health intervention delivered in general practice reduced unscheduled medical contacts in children with asthma.

Design: Cluster-randomised trial with trial-based economic evaluation. Randomisation was at general practice level, stratified by size of practice. The intervention group received a letter from their GP in late July outlining the importance of (re)taking asthma medication before the return to school. The control group was usual care.

Setting: General practices in England and Wales.

Participants: 12179 school-aged children in 142 general practices (70 on intervention).

Main Outcome: Proportion of children aged 5-16 who had an unscheduled contact in September. Secondary endpoints included collection of prescriptions in August and unscheduled contacts over 12 months. Economic endpoints were quality-adjusted life-years gained and health service costs.

Results: There was no evidence of effect (odds-ratio 1.09; 95% CI 0.96 to 1.25) on unscheduled contacts in September but the intervention reduced total contacts for the time intervals September-December and September-August. The mean number of total contacts per child over 12 months were reduced by 5%. The intervention increased the proportion of children collecting a prescription in August (odds-ratio 1.43; 95% CI 1.24 to 1.64).

The mean reduction in medical contacts informed the health economics analyses. The intervention was estimated to save £36.07 per patient - with a high probability (96.3%) of being cost-saving.

For peer review only

Conclusions: The intervention succeeded in increasing children collecting prescriptions and having scheduled contacts in August. It did not reduce unscheduled care in September (the primary outcome) but after September there was evidence in favour of the intervention.

Trial registration number: ISRCTN03000938

Key Words

Asthma, school-age children, primary care, cluster trial, general practice, randomised controlled trial, CPRD, unscheduled care, scheduled care, adherence

Funding details

This project was funded by the NIHR Health Technology Assessment programme

Strengths and limitations of this study

- The evaluation was a highly efficient study design using routine data to evaluate a general practice public health intervention designed with children with asthma and their parents
- The intervention was simple to implement had good user acceptability and was cost saving
- The intervention increased prescription uptake in the month prior to the return to school with 30% more prescriptions collected.
- There was no immediate effect in September but in the wide time intervals of September to December and September to August there was evidence of effect with a mean reduction in medical contacts
- The coding of the outcomes from the routine data did present challenges in the derivation of the outcomes with the assessment of adherence not possible.
-

For peer review only

1 INTRODUCTION

Asthma episodes and deaths are known to be seasonal [1]. A number of reports have shown peaks in asthma episodes in school-aged children associated with the return to school following the summer vacation [2–10]. Children returning to school are exposed to a variety of novel respiratory insults including allergens and viruses, at a time of changing climactic conditions. It has previously been shown that viral infection and allergen exposure in allergen sensitised asthmatics are associated with increased hospital admissions for acute asthma [11].

Our previous research [12] confirmed the increase in unscheduled medical contacts with children with asthma being approximately twice as likely as controls to have an unscheduled medical contact with their doctor around the time of the return back to school. In the same study it was found that in August, immediately preceding the return back to school, there were 25% fewer prescriptions for inhaled corticosteroids, compared to July and September. Furthermore, patients who received a prescription for inhaled corticosteroids were less likely to have an unscheduled medical contact after the return to school.

Little is known about the factors that are associated with the drop in prescriptions in August. Research on adherence to paediatric asthma treatment in general has identified weak beliefs about the necessity of asthma medication as a key reason for non-adherence[13]. Given that asthma symptoms decline in the summer months this may lead to weaker beliefs about the necessity to take asthma medication. The GP letter was designed to address this belief by emphasising the importance of (re)taking asthma mediation prior to returning to school.

The current study is a cluster randomised trial to evaluate whether a letter sent from a GP at the start of the summer vacation reminding parents of children with asthma of the necessity of taking their asthma medication before the return back to school. The study evaluated whether the letter reduced unscheduled contacts after the return back to school and increased prescriptions in August.

2 RESEARCH AIMS AND OBJECTIVES

The aim of the study was to assess if a general practice delivered public health intervention (a letter sent from the GP to parents/carers of school-aged children with asthma) can reduce the number of unscheduled medical contacts per child after the school return.

For peer review only - http://bmjopen.bmj.com/site/about/guidelines.xhtml

3 METHODS

3.1 Study Design

The study was an open-label cluster randomised trial where GP practices were randomised to the intervention or usual care. The study protocol and HTA report have been published [14 15]. The effectiveness of the intervention was assessed on the basis of reduced unscheduled medical contacts after the return to school in September and prescription uptake prior in August. The primary study period was 1st – 30th September 2013 after the return to school. The extended study period was 1st September - 31st December 2013, since asthma-related appointments are more frequent in these months for children with asthma. The full follow-up period was 12 calendar months from 1st September 2013 to 31st August 2014. Prescription uptake and scheduled medical contacts such as asthma reviews were evaluated during the periods August 2013 and August 2013-July 2014, respectively,

A cluster randomised trial was chosen due to the nature of the condition of asthma. Even if the study design was individually randomised there would have needed to be a need for the study to be randomised by household as siblings are likely to have asthma. A further consideration was that we wished for the intervention to represent possible routine care for future implementation. A practice level intervention would represent this.

The health economic analyses were based on a 12 month period from 1st August 2013 to 31st July 2014. The period starts a month earlier than the evaluation of medical contacts in order to incorporate the cost associated with delivering the intervention including any increase in prescriptions or medical contacts in response to the intervention that occurred during August 2013.

The primary outcome was the proportion of patients who had an unscheduled medical contact in September 2013.

The secondary outcomes evaluated included the number of unscheduled medical contacts in September 2013 and the number and proportion of any medical contacts (scheduled and unscheduled) in the same time interval as well as in the time intervals September-December

For peer review only - http://bmjopen.bmj.com/site/about/guidelines.xhtml

2013 and September 2013-August 2014. The analyses of the same outcomes were repeated for the other time intervals.

3.2 Participants

Participants were school-aged children with asthma, aged between 4 and 16, registered with a general practitioner. The primary analysis population was the intention to treat population (ITT) among children aged between 5 years and 16 years of age.

The choice of the 5-16 age group as the primary analysis population is due to the difficulty associated with making a diagnosis of asthma among children below this age [16,17]. Patients aged 4-5 were analysed separately to those aged 5-16 and are not included in the paper. Additional analyses were restricted specifically to children who had received a prescription for steroid inhalers in the previous year.

3.3 Interventions

Sites were randomly allocated to either: Intervention Group - sending out the letter or Control Group - standard care (no letter)

The intervention was a letter sent from a GP to the parents/carers of children with asthma reminding them to maintain their children's medication and collect a prescription if they were running low (See Appendix 1). It also advised that should their child have stopped their medication it should be resumed as soon as possible

The letter template was developed based on standard letters already used in general practice and designed to address beliefs about the necessity of taking asthma medication before the return back to school. The wording of the letter had input from the study team, which includes a GP, Health Psychologist and Consultant Respiratory Paediatrician and was also discussed in detail at two patient and public events, that included school-aged children with asthma and their parents[18 19 20].

The intervention letters were sent out the week commencing 29th July 2013 to obviate the distraction of planning for family holidays and yet leave enough time for parents and children to renew prescriptions and gain benefit from the medication. The letter and the timing of the

For peer review only - http://bmjopen.bmj.com/site/about/guidelines.xhtml

letter was decided following discussion with the Patients and Public Involvement (PPI) group [19].

3.4 Patient Involvement

There were three PPI consultation events with children with asthma and their parents. The first consultation event was funded by a grant by National Institute of Health Research (NIHR) Research Design Service for Yorkshire and the Humber prior to submission of the grant application in January 2011.

At this first consultation event it was agreed that a letter from their practice would be a useful reminder and not seen in any way as intrusive. A draft of the proposed letter was reviewed and the children fed back that they believed that the letter from their GP should be addressed to their parents rather than to themselves.

The second PPI consultation event was held after the grant was awarded in September 2012 [19]. At this meeting the intervention letter was finalised. The general feeling among the group was that the intervention did not adequately reflect the seriousness of asthma as a health condition. It was felt therefore that there was a danger that the intervention could be ignored by parents, or that the information it contained could be forgotten about. The letter was amended to reflect this input.

The consultation event also discussed the timing of the intervention and it was proposed to send the intervention the first week of August. The event also reviewed the lay summary for the study and provided input to the logo for the study.

Two parents also agreed to join the trial steering committee for the study. At the first trial steering committee meeting it was agreed to bring the timing of the intervention forward by a week to the end of July as asthma medication has a better chance of working the earlier they are used consistently.

A third PPI consultation event was held after the study had been completed which will be discussed in the discussion[21]. There is a web site where the PPI events are detailed

For peer review only

(http://www.sheffield.ac.uk/scharr/sections/dts/ctru/pleasant/ppi Assessed 3 October 2017). There has also been a separate publication on the first two PPI consultation events.[20]

3.5 Ethical approval and research governance

Ethical approval for the study was given by South Yorkshire Research Ethics Committee on 25th October 2012 (reference number 12/YH/04). NHS Permissions to conduct the study was obtained for all the Primary Care Trusts (PCTs) in England and Health Boards in Wales. Details of an amendment to the protocol are given in Appendix 2.

The trial was registered with the International Standard Randomised Controlled Trial Register (ISRCTN) reference number ISRCTN 03000938.

3.6 Setting

The setting was primary care with the unit of cluster being general practices. Site eligibility required practices to be using the Vision IT software and be part of Clinical Practice Research Datalink (CPRD). Site recruitment was conducted by CPRD and the NIHR Primary Care Research Network with the PLEASANT study team[22].

3.7 CPRD recruitment

A practice recruitment pack, consisting of a detailed study information sheet and an expression of interest (EoI) form, was sent to all 433 practices contributing to CPRD in England and Wales at the time of recruitment[22]. Practices were also recruited through the primary care research network. Recruitment took place over a 7-month period from January 2013 to July 2013. For these practices to be in the trial they needed to join the CPRD.

3.8 Randomisation and blinding

After each practice gave verbal consent to participate in the trial they were randomised to either the intervention or usual care.[22] Randomisation was stratified by size of General Practice (i.e. the “list size”) to ensure that there was an equal sample size – in terms of number of school-age children with asthma – in each arm of the trial. The randomisation sequence was generated by a statistician based within the Sheffield CTRU, using a blocked randomisation and allocation concealment was ensured by restricting access to the two CTRU statisticians. Once practices had agreed to participate, their identifier and list size was

For peer review only

forwarded to the trial statistician for randomisation to one of the two groups. The randomisation was then revealed to the study manager and research assistant. The study team were unblinded throughout the study but had no access to data until after a statistical analysis plan was developed and had no influence on data capture.

3.9 Data management

Data was collected through the CPRD which captures the coding for each consultation by staff in the practice. The medical consultations and diagnostic codes were reviewed to determine if each contact was a scheduled contact – such as a medicines review – or an unscheduled contact – such as an acute or an out of hours visit.

An independent GP adjudication panel was established to help in the coding. The adjudication panel met three times and did not have access to the randomisation group when reviewing the data. The adjudication panel reviewed and coded 4,600 unique terms into scheduled and unscheduled medical contacts. These terms accounted for 92% of all medical contacts but 17% of all terms used in the study. Terms not coded by the adjudication panel were coded as unscheduled. In addition, 7.9% of all contacts did not have any terms to indicate consultation type or diagnosis and free text was used in the database system to which the study team had no access. The adjudication panel advised to code these contacts as unscheduled. The GP adjudication panel did not have access to the randomisation group when reviewing the data.

3.10 Statistical methods

3.10.1 Analysis populations

The study was designed to detect a difference of 5% in the proportion of children who have an unscheduled medical contact (30 v 25%) with 90% power and two sided significance level of 5%, with an intra-class correlation (ICC) of 0.03 to account for clustering. Based on this we estimated that we required 70 practices per arm. It was anticipated that the sample size of 140 practices would equate to approximately 14,000 school-age children with asthma. We assumed equal cluster sizes in the sample size calculation. Sensitivity analyses indicated that the study was robust the assumptions made for the ICC as well as to practices not sending the intervention and reducing the observed effect size [15].

For peer review only - http://bmjopen.bmj.com/site/about/guidelines.xhtml

Each of the outcomes were evaluated on each of subpopulations: children aged 5-16 (the primary analysis population) and children aged 5-16 who have prescriptions for steroid preventer. The analyses were restricted specifically to children who had received a prescription for preventer inhalers in the previous year as this was intended to identify the treatment effect in the population likely to receive most benefit.

The primary analyses of effectiveness were performed on the intention to treat (ITT) population. Analyses were also conducted on the per protocol (PP). The health economic analyses were based on the PP population. ITT analyses comprised all practices for whom data were obtained for the study period (see Section 4.3). The two criteria for exclusion from PP analyses were: practices that did not send intervention as requested. In such cases, the entire practice data was excluded from PP analyses and individual children who were not sent the intervention letter. GPs were given discretion to withhold the letter from any children they believed were unsuitable. In such cases, the individual was excluded from PP analyses.

3.10.2 Analytical methods

The proportion of children having an unscheduled medical contact was analysed separately for each time period using logistic regression with the individual's age, sex, number of contacts the previous September as covariates, the trial arm (intervention or control) as a fixed effect, and the design/cluster effect of general practice as a random effect. The proportion of children having a prescription within each time period was analysed in the same manner. The number of unscheduled medical contacts made in each period by the children as well as the number of prescriptions ordered within a time period, were both analysed using a random effects negative binomial model in which the same covariates as above were included. [15]

3.11 Health economic methods

An economic evaluation was undertaken to compare the incremental cost per quality adjusted life year (QALY) of the reminder letter versus standard care. The perspective of the analysis was that of the NHS (primary and secondary care resource-use based on available CPRD data and associated costs). We assumed the intervention would have no impact on quality of life (utility) beyond 4 months or mortality so QALYs were calculated for the four month post-

For peer review only

intervention time period. Costs were calculated for one year post-intervention to allow for changes in the timing of routine asthma care in response to the intervention to be distinguished from changes in the number of scheduled contacts.

Bootstrapped costs were evaluated 12 months post-intervention with one year linear regression-based baseline adjustment (BA).

Costs for the letter intervention were based on a survey of participating practices which included questions on staff members involved as well as staff time[23].

Full details of the methods used in the economic analysis have been published in a separate paper [24].

3.12 Trial oversight

A Trial Steering Committee (TSC) was established to give oversight to the study. The TSC consisted of an independent chair (GP), two independent members (academic GP and statistician) and two lay members (parents of children with asthma) along with the Principal Investigator and key staff within the CTRU (as non-voting members). The role of the TSC was to provide supervision of the protocol, statistical analysis plan and to provide advice on and monitor progress of the trial.

4 TRIAL RESULTS

4.1 Recruitment and participant flow

The target sample size was 140 GP practices. In total, 142 practices agreed to take part in the study. Recruitment of GP practices was undertaken over a 7 month period, details of which have been published[22]. Of these practices, one (a control group practice with 99 children with asthma) withdrew consent after the start of the study for the data to be extracted and stored by the CPRD (independent of the study); this practice was excluded from all analyses. In total, 70 practices (comprising 5917 individuals) were randomised to the intervention (letter), and

For peer review only

71 practices (6262 individuals) to control. The CONSORT diagram is given in Figure 1 for the 12 months follow-up of the study.

4.2 Baseline characteristics

The descriptive statistics of the 12179 subjects and 141 practices are included in Table 1a and Table 1b. Summaries reported are stratified by intervention type and overall.

4.3 Number of participants and analysis subsets

For each study period, analyses were based only on practices that contributed data to the entirety of that period. In other words, if practices stopped submitting data to CPRD before the end of a given follow-up period they were excluded from all analyses for that time period. Practices that changed their software from the Vision IT system were no longer able to participate in CPRD and so withdrew from the study. Details of the practices within the study during each time period are given in Appendix 3.

Figure 1 shows the flow of subjects for the primary analysis population (aged 5-16). Of the 456 practices invited, 433 were through the CPRD and 23 were through the primary care research network and joined the CPRD[22].

There were 786 GP exclusions in the intervention arm. There were zero GP exclusions in the control arm, as it was impossible for the GPs to exclude individuals from receiving letters when no patients in the control arm were due to receive a letter.

For peer review only

4.4 Clinical results

The unscheduled medical contacts for children in the trial are given in Figure 2a. In this figure we have the unscheduled medical contacts in the year leading up to the intervention and post the intervention. We can see from this figure in 2012 the pronounced drop in unscheduled medical contacts in August. After the return to school in September there is an increase in unscheduled medical contacts which peaks in October/November before reducing.

In 2013 there is a similar pattern to 2012 but now after the intervention has been sent there is seems to be no immediate effect of the intervention in September and the peak in October/November is less pronounced than compared to the no letter arm.

The primary time point for the analysis was September. Thus, in the primary analysis the proportion of individuals who had at least one unscheduled medical contact in September was 45.2% in the intervention arm, compared with 43.7% in the control arm (adjusted odds ratio (OR) = 1.09, 95% CI 0.96 to 1.25 (see Table 2). The ICC for the primary analysis was 0.026 and was consistent with the estimate for the sample size calculation. In terms of mean contacts the number of unscheduled contacts are comparable (incidence rate ratio IRR=1.02 95% CI 0.94 to 1.12). The results are comparable for children receiving preventer medication (see Table 2b).

As highlighted in Figure 2a after September there was evidence of a reduction in the mean number of medical contacts. As a consequence, the incidence ratio declines as longer time periods are analysed (see Table 2) suggesting that the short-term increase in medical contacts in September is gradually outweighed by decreases in unscheduled contacts in the longer-term. For unscheduled contacts over 12 months there is a reduction of 3% (IRR=0.97 with 95% CI 0.95 to 1.04)

The total number of medical contacts in the twelve months is reduced by 5% (IRR=0.95 with 95% CI 0.91 to 0.99).

For peer review only

Similar to Figure 2a the preventer prescription collections for children in the trial are given in Figure 2b. In this figure we have the number of preventer prescription collections in the year leading up to the intervention and post the intervention. Mirroring the unscheduled contacts in August 2012 there also is a reduction in prescriptions collected in this month. After the return to school in September there is an increase in prescriptions collected with a peak in the interval October to December followed by a reduction.

In 2013 there is a similar pattern to 2012 but in 2013 after the intervention has been sent there is a marked increase in prescriptions in August in the intervention arm. The increase seems to continue into September and October.

The planned analysis was of prescriptions in August. This demonstrated that the intervention (letter) was associated with an increased uptake of prescriptions in the month of August 2013 – see Table 3. In August, 876 (16.5%) patients in the intervention arm had at least one prescription compared with 703 (12.6%) in the control group (adjusted odds ratio 1.43, 95% CI 1.24 to 1.64); the total number of prescriptions was also higher (adjusted incidence rate ratio 1.31, 95% CI 1.17 to 1.48).

Scheduled contacts made in August 2013 also increased (adjusted odds ratio 1.13, 95% CI 0.84 to 1.52).

In the 12 months from September the odds ratio for prescriptions was 1.06 (95% CI 0.94 to 1.19), whereas for scheduled contacts it was 0.89 (95% CI 0.69 to 1.16). For scheduled contacts these results could be suggestive of re-timing of contacts which would have happened as part of the planned care of the children.

The effect observed of an increase in prescription uptake in September and October in intervention arm came from the data and was not formally statistically tested.

For peer review only

There is evidence of user acceptability of the intervention with over half the practices in the intervention (13 out of 24) who responded to a survey saying they repeated the intervention the year following the study[23]

The analysis of respiratory relation contacts is given in Appendix 4

4.5 Health economic results

The full results of the economic evaluation have been published in a separate paper so only key BA base-case results are provided here[24]. The average cost per child of sending the intervention was £1.34 per child. The fall in medical contacts over one year described in the clinical results led through into the health economic assessment. A mean reduction in costs per child of £36.07 was estimated and there was 96.3% certainty of the intervention being cost saving. The economic evaluation estimated a mean QALY loss of 0.00017 which is practically zero.

5 DISCUSSION

Previous work has shown an increased in the number of unscheduled medical contacts by children in autumn months (September to December), which may be due to the start of the new school term [25]. By sending a letter at the start of the school holidays to remind children of the importance of taking their medication, it was hypothesised that the increase may be averted. More specifically, it was predicted that a reminder letter would lead to a greater uptake of inhaler prescriptions in August that, in turn, would lead to increased adherence and, finally, fewer unscheduled medical appointments.

There was evidence of an impact on the first part of this pathway as the intervention group demonstrated a higher uptake of prescriptions in August 2013. They also had an increase in the number of scheduled contacts in the same month. The data is not available to confirm actual medicine usage and so it is unclear whether the increased uptake also translated into an increased usage.

The original plan was to assess this through the medicines possession ratio – which estimates the time a child has collected medication for over the time the child should have collected

For peer review only

time for. This could not be estimated for these data however due to the inadequate recording in the routine data of the prescription data. Our analysis of the PLEASANT dataset suggests that further work is required to determine how to assess adherence using such data.

The primary endpoint was unscheduled medical contacts in September 2013, which coincided with the start of the new school term. There was no evidence of a reduction in the intervention group. In fact there was an increase in the proportion of children who had an unscheduled medical contact in September.

The increase could be caused by GPs needing to see certain patients before giving a new prescription if they had not had a prescription recently. Evidence to support this is a *post hoc* observation that for children who had collected a prescription within the last 3 months prior to the start of the study there was less evidence of an increase in unscheduled contacts in September - 55.2% of patients in the intervention arm seeing their GP compared to 54.3% for controls. This is compared patients whose last prescription was 3-6 months prior to the start of the study where difference between the arms was greater - 42.1% in the intervention arm seeing their GP in September against 39.7% on control.

The way the unscheduled contacts were coded could have also impacted on the outcome. The intervention increased prescription update and collection of a prescription for asthma was used in the definition of an unscheduled medical contact.

A further explanation is that September was too early to make an assessment of efficacy. Given that exposure to infections and the impact on asthma may take some time to have an impact on school-age children, it is possible that making the primary outcome period the first 4 weeks after returning to school was too soon to observe an effect of the intervention. It is interesting that an effect was demonstrated when the measurement period was extended to December. In the extended period both the total number of contacts per child (i.e. scheduled plus unscheduled) and unscheduled contacts were lower in the intervention group than in the control over the extended study period (September to December 2013) and the full year (September 2013 to August 2014). Although the effects were not statistically significantly, the minimal cost associated with the intervention meant the intervention was found to have a high probability of being cost-saving overall.

For peer review only

With such a relatively low cost intervention, £1.34 per child, and an average cost for an unscheduled surgery visit circa £50, an intervention would only need to reduce the number of contacts by 3 per year for an average practice with 85 asthmatic children to be cost neutral. The evidence from the trial is that contacts are reduced 0.6 per child in the 12 months after intervention, or 51 per year for an average practice of 85 children.

The economic analysis (which used data over a 12 month period from August 2013 to July 2014) estimated a mean cost saving across the base case of £36.07 per child. So, although the study did not have a significant effect for the primary endpoint, the minimal cost associated with the intervention meant the intervention was found to have a high probability (96.3%) of being cost-saving overall. In the UK alone there are over one million children with asthma. The intervention thus has potential to provide health service savings if implemented.

The results were discussed with children with asthma and their parents at a PPI consultation event[21]. At the event attendees felt that the savings per child was an important result and suggested that the impact of the intervention could have been greater if it had been repeated over a number of years. The letter could then assist parents and children as they plan for the school return each year.

There is evidence of good user acceptability with over half the practices who responded to a survey reported that they repeated the intervention the year after the study. Once the intervention is set up for one year the costs then associated with sending it out are less due to many of the school age children with asthma being the same from year to year.

There were methodological issues associated with a cluster randomise trial. Although there were 12,179 children with asthma in the study there were 141 GP practices which was the unit of randomisation. With 141 GP practices there is a chance of random differences between the two intervention arms. Any random differences could be compounded by the fact with common medical practice undertaken within a GP practice children with asthma would tend to be more alike within practices than between practices. This may affect the clinical outcomes.

The strengths of the study were that the intervention was evaluated in a relatively large trial population of children within a primary care setting within a single year. In addition the

For peer review only

procedures used in the study were the same as those that would be used in clinical practice and so implementation into routine care is straightforward.

The study had a highly efficient innovative study design that used routine data for all outcomes and the delivery of the intervention was centrally automated through the CPRD. By our own estimation a substantial six figure sum is saved compared to a trial where GP practices would need to be visited to collect the data.

There were additional practical advantages in using routine data. For example, the planning of data collection could be relatively straightforward to schedule and the collection of baseline data could be done retrospectively once the practice had entered the trial.

This final strength of the trial is also a weakness. Using routine data made the assessment of unscheduled contacts within the trial difficult - especially for an intervention which increased initial medical activity through the collection of prescriptions. In this study, it would have been helpful for two additional questions to be asked to facilitate evaluation of the intervention: Was the contact unscheduled? Was the contact respiratory related?

The study adds to the current literature by demonstrating that an easy to implement intervention of a simple letter from a GP to the parents of a children with asthma can assist in the self-management of condition by raising awareness of the importance of taking regular medication and by raising awareness of the importance of taking regular medication and by increasing prescription uptake and consequently reducing medical contacts. Over 90% of medical contacts are in primary care setting and yet there is a paucity of evidence of evaluations in this setting. This trial will add to this literature. It has also demonstrated that using routine data collected through the CPRD is a feasible in randomised trials and has shown the advantages and disadvantages of this approach

6 CONCLUSIONS

The intervention succeeded in increasing the number of children collecting a prescription in August, along with the proportion of children who had scheduled contacts in the same month.

The intervention did not reduce unscheduled care as expected in September, which was the primary endpoint, although over a longer time period there is evidence that the intervention

For peer review only

reduced medical contacts. This is reflected in the health economic evaluation which overall showed that the intervention had a high probability of giving a cost saving.

With the evidence from the trial of an increase in August of both prescription collection and evidence of cost reduction practices may wish to implement the intervention, particularly practices with high rates of unscheduled medical care.

7 REFERENCES

1. Campbell, M. J., Holgate, S. T. & Johnston, S. L. Trends in asthma mortality. *BMJ* **315,** 1012 (1997).
2. Storr, J, Lenney, W. School holidays and admissions with asthma. *Arch. Dis. Child.* **64,** 103–7 (1989).
3. Grech, V., Balzan, M. & Distefano, S. Paediatric Wheezy Admissions at and around School Holiday Periods. *Malta Med. J.* **16,** 23–26 (2004).
4. Kimes, D, Levine, E, Timmins, S, Weiss, SR, Bollinger, ME, Blaisdell, C. Temporal dynamics of emergency department and hospital admissions of pediatric asthmatics. *Environ. Res.* **94,** 7–17 (2004).
5. Kimbell-Dunn, M., Pearce, N. & Beasley, R. Seasonal variation in asthma hospitalizations and death rates in New Zealand. *Respirology* **5,** 241–6 (2000).
6. Gergen, P. J., Mitchell, H. & Lynn, H. Understanding the seasonal pattern of childhood asthma: results from the National Cooperative Inner-City Asthma Study (NCICAS). *J. Pediatr.* **141,** 631–6 (2002).
7. Harju, T., Keistinen, T., Tuuponen, T. & Kivelä, S. L. Seasonal variation in childhood asthma hospitalisations in Finland, 1972-1992. *Eur. J. Pediatr.* **156,** 436–9 (1997).
8. Julious, S. A., Osman, L. M. & Jiwa, M. Increases in asthma hospital admissions associated with the end of the summer vacation for school-age children with asthma in two cities from England and Scotland. *Public Health* **121,** 482–4 (2007).
9. Silverman, R. A., Stevenson, L. & Hastings, H. M. Age-related seasonal patterns of emergency department visits for acute asthma in an urban environment. *Ann. Emerg. Med.* **42,** 577–586 (2003).
10. Fleming, D. M., Cross, K. W., Sunderland, R. & Ross, A. M. Comparison of the

For peer review only

seasonal patterns of asthma identified in general practitioner episodes, hospital admissions, and deaths. *Thorax* **55,** 662–5 (2000).

11. Murray, CS, Poletti, G, Kebadze, T, Morris, J, Woodcock, A, Johnston, S. & Custovic, A. Study of modifiable risk factors for asthma exacerbations: virus infection and allergen exposure increase the risk of asthma hospital admissions in children. *Thorax* **61,** 376–82 (2006).

12. Julious, S. A., Campbell, M. J., Bianchi, S. M. & Murray-Thomas, T. Seasonality of medical contacts in school-aged children with asthma: association with school holidays. *Public Health* **125,** 769–76 (2011).

13. Drotar, D; Bonner, M. Influences on Adherence to Pediatric Asthma Treatment: A Review of Correlates and Predictors. *J. Dev. Behav. Pediatr* **30,** 574–82 (2009).

14. Julious SA, Horspool MJ, Davis S, Bradburn M, Norman P, Shephard Neil, Cooper CL, Smithson, WH, Boote J, Elphick Heather, Loban A, Franklin M, Kua WS, May Robin,; Campbell, Jennifer; Williams, Rachael; Rex, Saleema; Bortolami, O. PLEASANT: Preventing and Lessening Exacerbations of Asthma in School-age children Associated with a New Term - a cluster randomised controlled trial and economic evaluation. *Health Technol. Assess. (Rockv).* **20,** 1–154 (2016).

15. Horspool, M. J. *et al.* Preventing and lessening exacerbations of asthma in school-age children associated with a new term (PLEASANT): study protocol for a cluster randomised control trial. *Trials* **14,** 297 (2013).

16. British Thoracic Society / Scottish Intercollegiate Guideline. *British Guideline on the Management of Asthma: A national clinical guideline.* (2012).

17. Bush, A. Diagnosis of asthma in children under five. *Prim. Care Respir. J.* **16,** 7–15 (2007).

18. Julious SA, Lai J, Boote J, Elphick H, S. H. Report of a patient and public involvement consultation event with children with asthma and their parents to review a proposal for a primary care intervention around the September school return. *Univ. Sheff. ScHARR Rep. Ser. ISBN 1 900752 18 2* **25,** ISBN 1 900752 18 2 (2011).

19. Julious, SA, Horspool M J, Boote, J. PLEASANT TRIAL: 2nd Consultation event with children with asthma and their parents. *Univ. Sheffield. ScHARR Rep. Ser. No 28. ISBSN 1 900752 14 X* (2012).

For peer review only

20. Boote, J, Julious, S, Horspool, M, Elphick, H, Smithson, WH, Norman, P. PPI in the PLEASANT trial: involving children with asthma and their parents in designing an intervention for a randomised controlled trial based within primary care. *Prim. Health Care Res. Dev.* 1–13 (2016). doi:10.1017/S1463423616000025

21. Simpson, RM, Julious, SA, Mooney, C, Norman, P. PLEASANT TRIAL: 3rd Consultation event with children with asthma and their parents. *Univ. Sheff. ScHARR Rep. Ser. No 32. ISBN 1900752344.* (2015).

22. Horspool, MJ, Julious, SA, Mooney, C, May, R, Sully, B, Smithson, W. Preventing and Lessening Exacerbations of Asthma in School-aged children Associated with a New Term (PLEASANT): Recruiting Primary Care Research Sites-the PLEASANT experience. *NPJ Prim. care Respir. Med.* **25,** 15066 (2015).

23. Hatfield, I., Julious, S.A., Davis, S., Horspool, M.J., Norman, P., Mooney, C. An assessment of the resources used by General Practices in the intervention arm of the PLEASANT study in sending out the intervention. *Univ. Sheffield. ScHARR Rep. Ser. No 30. ISBSN 1 900752 24 7* (2015).

24. Franklin, M., Davis, S., Horspoo, l M., Kua, W.S., Julious, S. Economic Evaluations Alongside Efficient Study Designs Using Large Observational Datasets: the PLEASANT Trial Case Study. *Pharmacoeconomics* **35,** 261–73 (2017).

25. Julious, S. A., Campbell, M. J., Bianchi, S. M. & Murray-Thomas, T. Seasonality of medical contacts in school-aged children with asthma: association with school holidays. *Public Health* **125,** 769–76 (2011).

26. Yong, Y. V. & Shafie, A. A. Economic evaluation of enhanced asthma management: A systematic review. *Value Heal.* **16,** A377 (2013).

For peer review only

Study Registration

ISRCTN03000938 - assigned 19/10/2012

Contributors

Steven A. Julious, Professor Medical Statistics; Michelle J Horspool, Trial Manager; Sarah Davis, Health Economist; Matthew Franklin, Health Economist, together produced the first draft of the report.

The following conceived of or designed the work: Steven A. Julious, Professor of Medical Statistics; Michelle J Horspool, Trial Manager; Sarah Davis, Health Economist; W Henry Smithson, Prof. Primary Care; Healther Elphick, Consultant Respiratory Paediatrician; Paul Norman, Prof Health Psychology; Cindy L Cooper, Sheffield CTRU Director.

The following were involved in the interpretation of data for the work: Steven A Julious, Prof Medical Statistics; Michelle Horspool, Trial Manager; W Henry Smithson; Prof. Primary Care; Healther Elphick, Consultant Respiratory Paediatrician.

The following were involved in the analysis of data: Oscar Bortolami, Medical Statistician; Sarah Davis, Health Economist; Matthew Franklin, Health Economist and Rebecca Simpson, PhD student.

The following drafted the paper: Steven A. Julious, Professor Medical Statistics; Michelle J Horspool, Trial Manager; Sarah Davis, Health Economist; Matthew Franklin, Health Economist.

The following reviewed the work critically for important intellectual content. Steven A. Julious, Professor Medical Statistics; Michelle J Horspool, Trial Manage, Oscar Bortolami, Medical Statistician; Sarah Davis, Health Economist; Matthew Franklin, Health EconomistW Henry Smithson, Prof Primary Care; Heather Elphick, Consultant Respiratory Paediatrician; Paul Norman, Prof of Health Psychology; Cindy L Cooper, CTRU Director.

All authors agree to be accountable for all aspects of the work in ensuring that questions related to the accuracy or integrity of any part of the work are appropriately investigated and resolved.

Declaration of interests

We have read and understood BMJ policy on declaration of interests and declare that we have no competing interests.

For peer review only

All authors have completed the ICMJE uniform disclosure form at www.icmje.org/coi_disclosure.pdf and declare: no support from any organisation for the submitted work; no financial relationships with any organisations that might have an interest in the submitted work in the previous three years; no other relationships or activities that could appear to have influenced the submitted work.

Funding for this study was provided by the Health Technology Assessment programme of the National Institute for Health Research Programme (Grant 11/01/10)

Exclusive License

The Corresponding Author has the right to grant on behalf of all authors and does grant on behalf of all authors, a worldwide licence to the Publishers and its licensees in perpetuity, in all forms, formats and media (whether known now or created in the future), to i) publish, reproduce, distribute, display and store the Contribution, ii) translate the Contribution into other languages, create adaptations, reprints, include within collections and create summaries, extracts and/or, abstracts of the Contribution, iii) create any other derivative work(s) based on the Contribution, iv) to exploit all subsidiary rights in the Contribution, v) the inclusion of electronic links from the Contribution to third party material where-ever it may be located; and, vi) licence any third party to do any or all of the above

Transparency declaration

The lead author, and manuscript guarantor, affirms that this manuscript is an honest, accurate, and transparent account of the study being reported; that no important aspects of the study have been omitted; and that any discrepancies from the study as planned (and, if relevant, registered) have been explained.

Data Sharing

Access to patient level data is provided by the CPRD for health research purposes and is dependent on approval of a study protocol by the MHRA Independent Scientific Advisory Committee (ISAC). More information on ISAC and the protocol submission process can be found at: www.cprd.com/isac (date accessed 18 April 2017).

For peer review only

Sponsor

The study was sponsored by Sheffield Clinical Commissioning Group. The authors has freedom to act in the writing and submission of the paper.

Acknowledgments

We gratefully acknowledge the hard work, support and advice from the following: Hilary Pinnock, (Professor, University of Edinburgh) for her advice; Gerry McCann, Zaynah Gurreebun, Rachael Williams, Robin May and Jennifer Campbell, Clinical Practice Research Datalink (Clinical Practice Research Datalink, CPRD) for their contribution to site recruitment and to Tjeer Van Staa (CPRD) for his advice on CPRD; Cara Mooney, David White (University of Sheffield) for support with study set up, site recruitment and site set up and close down; Dan Beever and Helen Wakefield for administrative and clerical support; Wei Sun Kua, for her Health Economic contribution; Jonathan Boote, as PPI Lead; Mike Bradburn and Neil Shephard for the statistical input and Amanda Loban for data management.

We are very grateful to the children and parents who took part in the three patient and public events through all stages of the study for their advice and input.

We would like to especially thank the GP adjudication panel Dr Mark Boon (Conisbrough Group Practice), Dr Karen Forshaw (Bentley Surgery), Dr Julie Hackney (The Avenue Surgery) for their advice, steer and valued contributions.

We offer special thanks to the members of our oversight committee: Trial Steering Committee: Dr Steve Holmes (Independent Chair, General Practitioner), Prof Andrew Wilson (Independent Academic General Practitioner, University of Leicester), Dr Martyn Lewis (Independent Statistician, Keele University), Zaida Bibi (Independent Parent Representative), and Camilla Mills (Independent Parent Representative).

The views expressed in this report are those of the authors and not necessarily those of the National Institute for Health Research Health Technology Assessment programme. Any errors are the responsibility of the authors.

For peer review only

Trial Summary

What is already known about this topic? We have not identified any other studies which have examined the economic benefits of a simple postal intervention in asthma patients and therefore it is difficult to compare our results to those of existing published studies. Yong and Shafie [26] have published a systematic review which looked more broadly at non-pharmacological interventions that aimed to enhance asthma management. The interventions included by Yong and Shafie varied from educational and self-management interventions to environmental interventions. Whilst the PLEASANT intervention letter could be considered to be a simple form of patient education, the educational interventions included by Yong and Shafie were all more intensive than the postal intervention used in PLEASANT, and the population was not restricted to school-aged children, making comparisons difficult. However, the broader evidence reviewed by Yong and Shafie suggests that non-pharmacological interventions which aim to improve an individual's management of their asthma have the potential to be cost-effective.

What are the new findings? The intervention in PLEASANT caused an increase in prescription collection in August as well as scheduled medical contacts. It did not then reduce medical contacts in September but after September there was evidence of a fall in medical contacts which followed through in the economic analysis to give a high probability of the intervention being cost-saving.

How might this influence practice? The increase in prescriptions and scheduled contacts in August could lead to individual GP practices wishing to implement the intervention. Evidence from the trial suggests this would decrease overall costs associated with the asthma management and reduce unscheduled care.

For peer review only - http://bmjopen.bmj.com/site/about/guidelines.xhtml

Figure 1. CONSORT Diagram

Figure 2. Unscheduled medical contacts and prescriptions over time

a. Unscheduled medical contacts
b. Prescriptions for preventer medication

Figure 3. Unscheduled respiratory medical contacts

For peer review only

Table 1: Descriptive statistics of patients and surgeries

a) Descriptive statistics of sex (frequencies and percentages reported) and age (mean, SD, median, interquartile range and range reported). Statistics produced at subject level.

Variable	Letter (N=5917)	No Letter (N=6262)	Total (N=12179)
Sex	Male (%)<br>3505 (59.24)<br><br>Female (%)<br>2412 (40.76)	Male (%)<br>3749 (59.87)<br><br>Female (%)<br>2513 (40.13)	Male (%)<br>7254 (59.56)<br><br>Female (%)<br>4925 (40.44)
Age	Mean (SD)<br>10.51 (3.29)<br>Median (IQR)<br>10.80 (7.88 - 15.97)<br>Range<br>4.05 - 15.97	Mean (SD)<br>10.55 (3.30)<br>Median (IQR)<br>10.89 (7.80 - 15.97)<br>Range<br>4.05 - 15.97	Mean (SD)<br>10.53 (3.30)<br>Median (IQR)<br>10.89 (7.80 - 15.97)<br>Range<br>4.05 - 15.97

b) Descriptive statistics of size (mean, SD, median, interquartile range and range reported). Statistics produced at surgery level.

Variable	Letter (N=70)	No Letter (N=71)	Total (N=141)
Size	Mean (SD)<br>85 (44)<br>Median (IQR)<br>80 (49 - 114)<br>Range<br>4-209	Mean (SD)<br>88 (64)<br>Median (IQR)<br>75 (41 - 107)<br>Range<br>10-293	Mean (SD)<br>86 (55)<br>Median (IQR)<br>76 (45-113)<br>Range<br>4-293

For peer review only

Table 2. Analysis of unscheduled and total medical contacts

A. For all children in the intent to treat population.

	Time Period	Treatment Arm* Intervention (%)	Treatment Arm* Control (%)	Odds-Ratio+	95% Confidence Interval	Treatment Arm* Intervention (Mean)	Treatment Arm* Control (Mean)	Incidence Ratio+	95% Confidence Interval
Unscheduled Contacts	Sep	45.2	43.7	1.09	0.96 to 1.25	0.81	0.81	1.02	0.94 to 1.12
	Sep-Dec	80.1	79.1	1.10	0.96 to 1.26	3.19	3.32	0.98	0.93 to 1.04
	Sep-Aug	93.1	93.3	0.97	0.82 to 1.15	9.08	9.37	0.97	0.95 to 1.04
Total Contacts	Sep	57.8	58.4	0.99	0.80 to 1.22	1.05	1.10	0.97	0.87 to 1.07
	Sep-Dec	89.3	88.4	1.06	0.89 to 1.27	4.31	4.43	0.95	0.90 to 1.02
	Sep-Aug	96.6	96.4	0.89	0.71 to 1.12	11.52	12.08	0.95	0.91 to 0.99

* the proportions and means are simple summary statistics

+ the odds-ratios and incidence ratios with the corresponding confidence intervals are from a formal statistical analysis allowing for covariates and the effect of clustering.

B. For children receiving preventer medication in the intent to treat population.

	Time Period	Treatment Arm* Intervention (%)	Treatment Arm* Control (%)	Odds-Ratio+	95% Confidence Interval	Treatment Arm* Intervention (Mean)	Treatment Arm* Control (Mean)	Incidence Ratio+	95% Confidence Interval
Unscheduled Contacts	Sep	46.3	45.4	1.07	0.94 to 1.23	0.83	0.84	1.01	0.92 to 1.10
	Sep-Dec	81.3	81.4	1.04	0.90 to 1.21	3.27	3.44	0.97	0.92 to 1.03
	Sep-Aug	93.9	94.6	0.84	0.69 to 1.02	9.31	9.71	0.98	0.92 to 1.14
Total Contacts	Sep	59.1	60.4	0.97	0.79 to 1.21	1.08	1.14	0.96	0.86 to 1.07
	Sep-Dec	90.4	90.5	0.98	0.81 to 1.19	4.43	4.70	0.95	0.89to 1.01
	Sep-Aug	97.1	97.3	0.81	0.64 to 1.01	11.82	12.53	0.96	0.90 to 1.12

* the proportions and means are simple summary statistics

+ the odds-ratios and incidence ratios with the corresponding confidence intervals are from a formal statistical analysis allowing for covariates and the effect of clustering.

For peer review only

Table 3. Analysis of prescription and scheduled contacts for August.

		Treatment Arm*		Odds-Ratio+	95% Confidence Interval	Treatment Arm*		Incidence Ratio+	95% Confidence Interval
		Intervention (%)	Control (%)			Intervention (Mean)	Control (Mean)		
Prescriptions	All Children	16.5	12.6	1.43	1.24 to 1.64	0.17	0.13	1.31	1.17 to 1.48
	Preventer	17.3	13.4	1.41	1 23 to 1.63	0.18	0.14	1.30	1.16 to 1.47
Scheduled Contacts	All Children	14.3	13.9	1.13	0.84 to 1.52	0.17	0.16	1.17	1.06 to 1.29
	Preventer	14.8	14.4	1.14	0.84 to 1.54	0.18	0.17	1.17	1.06 to 1.29

* the proportions and means are simple summary statistics

+ the odds-ratios and incidence ratios with the corresponding confidence intervals are from a formal statistical analysis allowing for covariates and the effect of clustering.

For peer review only

Figure 1. CONSORT Diagram

Enrollment

GP practices approached (n=456 practices)

Randomized (n=141 practices; n=12179 individuals)

Experimental

Allocated to intervention (n=70 practices; n=5917 individuals)
- Received allocated intervention (n=4436 individuals)
- Did not receive allocated intervention as per protocol (n=1481 individuals)
  - GP exclusions (n=786 individuals)
  - Letter sent late or not at all (n=6 practices; n=695 individuals)

Control

Allocated to intervention (n=71 practices; 6262 individuals)
- Received allocated intervention (n=6262)
- Did not receive allocated intervention (n=0)

Allocation

Follow-Up

Experimental — Discontinued intervention:
- Withdrawal <2014-09-30 (n=13 practices; n=1506 individuals)

Control — Discontinued intervention:
- Withdrawal <2014-09-30 (n=18 practices; n=1824 individuals)

Analysis

Experimental — Analysed:

		ITT	PP
Prescription uptake & scheduled medical contacts	**Aug 13**	N_P = 68<br>P_I = 5305<br>P_O = 612*ǂ	N_P = 62<br>P_I = 4203<br>P_O = 1714*ǂ
	**Aug 13-Jul 14**	N_P = 58<br>P_I = 4541<br>P_O = 764	N_P = 54<br>P_I = 3641<br>P_O = 562
	**Aug 14**	N_P = 58<br>P_I = 4541<br>P_O = 0	N_P = 54<br>P_I = 3641<br>P_O = 0
All medical contacts	**Sept 13**	N_P = 68<br>P_I = 5305<br>P_O = 612*ǂ	N_P = 62<br>P_I = 4203<br>P_O = 1714*ǂ
	**Sept-Dec 13**	N_P = 65<br>P_I = 5097<br>P_O = 208	N_P = 59<br>P_I = 4021<br>P_O = 182
	**Sept 13-Aug 14**	N_P = 58<br>P_I = 4541<br>P_O = 556	N_P = 54<br>P_I = 3641<br>P_O = 380
	**Sept 14**	N_P = 57<br>P_I = 4411<br>P_O = 130	N_P = 54<br>P_I = 3641<br>P_O = 0

Control — Analysed:

		ITT	PP
Prescription uptake & scheduled medical contacts	**Aug 13**	N_P = 69<br>P_I = 5586<br>P_O = 676**ǂ	N_P = 69<br>P_I = 5586<br>P_O = 676**ǂ
	**Aug 13-Jul 14**	N_P = 54<br>P_I = 4549<br>P_O = 1037	N_P = 54<br>P_I = 4549<br>P_O = 1037
	**Aug 14**	N_P = 54<br>P_I = 4549<br>P_O = 0	N_P = 54<br>P_I = 4549<br>P_O = 0
All medical contacts	**Sept 13**	N_P = 69<br>P_I = 5586<br>P_O = 676**ǂ	N_P = 69<br>P_I = 5586<br>P_O = 676**ǂ
	**Sept-Dec 13**	N_P = 67<br>P_I = 5384<br>P_O = 202	N_P = 67<br>P_I = 5384<br>P_O = 202
	**Sept 13-Aug 14**	N_P = 54<br>P_I = 4549<br>P_O = 835	N_P = 54<br>P_I = 4549<br>P_O = 835
	**Sept 14**	N_P = 53<br>P_I = 4438<br>P_O = 111	N_P = 53<br>P_I = 4438<br>P_O = 111

N_P = Number of practices

P_I = Patients in analysis

P_O = New patients out of analysis (withdrawals), when compared to previous period

* = In comparison to those in the experimental arm (n=5917)

** = In comparison to those in the control arm (n=6262)

ǂ = These figures include withdrawals & patients aged 4.

For peer review only - http://bmjopen.bmj.com/site/about/guidelines.xhtml

For peer review only

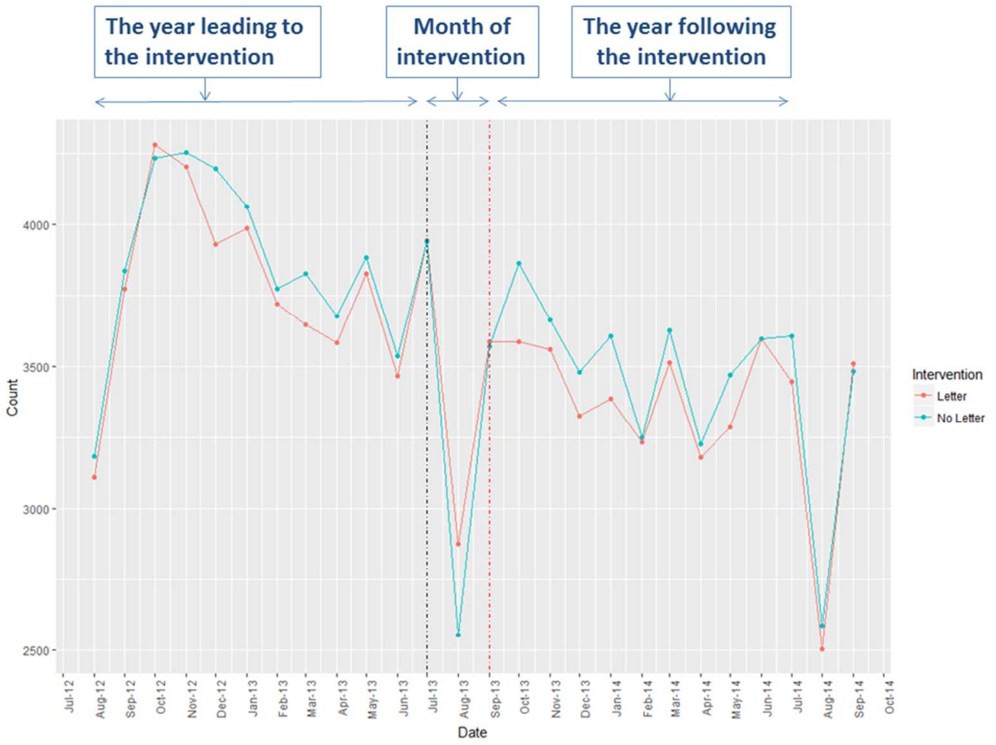

Figure 2. Unscheduled medical contacts and prescriptions over time
a. Unscheduled medical contacts

254x190mm (96 x 96 DPI)

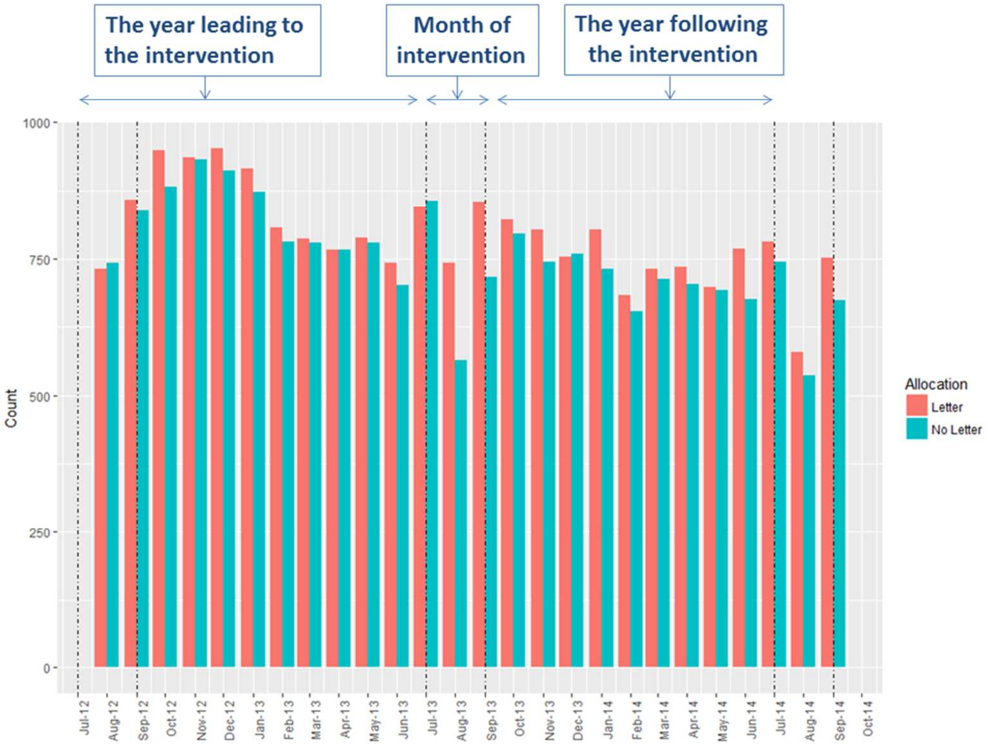

b. Prescriptions for preventer medication

254x190mm (96 x 96 DPI)

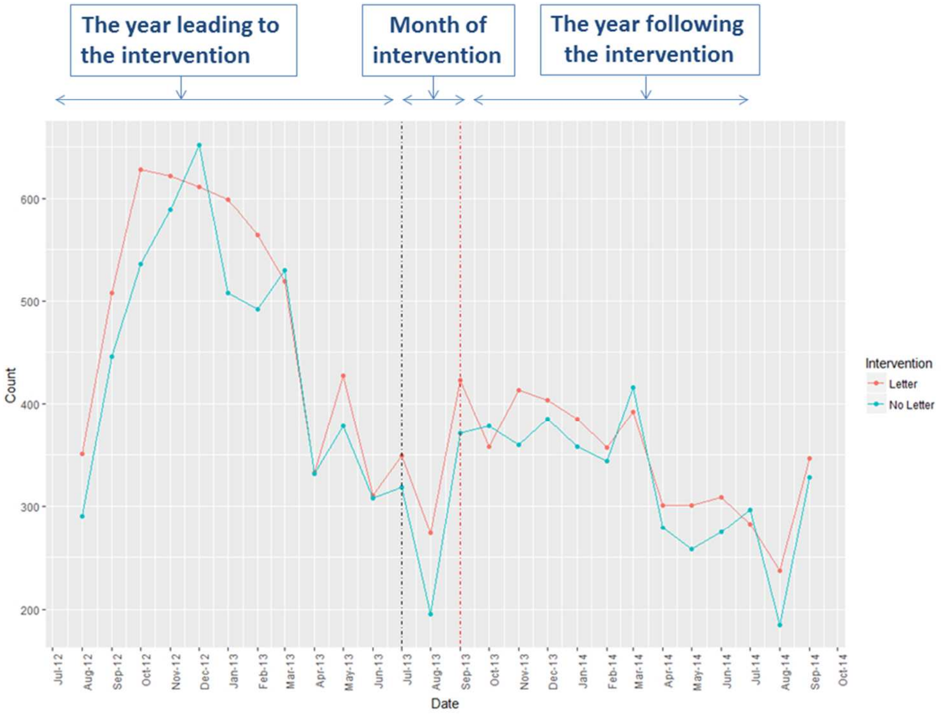

Figure 3. Unscheduled respiratory medical contacts

254x190mm (96 x 96 DPI)

1 APPENDICES

1.1 Appendix 1 . Trial Intervention

GP letterhead

< Address line 1>

< Address line 2>

< Address line 3>

< Address line 4>

<Insert Date>

Dear Parent

Please read this important letter regarding your child's asthma

It is really important that your child continues to take their asthma medication during the summer holidays. Returning to school is a time when asthma can get worse and make children and young people with asthma poorly. This may be due to contact with infections at the start of the new school year.

To reduce the chances of getting poorly when they return to school, your child should continue to take their asthma medication as prescribed by their GP or practice nurse. If your child has stopped taking their medication over the summer holidays it is important to start it again as soon as possible. If they are short of medication, or you are not sure of the proper dose, please get in touch with the practice.

Yours sincerely

<Name of Doctor>

For peer review only - http://bmjopen.bmj.com/site/about/guidelines.xhtml

1.2 Appendix 2. Changes to Protocol

Changes to Protocol		REC approval date	Approved by
Protocol Version 2 (14.05.15): This version included an additional secondary outcome to include data up to September 2014, to see if the effect from September 2013 is maintained when there is no study intervention thus extending the follow up period by one month (see section 2.1.5).	Agreed as a 2-month non-cost contact variation by HTA 02/02/2015	25th May 2014	NRES Committee Yorkshire & Humber – South Yorkshire

For peer review only - http://bmjopen.bmj.com/site/about/guidelines.xhtml

1.3 Appendix 3 - Practice Withdrawal and Adherence to Protocol

Table 4 provides the number of practices and the number of individuals aged 5-16 (the primary analysis population) included for each time period.

Table 4: Number of practices and individuals included within each time period

	**Letter**		**No letter**	
	**Practices**	**Individuals 5-16**	**Practices**	**Individuals 5-16**
Prescription uptake and scheduled medical contacts				
August 2013	68	5305	69	5586
August 2013-July 2014	58	4541	54	4549
August 2014	58	4541	54	4549
All medical contacts				
September 2013 (Primary study period)	68	5305	69	5586
September to December 2013 (extended study period)	65	5097	67	5384
September 2013-August 2014 (twelve month study period)	58	4541	54	4549
September 2014 (Echo sub-study)	57	4411	53	4438

For peer review only

For peer review only - http://bmjopen.bmj.com/site/about/guidelines.xhtml

1.4 Adherence to protocol

Of the 70 intervention practices, 2 did not send letters to any of the patients identified and 4 sent the intervention out late on the 6th, 8th, 12th and 23th of August. In addition, GPs were given discretion to withhold the letter from any children they believed were unsuitable candidates; among the remaining 64 practices (5222 individuals), letters were not sent to 786 children. These individuals were included in the primary ITT analyses but excluded from Per Protocol analyses.

For peer review only

1.5 Appendix 4 - Analysis of Respiratory Contacts

1.5.1 Analysis of unscheduled respiratory related contacts

The unscheduled medical respiratory related contacts are given in Figure 3. The baseline data for the 12 months leading up to the intervention would suggest that the practices randomised the intervention arm have more unscheduled respiratory contacts than the control arm. This would feed through into the summary statistics given in Table 5 which show more contacts in the intervention arm. When compared to Table 4 one can see that around 5% of medical contacts are respiratory related in the analysis.

The adjusted analyses in Table 5 has in the model the corresponding baseline term. The analyses infer an increase in respiratory related medical contacts. The definition of a respiratory related contact for the analysis, however, included that a prescription for an asthma medication was given in the medical contact. The intervention has increased asthma prescriptions and so it is likely that a proportion of these contacts are associated with the increase in prescriptions.

For peer review only

For peer review only - http://bmjopen.bmj.com/site/about/guidelines.xhtml

Table 5. Analysis of unscheduled respiratory related medical contacts

	Time Period	Treatment Arm* Intervention (%)	Treatment Arm* Control (%)	Odds-Ratio+	95% Confidence Interval	Treatment Arm* Intervention (Mean)	Treatment Arm* Control (Mean)	Incidence Ratio+	95% Confidence Interval
All Children	Sep	5.3	4.2	1.30	1.03 to 1.66	0.06	0.05	1.30	1.02 to 1.66
	Sep-Dec	18.4	16.7	1.13	0.95 to 1.33	0.23	0.21	1.10	0.95 to 1.27
	Sep-Aug	38.0	35.3	1.05	0.87 to 1.33	0.57	0.53	1.04	0.90 to 1.20
Children Receiving Preventers	Sep	5.5	4.4	1.30	1.02 to 1.66	0.06	0.05	1.30	1.01 to 1.66
	Sep-Dec	19.2	17.4	1.13	0.96 to 1.34	0.24	0.22	1.10	0.95 to 1.28
	Sep-Aug	39.3	36.9	1.06	0.87 to 1.30	0.59	0.55	1.06	0.92 to 1.23

* the proportions and means are simple summary statistics

+ the odds-ratios and incidence ratios with the corresponding confidence intervals are from a formal statistical analysis allowing for covariates.

For peer review only

For peer review only - http://bmjopen.bmj.com/site/about/guidelines.xhtml

PLEASANT Statistical Analysis Plan – version 1.0

Project Title: The PLEASANT Study

(Preventing and Lessening Exacerbations of Asthma in School-age children Associated with a New Term)

A cluster randomised controlled trial investigating the effect of a postal intervention in reducing unscheduled medical contacts in school age children following returning to school.

Statistical Analysis Plan

Version 1.1.0

Authored by ________________________________ ___/___/_____

Benjamin Sully Date

PLEASANT trial statistician

Medical Statistics Group, DTS, University of Sheffield

Approved by ________________________________ ___/___/_____

Neil Shephard Date

PLEASANT trial statistician

CTRU, DTS, University of Sheffield

________________________________ ___/___/_____

Mike Bradburn Date

PLEASANT trial Senior statistician

CTRU, DTS, University of Sheffield

For peer review only - http://bmjopen.bmj.com/site/about/guidelines.xhtml

PLEASANT Statistical Analysis Plan – version 1.0

_______________________________ ___/___/_____

Professor Steven A. Julious Date

PLEASANT chief investigator

Medical Statistics Group, DTS, University of Sheffield

_______________________________ ___/___/_____

Martyn Lewis Date

Reader in Biostatistics

PLEASANT Trial Steering Committee Statistician

Primary Care Sciences, Keele University

For peer review only

For peer review only - http://bmjopen.bmj.com/site/about/guidelines.xhtml

PLEASANT Statistical Analysis Plan – version 1.0

List of abbreviations used

AE	Adverse Event
AFT	Accelerated Failure Time
CI	Confidence Interval
CPRD	Clinical Practice Research Datalink
CTRU	Clinical Trials Research Unit
GP	General Practitioner
HTA	Health Technology Assessment
ICC	Intra-Class Correlation
ITT	Intent-To-Treat
MPR	Medicine Possessions Ratio
NHS	National Health Service
SOP	Standard Operating Procedure
TMG	Trial Management Group
TSC	Trial Steering Committee
UoS	University of Sheffield

For peer review only

For peer review only - http://bmjopen.bmj.com/site/about/guidelines.xhtml

PLEASANT Statistical Analysis Plan – version 1.0

1 Introduction, study design and key trial objectives

1.1 Study outline

The PLEASANT study is a parallel group, cluster randomised controlled trial that will compare a postal intervention to standard care in children aged 4-16 with previous diagnoses of asthma; 70 General Practices (GPs) will be randomised to each arm, and patients from these GPs will receive the appropriate intervention.

This statistical analysis plan is written in conjunction with the International Conference on Harmonisation topic E9 (Statistical principles for clinical trials), applicable standard operating procedures from the Sheffield Clinical Trials Research Unit (CTRU) and trial documents referenced in section 4.

This trial is funded by the National Health Service (NHS) Health Technology Assessment (HTA).

1.2 Outcome measures

1.2.1 Primary outcome measure

- The proportion of patients aged between 5-16 who have an unscheduled medical contact in September

1.2.2 Secondary outcome measures

- The proportion of patients who have an unscheduled medical contact in the period September – December
- The total number of medical contacts (scheduled and unscheduled) per patient in September and in the period September – December
- The time to first unscheduled medical contact in September and in the period September – December

For peer review only - http://bmjopen.bmj.com/site/about/guidelines.xhtml

PLEASANT Statistical Analysis Plan – version 1.0

- The proportion of patients who have a medical contact (either scheduled or unscheduled) in September and in the period September – December
- The total number of medical contacts (scheduled and unscheduled) per patient in September and in the period September – December
- The time to first medical contact in September and in the period September – December
- The proportion of patients who have an unscheduled medical contact in September and in the period September – December associated with a respiratory diagnosis
- The number of unscheduled medical contacts per patient in September and in the period September – December associated with a respiratory diagnosis
- The time to first unscheduled medical contact associated with a respiratory diagnosis in September and in the period September – December
- The number of prescriptions per patient in the month of August
- The number of prescriptions in the 12 months following the intervention
- The proportion of patients who have a scheduled medical contact (for example asthma review) in August
- The proportion of patients who have a scheduled medical contact (for example Asthma review) in the 12 months following the intervention.

All above analyses will be undertaken and reported twice: once on patients under the age of 5 and once on patients aged between 5 and 16. This is because asthma is difficult to diagnose in children below this age[1-2].

For peer review only - http://bmjopen.bmj.com/site/about/guidelines.xhtml

1.2.3 Sample size

From previous research in the CPRD practice population 30% of school age asthmatic children had at least one unscheduled medical contact within the month of September13. We postulate that the intervention may reduce the number of children who have unscheduled medical contacts from 30% to 25% (i.e. an absolute reduction of 5%). We would have an effect size of 5%. The average practice size in the CPRD is 8,294. We thus anticipate circa 100 school age asthmatic patients per practice (based on 12% of a practice being school age children and 11% of school age children having asthma). Hence, to detect a difference of 5% with 90% power and two sided significance level of 5%, with an intra-class correlation (ICC) of 0.03 to account for clustering we require 70 practices per arm. The sample size of 140 practices would equate to approximately 14,000 school age asthmatic patients.

Ukoumunne et al[3] give estimates of ICCs for patients with respiratory symptoms in General Practice. Based on the work of Ukoumunne et al an ICC of 0.03 is a conservative estimate. The power of the study for ICCs of 0.01, 0.02, 0.03, 0.04 and 0.05 is respectively 99.4, 96.0, 90.0, 83.1 and 76.2%

As a further sensitivity analysis we investigated the effect of practices not sending out the letter as planned. Suppose 10 practices failed to send out the letter, these would still be included in the primary analysis under the intent to treat principle. However, the effect that could be observed would be reduced to 4.3%. Under the sample assumptions (ICC=0.03 etc) the power for the same sample size is reduced to 79.3%. This is a little under 80% but it does demonstrate that the study is reasonably robust to at least one deviation in the planned design.

For peer review only - http://bmjopen.bmj.com/site/about/guidelines.xhtml

1.2.4 Randomisation

The study is a cluster randomised trial; 70 general practices (GPs) undertaking the intervention and 70 control practices of "usual care". The randomisation will be stratified by size of GP to ensure that there is an equal sample size – in terms of number of school age asthmatic children – in each arm of the trial. Practices will be randomised to one of the two arms after they have agreed to participate. The randomisation will be carried out by the University of Sheffield (UoS) Clinical Trials Research Unit (CTRU) using a randomisation plan developed prior to the beginning of the trial.

1.2.5 Interim analyses and study committees

Two committees will be established to govern the conduct of the study:

1. Trial Management Group (TMG)
2. Trial Steering Committee (TSC)

All committees are governed by Sheffield CTRU standard operating procedures. The TMG consists of the Principal Investigator, co-investigators and key staff within the CTRU. The role of the TMG is to implement all parts of the trial.

The TSC consists of the Principal Investigator, key staff within the CTRU (as non-voting members), an independent chair and two independent members (including a statistician) and 2 lay members. The roles of the TSC are to provide supervision of the protocol and statistical analysis plan, provide advice on and monitor progress of the trial.

No formal interim analyses are required in the study.

For peer review only - http://bmjopen.bmj.com/site/about/guidelines.xhtml

2 Data sources, data evaluability and analysis populations

2.1 Data sources

The data for this study will be collected and managed by the Clinical Practice Research Datalink (CPRD), a computerised database of anonymised longitudinal medical records from primary care. The CPRD are able to capture all medical contacts along with the reason for the contact.

The PLEASANT study team at CTRU will request and collect the appropriate data from CPRD at three time points:

1. Baseline
2. 1 month post intervention
3. 12 months post intervention

The data requested from CPRD will include, for each patient:

- Age
- Gender
- General Practice identifier
- The date of each appointment
- The type of medical contact for each appointment
- The diagnosis given for each appointment
- Any prescriptions given as a result of an appointment.

2.2 Data evaluability

Upon receiving the data from CPRD, CTRU will handle and prepare the data for statistical analysis. This includes forwarding data pertaining to the nature

For peer review only - http://bmjopen.bmj.com/site/about/guidelines.xhtml

of each appointment to an adjudication panel for their review, who will in turn define appointments as being either scheduled or unscheduled. The CTRU will also merge treatment allocation data with CPRD data and calculate the number of appointments for each patient.

Detailed data management and data quality issues will be set out in a data management plan. Data will be retained in accordance with the Data Protection Act 1998 and CTRU data management Standard Operating Procedures (SOPs).

All source documents and data will be retained for a period of at least 5 years following the end of the trial.

2.3 Analysis populations

The analysis populations will be as follows:

Intent To Treat (ITT)	All randomised patients identified through the extraction identified by the CPRD.
Per protocol (PP)	The subset of the ITT who belong to a practice which complies to the protocol, meet the inclusion/exclusion criteria and whom the GPs did not exclude from receiving the intervention.

All analyses will be performed on both study populations.

There are three study periods to be analysed. The primary analysis will be undertaken on the primary study period; secondary analyses will use all three stages.

For peer review only - http://bmjopen.bmj.com/site/about/guidelines.xhtml

Primary study period 1st September 2013 – 30th September 2013

Extended study period 1st September 2013 – 31st December 2013

Follow-up period 1st September 2013 – 31st August 2014

3 Outline of analyses

3.1 General considerations

Summaries of continuous variables will comprise the sample size used and either:

i. mean, standard deviation, minimum and maximum, or

ii. median, inter-quartile range, minimum and maximum

as appropriate for the distributional form of the data. Summaries of categorical variables will comprise the sample size used, and the number and percentage of observations in each category.

3.1.1 Levels of statistical significance and adjustment for multiplicity

The PLEASANT study was designed and planned using a 2-sided significance level of 5%. All analyses will be undertaken using this level of significance. As there is only one primary outcome and no interim analysis, adjustment for multiplicity is unnecessary. However adjustments will be made for the multitude of secondary outcomes. Conservative Bonferroni corrections will be

For peer review only

made to the raw p-values and where possible k-fold cross-validation will be performed by using a leave-one-out approach.

3.1.2 Rules for derived variables

The number of appointments for each patient will be calculated after the panel has determined whether appointments were scheduled or unscheduled. The numbers of each will then be summed (for both the primary and extended study periods). There are instances where no medical code has been used to record the type of medical contact and instead free-form text has been entered. Such entries will always be unscheduled (because scheduled contacts are recorded so that GPs are remunerated) but it is impossible to determine the nature of the contact and therefore whether it is respiratory related or not. The number of each contact type, in terms of "relevant" /" irrelevant", "scheduled" /" unscheduled" /" unknown" and "respiratory related" /" not respiratory related" /" indeterminable" will be reported.

The proportion of patients with unscheduled medical contacts in September 2013 will be analysed using a derived variable. Any patients who have had one or more unscheduled medical contacts in this period will be coded as '1', while those who have had zero unscheduled medical contacts in this period will be coded as '0'. This binary variable will then be used as the dependent variable in the analysis. This will be done for all outcomes involving a proportion of patients.

For peer review only - http://bmjopen.bmj.com/site/about/guidelines.xhtml

3.2 Disposition

The following summary will be presented for all practices and patients:

- Centre disposition: the number and percentage of practices included in each analysis population with reason for exclusion
- Patient disposition: the number and percentage of patients included in each analysis population with reason for exclusion

The following summary will be presented for the ITT:

- Data completeness: the number of patients with complete data for key parameters by treatment group
- Data completeness by practice: the number of patients with complete data for key parameters by practice.

3.3 Demographics and baseline characteristics

The following summaries will be presented:

- Demographics: age; gender; practice; number of asthma admissions in September 2012, the period 1st September – 31st December 2012 and the period 1st September 2012 – 31st August 2013 (scheduled, unscheduled and both combined); time to first medical contact in September and the period 1st September – 31st December 2012 (scheduled, unscheduled and both combined).

The outputs to be presented are shown in Tables 4 and 5.

For peer review only - http://bmjopen.bmj.com/site/about/guidelines.xhtml

3.4 Efficacy

3.4.1 Primary outcome

The primary outcome will be analysed by intent to treat among patients aged 5-16 as of 1st September 2012. The primary endpoint (the proportion of patients who have an unscheduled medical contact in September) will be analysed by logistic regression in which the fixed covariates will include the individual’ s age, gender, number of contacts the previous September, and trial arm; GP will be included as a random effect to account for the effect of clustering by practice.

The following outputs will be presented for the ITT:

- The number of unscheduled medical contacts in September 2013
- The proportion of patients having unscheduled medical contacts in September 2013
- The results of the logistic regression modelling for the primary outcome, summarising the effect of all covariates fitted in the model.

All outputs to be presented are shown in Table 1.

3.4.2 Secondary outcomes

3.4.2.1 Proportion of patients with medical contacts

For analysis of secondary outcomes involving proportions of patients in both the extended period and the follow-up study, the same approach will be used as for the primary outcome. Similar covariates will be included in the analysis, ensuring that the baseline variable matches the outcome variable. For

For peer review only - http://bmjopen.bmj.com/site/about/guidelines.xhtml

example, when analysing the proportion of patients who have an unscheduled medical contact in the period September – December associated with a respiratory diagnosis, the baseline covariate will be the number of contacts in the previous September – December associated with a respiratory diagnosis.

Outputs to be presented are shown in Table 1.

3.4.2.2 Number of patients with medical contacts

For outcomes involving numbers of medical appointments or prescriptions the intervention will be analysed in an analogous approach to those involving proportions. A random effects negative binomial model will be fitted, including the same covariates as above.

Outputs to be presented are shown in Table 2.

3.4.2.3 Time to first medical contact

Analyses involving the time to first medical contact will all be analysed using a random effects ("shared frailty") regression model including the same covariates as described previously. Due to the expected high prevalence of ties (i.e. the same time to first contact) the Efron method for handling ties will be used.

Outputs to be presented are shown in Table 3.

For peer review only - http://bmjopen.bmj.com/site/about/guidelines.xhtml

3.5 Testing assumptions of statistical analyses

The primary outcome will be analysed using a random effects logistic regression model. This modelling technique is very robust and makes very few assumptions. The same applies for the secondary analyses involving proportional dependent variables. The Hosmer-Lemeshow test will be used to test the goodness of fit for these models.

The secondary analyses involving number of events will be analysed using random effects negative binomial regression. Similarly to above, this method is very flexible and does not rely on assumptions.

Analyses involving time-to-event data will be analysed using random effects "shared frailty" Cox regression. The key assumption underlying this analysis method is that the hazard in one group (or one level for a continuous covariate) is a constant multiple of that in another group (level). This will be tested by fitting an interaction term between time and treatment arm: if the hazard ratio is constant, this term will be non-significant. If the hazard ratio is found to be non-constant over time the outcome will instead be analysed using Accelerated Failure Time (AFT), with goodness of fit assessed by Q-Q plots[4]. If the assumptions underlying this method are not met, residual mean survival methods will be used[5].

3.6 Compliance

Compliance will be based on whether or not *practices* comply with the intervention i.e. whether they send out the letter. To check for differences between complying and non-complying practices the demographics for each

For peer review only

population will be reported (Tables 4 and 5). Tables displaying outcome data (Tables 1, 2 and 3) will also be reported split by compliance.

3.7 Economic analyses

Economic analyses will be included in a separate document.

3.8 Analysis of non-adherence

In order to identify patients who are non-adherent to regular asthma treatments the medicine possessions ratio (MPR) for each participant will be calculated as the following:

$$MPR = 100 \times \frac{numberofdaysofmedicineprescribed \in last\,12\,months}{365}.$$

This will be calculated at baseline (the year prior to the intervention) and at follow-up (the year following the intervention). Patients with an MPR of under 80% will be classed as 'non-adherent' to medicine.

The MPR will be calculated for preventative medications only, using prescription information. The analysis will be undertaken only on patients who have a single medication which remains the same over both baseline and follow-up; patients prescribed more than one preventative medication or who switch medications between periods will be excluded from this analysis.

Informal analysis will take place to ensure that the MPRs are independent across treatment arms at baseline and also independent across time points in the control group. This will comprise histograms of the MPR and summary statistics.

For peer review only - http://bmjopen.bmj.com/site/about/guidelines.xhtml

The main analysis of MPR will test whether the intervention changes MPR. This will be done in two ways:

1. A test for change in proportion of patients classed as non-adherent before and after the intervention in control and intervention arms.

2. Testing the difference in change in MPR before and after the intervention between the control and intervention arms.

A separate subgroup analysis will investigate whether patients who are classed as non-adherent at baseline respond differently to the intervention to those who are classed as adherent.

1. Paired t-test, intervention group only, comparing difference between baseline and follow-up for adherent vs non-adherent.

4 References

4.1 Trial Documents

Trial Protocol (version 1.6, 25/09/2012)

4.2 Other References

1. British Thoracic Society / Scottish Intercollegiate Guideline Network. British Guideline on the Management of Asthma: A national clinical guideline. Jan 2012.

2. Bush A. Diagnosis of asthma in children under five. Primary Care Respiratory Journal. 2007;16(1):7-15

For peer review only

For peer review only - http://bmjopen.bmj.com/site/about/guidelines.xhtml

3. Ukoumunne OC, Gulliford MC, Chinn S, Sterne JAC and Burney PJG. Methods for evaluating area-wide and organisation-based interventions in health and health care: a systematic review. Health Technology Assessment 1999; Vol. 3: No. 5.

4. Bradburn MJ, Clark TG, Love SB and DG Altman DG. Survival Analysis Part III: Multivariate data analysis – choosing a model and assessing its adequacy and fit. British Journal of Cancer (2003) 89, 605–611.

5. Royston P and Parmar MKB. The use of restricted mean survival time to analyse randomized clinical trials data when the proportional hazards assumption is in doubt. Statistics in Medicine 2011; 30: 2409-2421.

For peer review only

Table 1: CONSORT 2010 checklist of information to include when reporting a cluster randomised trial

Section/Topic	Item No	Standard Checklist item	Extension for cluster designs	Page No *
**Title and abstract**				
	1a	Identification as a randomised trial in the title	Identification as a cluster randomised trial in the title	1
	1b	Structured summary of trial design, methods, results, and conclusions (for specific guidance see CONSORT for abstracts)[1,2]	See table 2	2
**Introduction**				
**Background and objectives**	2a	Scientific background and explanation of rationale	Rationale for using a cluster design	4
	2b	Specific objectives or hypotheses	Whether objectives pertain to the the cluster level, the individual participant level or both	4
**Methods**				
**Trial design**	3a	Description of trial design (such as parallel, factorial) including allocation ratio	Definition of cluster and description of how the design features apply to the clusters	6
	3b	Important changes to methods after trial commencement (such as eligibility criteria), with reasons		N/A
**Participants**	4a	Eligibility criteria for participants	Eligibility criteria for clusters	6
	4b	Settings and locations where the data were collected		8
**Interventions**	5	The interventions for each group with sufficient details to allow replication, including how and when they were actually administered	Whether interventions pertain to the cluster level, the individual participant level or both	6
**Outcomes**	6a	Completely defined pre-specified primary and secondary outcome measures, including how and	Whether outcome measures pertain to the cluster level, the individual participant level or both	5-6

		when they were assessed		
	6b	Any changes to trial outcomes after the trial commenced, with reasons		**15**
**Sample size**	7a	How sample size was determined	Method of calculation, number of clusters(s) (and whether equal or unequal cluster sizes are assumed), cluster size, a coefficient of intracluster correlation (ICC or *k*), and an indication of its uncertainty	9
	7b	When applicable, explanation of any interim analyses and stopping guidelines		**N/A**
**Randomisation:**				
**Sequence generation**	8a	Method used to generate the random allocation sequence		**7-8**
	8b	Type of randomisation; details of any restriction (such as blocking and block size)	Details of stratification or matching if used	7-8
**Allocation concealment mechanism**	9	Mechanism used to implement the random allocation sequence (such as sequentially numbered containers), describing any steps taken to conceal the sequence until interventions were assigned	Specification that allocation was based on clusters rather than individuals and whether allocation concealment (if any) was at the cluster level, the individual participant level or both	7-8
**Implementation**	10	Who generated the random allocation sequence, who enrolled participants, and who assigned participants to interventions	Replace by 10a, 10b and 10c	7-8
	10a		Who generated the random allocation sequence, who enrolled clusters, and who assigned clusters to interventions	7-8
	10b		Mechanism by which individual participants were included in clusters for the purposes of the trial (such as complete	8

For peer review only

			enumeration, random sampling)	
	10c		From whom consent was sought (representatives of the cluster, or individual cluster members, or both), and whether consent was sought before or after randomisation	11
**Blinding**	11a	If done, who was blinded after assignment to interventions (for example, participants, care providers, those assessing outcomes) and how		**8-9**
	11b	If relevant, description of the similarity of interventions		**N/A**
**Statistical methods**	12a	Statistical methods used to compare groups for primary and secondary outcomes	How clustering was taken into account	9-11
	12b	Methods for additional analyses, such as subgroup analyses and adjusted analyses		**9-11**
**Results**				
**Participant flow (a diagram is strongly recommended)**	13a	For each group, the numbers of participants who were randomly assigned, received intended treatment, and were analysed for the primary outcome	For each group, the numbers of clusters that were randomly assigned, received intended treatment, and were analysed for the primary outcome	11
	13b	For each group, losses and exclusions after randomisation, together with reasons	For each group, losses and exclusions for both clusters and individual cluster members	11
**Recruitment**	14a	Dates defining the periods of recruitment and follow-up		**11**
	14b	Why the trial ended or was stopped		**N/A**
**Baseline data**	15	A table showing baseline demographic and clinical	Baseline characteristics for the individual and cluster levels as	26

For peer review only

		characteristics for each group	applicable for each group	
**Numbers analysed**	16	For each group, number of participants (denominator) included in each analysis and whether the analysis was by original assigned groups	For each group, number of clusters included in each analysis	26
**Outcomes and estimation**	17a	For each primary and secondary outcome, results for each group, and the estimated effect size and its precision (such as 95% confidence interval)	Results at the individual or cluster level as applicable and a coefficient of intracluster correlation (ICC or k) for each primary outcome	26
	17b	For binary outcomes, presentation of both absolute and relative effect sizes is recommended		**27-28**
**Ancillary analyses**	18	Results of any other analyses performed, including subgroup analyses and adjusted analyses, distinguishing pre-specified from exploratory		**27-28**
**Harms**	19	All important harms or unintended effects in each group (for specific guidance see CONSORT for harms[3])		**N/A**
**Discussion**				
**Limitations**	20	Trial limitations, addressing sources of potential bias, imprecision, and, if relevant, multiplicity of analyses		**15-18**
**Generalisability**	21	Generalisability (external validity, applicability) of the trial findings	Generalisability to clusters and/or individual participants (as relevant)	15-18
**Interpretation**	22	Interpretation consistent with results, balancing benefits and harms, and considering other relevant evidence		**15-18**
**Other information**				
**Registration**	23	Registration number and		**3 and 22**

For peer review only

		name of trial registry	
**Protocol**	24	Where the full trial protocol can be accessed, if available	**20, Ref 15**
**Funding**	25	Sources of funding and other support (such as supply of drugs), role of funders	**3 and 23**

** Note: page numbers optional depending on journal requirements*

For peer review only

Table 2. Items to include when reporting a randomized trial in a journal or conference abstract

Item	Description	Reported on line number
Title	Identification of the study as randomized	Y
Authors *	Contact details for the corresponding author	Y
Trial design	Description of the trial design (e.g. parallel, cluster, non-inferiority)	Y
Methods		
Participants	Eligibility criteria for participants and the settings where the data were collected	Y
Interventions	Interventions intended for each group	Y
Objective	Specific objective or hypothesis	Y
Outcome	Clearly defined primary outcome for this report	Y
Randomization	How participants were allocated to interventions	Y
Blinding (masking)	Whether or not participants, care givers, and those assessing the outcomes were blinded to group assignment	N
Results		
Numbers randomized	Number of participants randomized to each group	N/A (Number of clusters provided)
Recruitment	Trial status	N
Numbers analysed	Number of participants analysed in each group	N/A (Number of clusters provided)
Outcome	For the primary outcome, a result for each group and the estimated effect size and its precision	Y
Harms	Important adverse events or side effects	N/A
Conclusions	General interpretation of the results	Y
Trial registration	Registration number and name of trial register	Y
Funding	Source of funding	Y

**this item is specific to conference abstracts*

For peer review only - http://bmjopen.bmj.com/site/about/guidelines.xhtml

An Open-Label Cluster Randomised Controlled Trial and Economic Evaluation of a Brief Letter from a GP on Unscheduled Medical Contacts Associated with the Start of the School Year – The PLEASANT Trial

Journal:	*BMJ Open*
Manuscript ID	bmjopen-2017-017367.R2
Article Type:	Research
Date Submitted by the Author:	15-Dec-2017
Complete List of Authors:	Julious, Steven; The University of Sheffield, Medical Statistics Group, ScHARR,<br>Horspool, Michelle; University of Sheffield, Medical Statistics Group, School of Health and Related Research (ScHARR),<br>Davis, Sarah; University of Sheffield, Health Economics and Decision Sciences, School of Health and Related Research (ScHARR)<br>Franklin, Matthew; University of Sheffield, Health Economics and Decision Sciences, School of Health and Related Research (ScHARR)<br>Smithson, Henry; University of Cork, Department of General Practice<br>Norman, Paul; University of Sheffield, Department of Psychology<br>Simpson, Rebecca; University of Sheffield , Medical Statistics Group, School of Health and Related Research (ScHARR)<br>Elphick, Heather; Sheffield Children's Hospital, Respiratory Department<br>Bortolami, Oscar; University of Sheffield, Medical Statistics Group, School of Health and Related Research (ScHARR)<br>Cooper, Cindy; University of Sheffield , Clinical Trials Research Unit, School of Health and Related Research (ScHARR)
**Primary Subject Heading**:	Respiratory medicine
Secondary Subject Heading:	Paediatrics, Public health, General practice / Family practice, Health economics, Medical management
Keywords:	Asthma < THORACIC MEDICINE, PRIMARY CARE, Cluster Trial, Randomised Controlled Trial, School-aged Children, Adherence

An Open-Label Cluster Randomised Controlled Trial and Economic Evaluation of a Brief Letter from a GP on Unscheduled Medical Contacts Associated with the Start of the School Year – The PLEASANT Trial

Authors: Prof Steven A Julious[a], Dr Michelle J Horspool[a], Sarah Davis[b], Dr Matthew Franklin[b], Prof W Henry Smithson[c], Prof Paul Norman[f], Rebecca M Simpson, Prof Heather Elphick[d], Dr Oscar Bortolami[a], Prof Cindy Cooper[e],

Author Institutions

[a] Medical Statistics Group, School of Health and Related Research (ScHARR), University of Sheffield, Sheffield, United Kingdom

[b] Health Economics and Decision Sciences, School of Health and Related Research (ScHARR), University of Sheffield, Sheffield, United Kingdom

[c] Department of General Practice, University of Cork, Cork, Ireland

[d] Respiratory Department, Sheffield Children's Hospital, Sheffield, United Kingdom

[e] Clinical Trials Research Unit, School of Health and Related Research (ScHARR), University of Sheffield, Sheffield, United Kingdom

[f] Department of Psychology, University of Sheffield, Sheffield, United Kingdom

Corresponding author:

Name: Prof Steven A Julious

Address: Medical Statistics Group, School of Health and Related Research, The University of Sheffield, Regent Court, Regent Street, Sheffield, S1 4DA

Tel: 0114 2220709

Fax: 0114 2220749

Email: s.a.julious@sheffield.ac.uk

For peer review only

ABSTRACT (298/300 words max)

Background: Asthma is seasonal with peaks in exacerbation rates in school-aged children associated with the return to school following the summer vacation. A drop in prescription collection in August is associated with an increase in the number of unscheduled contacts after the school return.

Objective: To assess whether a public health intervention delivered in general practice reduced unscheduled medical contacts in children with asthma.

Design: Cluster-randomised trial with trial-based economic evaluation. Randomisation was at general practice level, stratified by size of practice. The intervention group received a letter from their GP in late July outlining the importance of (re)taking asthma medication before the return to school. The control group was usual care.

Setting: General practices in England and Wales.

Participants: 12179 school-aged children in 142 general practices (70 randomised to intervention).

Main Outcome: Proportion of children aged 5-16 who had an unscheduled contact in September. Secondary endpoints included collection of prescriptions in August and medical contacts over 12 months (September-August). Economic endpoints were quality-adjusted life-years gained and health service costs.

Results: There was no evidence of effect (odds-ratio 1.09; 95% CI 0.96 to 1.25 against treatment) on unscheduled contacts in September. The intervention increased the proportion of children collecting a prescription in August by 4% (odds-ratio 1.43; 95% CI 1.24 to 1.64). The intervention also reduced the total number of medical contacts between September-August by 5% (incidence ratio 0.95; 95% CI 0.91 to 0.99).

The mean reduction in medical contacts informed the health economics analyses. The intervention was estimated to save £36.07 per patient - with a high probability (96.3%) of being cost-saving.

For peer review only

Conclusions: The intervention succeeded in increasing children collecting prescriptions. It did not reduce unscheduled care in September (the primary outcome), but in the year following the intervention, it reduced the total number of medical contacts.

Trial registration number: ISRCTN03000938

Key Words

Asthma, school-age children, primary care, cluster trial, general practice, randomised controlled trial, CPRD, unscheduled care, scheduled care, adherence

Funding details

This project was funded by the NIHR Health Technology Assessment programme

Strengths and limitations of this study

- The evaluation was a highly efficient study design using routine data to evaluate a general practice public health intervention designed with children with asthma and their parents.
- The intervention was simple to implement had good user acceptability and was cost saving.
- The intervention increased prescription uptake in the month prior to the return to school with 30% more prescriptions collected.
- There was no immediate effect in September but in the wider time intervals of September to December and September to August there was evidence of effect with a reduction in the mean number of medical contacts.
- The coding of the outcomes from the routine data was challenging and the assessment of adherence not possible.

For peer review only

1 INTRODUCTION

Asthma episodes and deaths are known to be seasonal [1]. A number of reports have shown peaks in asthma episodes in school-aged children associated with the return to school following the summer vacation [2–10]. Children returning to school are exposed to a variety of novel respiratory insults including allergens and viruses, at a time of changing climactic conditions. It has previously been shown that viral infection and allergen exposure in allergen sensitised asthmatics are associated with increased hospital admissions for acute asthma [11].

Our previous research [12] confirmed the increase in unscheduled medical contacts with children with asthma being approximately twice as likely as controls to have an unscheduled medical contact with their doctor around the time of the return back to school. In the same study it was found that in August, immediately preceding the return back to school, there were 25% fewer prescriptions for inhaled corticosteroids, compared to July and September. Patients who received a prescription for inhaled corticosteroids were less likely to have an unscheduled medical contact after the return to school.

Little is known about the factors that are associated with the drop in prescriptions in August. Research on adherence to paediatric asthma treatment in general has identified weak beliefs about the necessity of asthma medication as a key reason for non-adherence[13]. Given that asthma symptoms decline in the summer months this may lead to weaker beliefs about the necessity to take asthma medication. The GP letter was designed to address this belief by emphasising the importance of (re)taking asthma mediation prior to returning to school.

The current study is a cluster randomised trial to evaluate whether a letter sent from a GP at the start of the summer vacation reminding parents of children with asthma of the necessity of taking their asthma medication before the return back to school. The study evaluated whether the letter reduced unscheduled contacts after the return back to school and increased prescriptions in August.

2 RESEARCH AIMS AND OBJECTIVES

The aim of the study was to assess if a general practice delivered public health intervention (a letter sent from the GP to parents/carers of school-aged children with asthma) can reduce the number of unscheduled medical contacts per child after the school return.

For peer review only - http://bmjopen.bmj.com/site/about/guidelines.xhtml

3 METHODS

3.1 Study Design

The study was an open-label cluster randomised trial where GP practices were randomised to the intervention or usual care. The study protocol and HTA report have been published [14 15]. The effectiveness of the intervention was assessed on the basis of reduced unscheduled medical contacts after the return to school in September and prescription uptake prior in August. The primary study period was 1st – 30th September 2013 after the return to school. The extended study period was 1st September - 31st December 2013, since asthma-related appointments are more frequent in these months for children with asthma. The full follow-up period was 12 calendar months from 1st September 2013 to 31st August 2014. Prescription uptake and scheduled medical contacts such as asthma reviews were evaluated during the periods August 2013 and August 2013-July 2014, respectively,

A cluster randomised trial was chosen due to the nature of the condition of asthma. Even if the study design was individually randomised there would have been a need for the study to be randomised by household as siblings are likely to have asthma. A further consideration was that we wished for the intervention to represent possible routine care for future implementation. A practice level intervention would represent this.

The health economic analyses were based on a 12 month period from 1st August 2013 to 31st July 2014. The period starts a month earlier than the evaluation of medical contacts in order to incorporate the cost associated with delivering the intervention including any increase in prescriptions or medical contacts in response to the intervention that occurred during August 2013.

The primary outcome was the proportion of patients who had an unscheduled medical contact in September 2013.

The secondary outcomes evaluated included the number of unscheduled medical contacts in September 2013 and the number and proportion of any medical contacts (scheduled and unscheduled) in the same time interval as well as in the time intervals September-December

2013 and September 2013-August 2014. The analyses of the same outcomes were repeated for the other time intervals.

3.2 Participants

Participants were school-aged children with asthma, aged between 4 and 16, registered with a general practitioner. The primary analysis population was the intention to treat population (ITT) among children aged between 5 years and 16 years of age.

The choice of the 5-16 age group as the primary analysis population is due to the difficulty associated with making a diagnosis of asthma among children below this age [16 17]. Patients aged 4-5 were analysed separately to those aged 5-16 and are not included in this paper. Additional analyses were restricted specifically to children who had received a prescription for steroid inhalers in the previous year.

3.3 Interventions

Sites were randomly allocated to either: Intervention Group - sending out the letter or Control Group - standard care (no letter)

The intervention was a letter sent from a GP to the parents/carers of children with asthma reminding them to maintain their children's medication and collect a prescription if they were running low (see Appendix 1). It also advised that should their child have stopped their medication, it should be resumed as soon as possible

The letter template was developed based on standard letters already used in general practice and designed to address beliefs about the necessity of taking asthma medication before the return back to school. The wording of the letter had input from the study team, which includes a GP, Health Psychologist and Consultant Respiratory Paediatrician. The letter was also discussed in detail at two patient and public events, that included school-aged children with asthma and their parents [18 19 20].

The intervention letters were sent out the week commencing 29th July 2013 to obviate the distraction of planning for family holidays and yet leave enough time for parents and children to renew prescriptions and gain benefit from the medication. The letter and the timing of the

For peer review only - http://bmjopen.bmj.com/site/about/guidelines.xhtml

letter was decided following discussion with the Patients and Public Involvement (PPI) group [19].

3.4 Patient Involvement

There were three PPI consultation events with children with asthma and their parents. The first consultation event was funded by a grant by National Institute of Health Research (NIHR) Research Design Service for Yorkshire and the Humber prior to submission of the grant application in January 2011.

At this first consultation event it was agreed that a letter from their practice would be a useful reminder and not seen in any way as intrusive. A draft of the proposed letter was reviewed and the children fed back that they believed that the letter from their GP should be addressed to their parents rather than to themselves.

The second PPI consultation event was held after the grant was awarded in September 2012 [19]. At this meeting the intervention letter was finalised. The general feeling among the group was that the intervention did not adequately reflect the seriousness of asthma as a health condition. It was felt therefore that there was a danger that the intervention could be ignored by parents, or that the information it contained could be forgotten. The letter was amended to reflect this input.

The consultation event also discussed the timing of the intervention and it was proposed to send the intervention the first week of August. The event also reviewed the lay summary for the study and provided input to the logo for the study.

Two parents also agreed to join the trial steering committee for the study. At the first trial steering committee meeting it was agreed to bring the timing of the intervention forward by a week to the end of July, as asthma medication has a better chance of working the earlier it is used consistently.

A third PPI consultation event was held after the study had been completed which will be discussed in the discussion [21]. There is a web site where the PPI events are detailed

For peer review only

(http://www.sheffield.ac.uk/scharr/sections/dts/ctru/pleasant/ppi Assessed 8 December 2017). There has also been a separate publication on the first two PPI consultation events[20].

3.5 Ethical approval and research governance

Ethical approval for the study was given by South Yorkshire Research Ethics Committee on 25th October 2012 (reference number 12/YH/04). NHS Permissions to conduct the study was obtained for all the Primary Care Trusts (PCTs) in England and Health Boards in Wales.
Details of an amendment to the protocol are given in Appendix 2. The amendment was to extend the follow up period by one month to the end of September 2014.

The trial was registered with the International Standard Randomised Controlled Trial Register (ISRCTN) reference number ISRCTN 03000938.

3.6 Setting

The setting was primary care with the unit of cluster being general practices. Site eligibility required practices to be using the Vision IT software and be part of Clinical Practice Research Datalink (CPRD). Site recruitment was conducted by CPRD and the NIHR Primary Care Research Network with the PLEASANT study team [22].

3.7 CPRD recruitment

A practice recruitment pack, consisting of a detailed study information sheet and an expression of interest (EoI) form, was sent to all 433 practices contributing to CPRD in England and Wales at the time of recruitment [22]. Practices were also recruited through the primary care research network. Recruitment took place over a 7-month period from January 2013 to July 2013. For these practices to be in the trial they needed to join the CPRD.

3.8 Randomisation and blinding

After each practice gave verbal consent to participate in the trial they were randomised to either the intervention or usual care.[22] Randomisation was stratified by size of General Practice (i.e. the "list size") to ensure that there was an equal sample size – in terms of number of school-age children with asthma – in each arm of the trial. The randomisation sequence was generated by a statistician based within the Sheffield CTRU, using a blocked randomisation and allocation concealment was ensured by restricting access to the two CTRU

For peer review only - http://bmjopen.bmj.com/site/about/guidelines.xhtml

statisticians. Once practices had agreed to participate, their identifier and list size was forwarded to the trial statistician for randomisation to one of the two groups. The randomisation was then revealed to the study manager and research assistant. The study team were unblinded throughout the study but had no access to data until after a statistical analysis plan was developed and had no influence on data capture.

3.9 Data management

Data was collected through the CPRD which captures the coding for each consultation by staff in the practice. The medical consultations and diagnostic codes were reviewed to determine if each contact was a scheduled contact – such as a medicines review – or an unscheduled contact – such as an acute or an out of hours visit.

An independent GP adjudication panel was established to help in the coding. The adjudication panel met three times and did not have access to the randomisation group when reviewing the data. The adjudication panel reviewed and coded 4,600 unique terms into scheduled and unscheduled medical contacts. These terms accounted for 92% of all medical contacts but 17% of all terms used in the study. Terms not coded by the adjudication panel were coded as unscheduled. In addition, 7.9% of all contacts did not have any terms to indicate consultation type or diagnosis and free text was used in the database system to which the study team had no access. The adjudication panel advised to code these contacts as unscheduled.

3.10 Statistical methods

3.10.1 Analysis populations

The study was designed to detect a difference of 5% in the proportion of children who have an unscheduled medical contact (30 v 25%) with 90% power and two sided significance level of 5%, with an intra-class correlation (ICC) of 0.03 to account for clustering. Based on this we estimated that we required 70 practices per arm. It was anticipated that the sample size of 140 practices would equate to approximately 14,000 school-age children with asthma. We assumed equal cluster sizes in the sample size calculation. Sensitivity analyses indicated that the study was robust to the assumptions made for the ICC as well as to practices not sending the intervention and reducing the observed effect size [15].

For peer review only

Each of the outcomes were evaluated on for each of each subpopulations: children aged 5-16 (the primary analysis population) and children aged 5-16 who have prescriptions for steroid preventer. The analyses were restricted specifically to children who had received a prescription for preventer inhalers in the previous year as this was intended to identify the treatment effect in the population likely to receive most benefit.

The primary analyses of effectiveness were performed on the intention to treat (ITT) population. Analyses were also conducted on the per protocol (PP) population. The health economic analyses were based on the PP population. ITT analyses comprised all practices for whom data were obtained for the study period (see Section 4.3). There were two criteria for exclusion from PP analyses. First, practices that did not send intervention as requested the entire practice data was excluded from PP analyses. Second, individual children who were not sent the intervention letter. GPs were given discretion to withhold the letter from any children they believed were unsuitable. In such cases, the individual was excluded from PP analyses.

3.10.2 Analytical methods

The proportion of children having an unscheduled medical contact was analysed separately for each time period using logistic regression with the individual's age, sex, number of contacts the previous September as covariates, the trial arm (intervention or control) as a fixed effect, and the design/cluster effect of general practice as a random effect. The proportion of children having a prescription within each time period was analysed in the same manner. The number of unscheduled medical contacts made in each period by the children as well as the number of prescriptions ordered within a time period, were both analysed using a random effects negative binomial model in which the same covariates as above were included [15].

3.11 Health economic methods

An economic evaluation was undertaken to compare the incremental cost per quality adjusted life year (QALY) of the reminder letter versus standard care. The perspective of the analysis was that of the NHS (primary and secondary care resource-use based on available CPRD data and associated costs). We assumed the intervention would have no impact on mortality or

For peer review only

quality of life (utility) beyond 4 months and, as a result, QALYs were calculated for the four-month post-intervention time period. Costs were calculated for one year post-intervention to allow for changes in the timing of routine asthma care in response to the intervention to be distinguished from changes in the number of scheduled contacts.

Bootstrapped costs were evaluated 12 months post-intervention with one year linear regression-based baseline adjustment (BA). Costs for the letter intervention were based on a survey of participating practices which included questions on staff members involved as well as staff time [23]. Full details of the methods used in the economic analysis have been published in a separate paper [24].

3.12 Trial oversight

A Trial Steering Committee (TSC) was established to give oversight to the study. The TSC consisted of an independent chair (GP), two independent members (academic GP and statistician) and two lay members (parents of children with asthma) along with the Principal Investigator and key staff within the CTRU (as non-voting members). The role of the TSC was to provide supervision of the protocol and statistical analysis plan and to provide advice on, and monitor progress of, the trial.

4 TRIAL RESULTS

4.1 Recruitment and participant flow

The target sample size was 140 GP practices. In total, 142 practices agreed to take part in the study. Recruitment of GP practices was undertaken over a 7 month period, details of which have been published [22]. Of these practices, one (a control group practice with 99 children with asthma) withdrew consent after the start of the study for the data to be extracted and stored by the CPRD (independent of the study); this practice was excluded from all analyses. In total, 70 practices (comprising 5917 individuals) were randomised to the intervention (letter) and 71 practices (6262 individuals) to control. The CONSORT diagram is given in Figure 1 for the 12 months follow-up of the study.

4.2 Baseline characteristics

The descriptive statistics of the 12179 subjects and 141 practices are included in Table 1a and Table 1b. Summaries reported are stratified by intervention type and overall.

For peer review only

4.3 Number of participants and analysis subsets

For each study period, analyses were based only on practices that contributed data to the entirety of that period. In other words, if practices stopped submitting data to CPRD before the end of a given follow-up period they were excluded from all analyses for that time period. Practices that changed their software from the Vision IT system were no longer able to participate in CPRD and so withdrew from the study. The data from the practices until the time they withdrew was included in the statistical analysis. Details of the practices within the study during each time period are given in Appendix 3.

Figure 1 shows the flow of patients and practices for the primary analysis population (aged 5-16). Of the 456 practices invited, 433 were through the CPRD and 23 were through the primary care research network and joined the CPRD [22].

There were 786 GP exclusions in the intervention arm. There were zero GP exclusions in the control arm, as it was impossible for the GPs to exclude individuals from receiving letters when no patients in the control arm were due to receive a letter.

4.4 Clinical results

The primary time point for the analysis was September. Thus, in the primary analysis the proportion of individuals who had at least one unscheduled medical contact in September was 45.2% in the intervention arm, compared with 43.7% in the control arm (adjusted odds ratio (OR) = 1.09, 95% CI 0.96 to 1.25 (see Table 2a). The ICC for the primary analysis was 0.026 and was consistent with the estimate for the sample size calculation. In terms of mean contacts the number of unscheduled contacts are comparable (incidence rate ratio IRR=1.02 95% CI 0.94 to 1.12). The results are comparable for children receiving preventer medication (see Table 2b).

In the year following the intervention, there was evidence of a reduction in the mean number of medical contacts. As a consequence, the incidence ratio declines as longer time periods are analysed (see Table 2a), suggesting that the short-term increase in medical contacts in September is gradually outweighed by decreases in unscheduled contacts in the longer-term. There is a non-statistically significant 3% reduction in unscheduled contacts (IRR=0.97 with 95% CI 0.95 to 1.04), and a statistically significant 5% reduction in the total number of

For peer review only - http://bmjopen.bmj.com/site/about/guidelines.xhtml

medical contacts (IRR=0.95 with 95% CI 0.91 to 0.99), over the 12 months following the intervention.

Unscheduled medical contacts for children in the trial in the year before and after the intervention are presented in Figure 2a. A pronounced drop in unscheduled medical contacts can be seen in August 2012. After the return to school in September 2012 there is an increase in unscheduled medical contacts which peaks in October/November before reducing.

In 2013 there is a similar pattern to 2012 in the control arm. In contrast, in the intervention arm, although there is no immediate effect of the intervention in September, however, the peak in October/November is less pronounced than in the control arm.

The planned analysis was of prescriptions in August. This demonstrated that the intervention (letter) was associated with a statistically significant increase in the uptake of prescriptions in the month of August 2013 (see Table 3). In August, 876 (16.5%) patients in the intervention arm had at least one prescription compared with 703 (12.6%) in the control group (adjusted odds ratio 1.43, 95% CI 1.24 to 1.64); the total number of prescriptions was also higher (adjusted incidence rate ratio 1.31, 95% CI 1.17 to 1.48). In line with the increase in prescriptions, there was also a non-statistically significant increase in scheduled contacts made in August 2013, in terms of having at least one contact (adjusted odds ratio 1.13, 95% CI 0.84 to 1.52) and a statistically significant increase in terms of the mean number of scheduled contacts (IRR=1.17, 95% CI 1.06 to 1.29).

Preventer prescription collections for children in the trial in the year before and after the intervention are presented in Figure 2b. Mirroring the unscheduled contacts in August 2012, there is a reduction in prescriptions collected in this month. After the return to school in September there is an increase in prescriptions collected with a peak in the interval between October and December followed by a reduction.

For peer review only

In 2013 there is a similar pattern to 2012 in the control arm. In contrast, in the intervention arm, there is a marked increase in prescriptions in August 2013 that appears to continue into September before declining.

Per protocol population analyses were also conducted, with the results being broadly consistent with the intention to treat analyses reported above. However, there were larger effects for the increase in scheduled contacts and uptake of prescriptions in August, but smaller effects for unscheduled contacts and total medical contacts [25].

The analysis of respiratory relation contacts is given in Appendix 4 and Figure 3.

4.5 Health economic results

The full results of the economic evaluation have been published in a separate paper so only key BA base-case results are provided here [24]. The average cost per child of sending the intervention was £1.34 per child. The fall in medical contacts over one year described in the clinical results led through into the health economic assessment. A mean reduction in costs per child of £36.07 was estimated and there was 96.3% certainty of the intervention being cost saving. The economic evaluation estimated a mean QALY loss of 0.00017 which is practically zero.

5 DISCUSSION

Previous work has shown an increase in the number of unscheduled medical contacts by children in autumn months (September to December), which may be due to the start of the new school term [26]. By sending a letter at the start of the school holidays to remind children of the importance of taking their medication, it was hypothesised that this increase in unscheduled medical contacts may be averted. More specifically, it was predicted that a reminder letter would lead to a greater uptake of inhaler prescriptions in August that, in turn, would lead to increased adherence and, finally, fewer unscheduled medical appointments after the return to school.

There was evidence of an impact on the first part of this pathway as the intervention group demonstrated a higher uptake of prescriptions in August 2013. They also had a non-

For peer review only

statistically significant increase in the number of scheduled contacts in the same month. Data are not available to confirm actual medicine usage and, as a result, it is unclear whether the increased uptake also translated into an increased usage. The original plan was to assess this through the medicines possession ratio, which estimates the time a child has collected medication for over the time the child should have collected time for. However, this could not be estimated for these data due to inadequate recording of prescription data in the routine data. Further work is required to determine how to assess adherence using such routine data.

The primary endpoint was unscheduled medical contacts in September 2013, which coincided with the start of the new school term. There was no evidence of a reduction in the intervention group. In fact, there was a non-statistically significant increase in the proportion of children who had an unscheduled medical contact in September.

The increase could be caused by GPs needing to see certain patients before giving a new prescription if they had not had a prescription recently. Evidence to support this is a *post hoc* observation that for children who had collected a prescription within the last 3 months prior to the start of the study, there was an increase in unscheduled contacts in September with 55.2% of patients in the intervention arm seeing their GP compared to 54.3% for controls. For patients whose last prescription was 3-6 months prior to the start of the study, the difference between the arms was greater with 42.1% in the intervention arm seeing their GP in September compared to 39.7% for controls.

The way the unscheduled contacts were coded could have also impacted on the outcome. The intervention increased prescription update and collection of a prescription for asthma was coded as an unscheduled medical contact.

A further explanation for the lack of effect of the intervention on unscheduled contacts in September is that September was too early to make an assessment of efficacy. Given that exposure to infections may take some time to have an impact on asthma symptoms in school-age children, it is possible that making the primary outcome period the first 4 weeks after returning to school was too soon to observe an effect of the intervention. It is interesting that an effect in favour of the intervention was demonstrated when the measurement period was extended both to December and to the following August. In the extended period both the total number of contacts per child (i.e. scheduled plus unscheduled) and unscheduled contacts

For peer review only

were lower in the intervention group than in the control over the extended study period (September to December 2013) and the full year (September 2013 to August 2014), although only the effect on the total number of medical contacts for the full year was statistically significant.

The effects on medical contacts, though small, are potentially clinically important given the effect the intervention had on prescriptions. The expectation was an increase in prescription update would lead to a reduction in medical contacts. The results are in line with this expectation. Moreover, the minimal cost associated with the intervention meant the intervention was found to have a high probability of being cost-saving overall. With such a relatively low cost intervention, £1.34 per child, and an average cost for an unscheduled surgery visit circa £50, an intervention would only need to reduce the number of contacts by 3 per year for an average practice with 85 asthmatic children to be cost neutral. The evidence from the trial is that contacts are reduced 0.6 per child in the 12 months after intervention, or 51 per year for an average practice of 85 children.

The economic analysis (which used data over a 12 month period from August 2013 to July 2014) estimated a mean cost saving across the base case of £36.07 per child. So, although the study did not have a significant effect for the primary endpoint, the minimal cost associated with the intervention meant the intervention was found to have a high probability (96.3%) of being cost-saving overall, primarily due it its effect on reducing the total number of medical contacts over the following year. In the UK alone there are over one million children with asthma. The intervention thus has the potential to provide health service savings if implemented.

The results were discussed with children with asthma and their parents at a PPI consultation event [21]. At the event attendees felt that the savings per child was an important result and suggested that the impact of the intervention could have been greater if it had been repeated over a number of years. The letter could then assist parents and children as they plan for the school return each year.

There is evidence of good user acceptability with over half the practices who responded to a survey reporting that they had repeated the intervention the year after the study [23]. Once the intervention is set up for one year, the costs then associated with sending it out in subsequent

For peer review only

years are less given that many of the school age children with asthma will be the same from year to year.

There were methodological issues associated with the cluster randomised trial. Although there were 12,179 children with asthma in the study, there were only 141 GP practices, which was the unit of randomisation. With 141 GP practices there is a chance of random differences between the two intervention arms. Any random differences could be compounded by the fact that children with asthma and the common medical practice undertaken to manage asthma would tend to be more alike within practices than between practices. This may affect the clinical outcomes.

The strengths of the study were that the intervention was evaluated in a relatively large trial population of children within a primary care setting within a single year. In addition, the procedures used in the study were the same as those that would be used in clinical practice and so implementation into routine care is straightforward.

The study had a highly efficient innovative study design that used routine data for all outcomes and the delivery of the intervention was centrally automated through the CPRD. By our own estimation a substantial six-figure sum is saved compared to a trial where GP practices would need to be visited to collect the data. There were additional practical advantages in using routine data. For example, the planning of data collection was relatively straightforward to schedule and the collection of baseline data could be done retrospectively once practices had entered the trial.

This final strength of the trial is also a weakness. Using routine data made the assessment of unscheduled contacts within the trial difficult - especially for an intervention which increased initial medical activity through the collection of prescriptions. In this study, it would have been helpful for two additional questions to be asked to facilitate evaluation of the intervention: Was the contact unscheduled? Was the contact respiratory related?

The study adds to the current literature by demonstrating that an easy to implement intervention of a simple letter from a GP to the parents of a children with asthma can assist in the self-management of the condition by increasing prescription uptake and consequently reducing medical contacts. Over 90% of medical contacts are in a primary care setting and

For peer review only

yet there is a paucity of evaluations in this setting. This has demonstrated that using routine data collected through the CPRD within a cluster randomised trial is feasible and has shown the advantages and disadvantages of this approach.

6 CONCLUSIONS

The intervention succeeded in increasing the number of children collecting a prescription in August. The intervention did not reduce unscheduled care as expected in September, which was the primary endpoint, although in the year following the intervention, it had a statistically significant, and potentially clinically important, effect on reducing total medical contacts. This is reflected in the health economic evaluation which, overall, showed that the intervention had a high probability of giving a cost saving.

With the evidence from the trial of an increase in August of both prescription collection and evidence of cost reduction practices may wish to implement the intervention, particularly practices with high rates of unscheduled medical care.

7 REFERENCES

1. Campbell, M. J., Holgate, S. T. & Johnston, S. L. Trends in asthma mortality. *BMJ* **315,** 1012 (1997).

2. Storr, J, Lenney, W. School holidays and admissions with asthma. *Arch. Dis. Child.* **64,** 103–7 (1989).

3. Grech, V., Balzan, M. & Distefano, S. Paediatric Wheezy Admissions at and around School Holiday Periods. *Malta Med. J.* **16,** 23–26 (2004).

4. Kimes, D, Levine, E, Timmins, S, Weiss, SR, Bollinger, ME, Blaisdell, C. Temporal dynamics of emergency department and hospital admissions of pediatric asthmatics. *Environ. Res.* **94,** 7–17 (2004).

5. Kimbell-Dunn, M., Pearce, N. & Beasley, R. Seasonal variation in asthma hospitalizations and death rates in New Zealand. *Respirology* **5,** 241–6 (2000).

6. Gergen, P. J., Mitchell, H. & Lynn, H. Understanding the seasonal pattern of childhood asthma: results from the National Cooperative Inner-City Asthma Study (NCICAS). *J. Pediatr.* **141,** 631–6 (2002).

7. Harju, T., Keistinen, T., Tuuponen, T. & Kivelä, S. L. Seasonal variation in childhood

For peer review only

asthma hospitalisations in Finland, 1972-1992. *Eur. J. Pediatr.* **156,** 436–9 (1997).

8. Julious, S. A., Osman, L. M. & Jiwa, M. Increases in asthma hospital admissions associated with the end of the summer vacation for school-age children with asthma in two cities from England and Scotland. *Public Health* **121,** 482–4 (2007).

9. Silverman, R. A., Stevenson, L. & Hastings, H. M. Age-related seasonal patterns of emergency department visits for acute asthma in an urban environment. *Ann. Emerg. Med.* **42,** 577–586 (2003).

10. Fleming, D. M., Cross, K. W., Sunderland, R. & Ross, A. M. Comparison of the seasonal patterns of asthma identified in general practitioner episodes, hospital admissions, and deaths. *Thorax* **55,** 662–5 (2000).

11. Murray, CS, Poletti, G, Kebadze, T, Morris, J, Woodcock, A, Johnston, S. & Custovic, A. Study of modifiable risk factors for asthma exacerbations: virus infection and allergen exposure increase the risk of asthma hospital admissions in children. *Thorax* **61,** 376–82 (2006).

12. Julious, S. A., Campbell, M. J., Bianchi, S. M. & Murray-Thomas, T. Seasonality of medical contacts in school-aged children with asthma: association with school holidays. *Public Health* **125,** 769–76 (2011).

13. Drotar, D; Bonner, M. Influences on Adherence to Pediatric Asthma Treatment: A Review of Correlates and Predictors. *J. Dev. Behav. Pediatr* **30,** 574–82 (2009).

14. Julious SA, Horspool MJ, Davis S, Bradburn M, Norman P, Shephard Neil, Cooper CL, Smithson, WH, Boote J, Elphick Heather, Loban A, Franklin M, Kua WS, May Robin,; Campbell, Jennifer; Williams, Rachael; Rex, Saleema; Bortolami, O. PLEASANT: Preventing and Lessening Exacerbations of Asthma in School-age children Associated with a New Term - a cluster randomised controlled trial and economic evaluation. *Health Technol. Assess. (Rockv).* **20,** 1–154 (2016).

15. Horspool, M. J. *et al.* Preventing and lessening exacerbations of asthma in school-age children associated with a new term (PLEASANT): study protocol for a cluster randomised control trial. *Trials* **14,** 297 (2013).

16. British Thoracic Society / Scottish Intercollegiate Guideline. *British Guideline on the Management of Asthma: A national clinical guideline.* (2012).

17. Bush, A. Diagnosis of asthma in children under five. *Prim. Care Respir. J.* **16,** 7–15

For peer review only

(2007).

18. Julious SA, Lai J, Boote J, Elphick H, S. H. Report of a patient and public involvement consultation event with children with asthma and their parents to review a proposal for a primary care intervention around the September school return. *Univ. Sheff. ScHARR Rep. Ser. ISBN 1 900752 18 2* **25,** ISBN 1 900752 18 2 (2011).

19. Julious, SA, Horspool M J, Boote, J. PLEASANT TRIAL: 2nd Consultation event with children with asthma and their parents. *Univ. Sheffield. ScHARR Rep. Ser. No 28. ISBSN 1 900752 14 X* (2012).

20. Boote, J, Julious, S, Horspool, M, Elphick, H, Smithson, WH, Norman, P. PPI in the PLEASANT trial: involving children with asthma and their parents in designing an intervention for a randomised controlled trial based within primary care. *Prim. Health Care Res. Dev.* 1–13 (2016). doi:10.1017/S1463423616000025

21. Simpson, RM, Julious, SA, Mooney, C, Norman, P. PLEASANT TRIAL: 3rd Consultation event with children with asthma and their parents. *Univ. Sheff. ScHARR Rep. Ser. No 32. ISBN 1900752344.* (2015).

22. Horspool, MJ, Julious, SA, Mooney, C, May, R, Sully, B, Smithson, W. Preventing and Lessening Exacerbations of Asthma in School-aged children Associated with a New Term (PLEASANT): Recruiting Primary Care Research Sites-the PLEASANT experience. *NPJ Prim. care Respir. Med.* **25,** 15066 (2015).

23. Hatfield, I., Julious, S.A., Davis, S., Horspool, M.J., Norman, P., Mooney, C. An assessment of the resources used by General Practices in the intervention arm of the PLEASANT study in sending out the intervention. *Univ. Sheffield. ScHARR Rep. Ser. No 30. ISBSN 1 900752 24 7* (2015).

24. Franklin, M., Davis, S., Horspoo, l M., Kua, W.S., Julious, S. Economic Evaluations Alongside Efficient Study Designs Using Large Observational Datasets: the PLEASANT Trial Case Study. *Pharmacoeconomics* **35,** 261–73 (2017).

25. Julious, S.A., Horspool, M.J., Davis, S. Blackburn M Norman P, Shephard N, Cooper CL, Smith WH, Boote J, Elphick H, Loban A, Franklin M, Kua WS, May R, Campbell J, Williams R, Rex S, B. O. PLEASANT: Preventing and Lessening Exacerbations of Asthma in School-age children Associated with a New Term a cluster randomised controlled trial and economic evaluation. *Health Technol. Assess. (Rockv).* **20,** 1–154 (2016).

For peer review only

26. Julious, S. A., Campbell, M. J., Bianchi, S. M. & Murray-Thomas, T. Seasonality of medical contacts in school-aged children with asthma: association with school holidays. *Public Health* **125,** 769–76 (2011).

For peer review only

Study Registration

ISRCTN03000938 - assigned 19/10/2012

Contributors

Steven A. Julious, Professor Medical Statistics; Michelle J Horspool, Trial Manager; Sarah Davis, Health Economist; Matthew Franklin, Health Economist, together produced the first draft of the report.

The following conceived of or designed the work: Steven A. Julious, Professor of Medical Statistics; Michelle J Horspool, Trial Manager; Sarah Davis, Health Economist; W Henry Smithson, Prof. Primary Care; Healther Elphick, Consultant Respiratory Paediatrician; Paul Norman, Prof Health Psychology; Cindy L Cooper, Sheffield CTRU Director.

The following were involved in the interpretation of data for the work: Steven A Julious, Prof Medical Statistics; Michelle Horspool, Trial Manager; W Henry Smithson; Prof. Primary Care; Healther Elphick, Consultant Respiratory Paediatrician.

The following were involved in the analysis of data: Oscar Bortolami, Medical Statistician; Sarah Davis, Health Economist; Matthew Franklin, Health Economist and Rebecca Simpson, PhD student.

The following drafted the paper: Steven A. Julious, Professor Medical Statistics; Michelle J Horspool, Trial Manager; Sarah Davis, Health Economist; Matthew Franklin, Health Economist.

The following reviewed the work critically for important intellectual content. Steven A. Julious, Professor Medical Statistics; Michelle J Horspool, Trial Manage, Oscar Bortolami, Medical Statistician; Sarah Davis, Health Economist; Matthew Franklin, Health EconomistW Henry Smithson, Prof Primary Care; Heather Elphick, Consultant Respiratory Paediatrician; Paul Norman, Prof of Health Psychology; Cindy L Cooper, CTRU Director.

All authors agree to be accountable for all aspects of the work in ensuring that questions related to the accuracy or integrity of any part of the work are appropriately investigated and resolved.

Declaration of interests

We have read and understood BMJ policy on declaration of interests and declare that we have no competing interests.

For peer review only

All authors have completed the ICMJE uniform disclosure form at www.icmje.org/coi_disclosure.pdf and declare: no support from any organisation for the submitted work; no financial relationships with any organisations that might have an interest in the submitted work in the previous three years; no other relationships or activities that could appear to have influenced the submitted work.

Funding for this study was provided by the Health Technology Assessment programme of the National Institute for Health Research Programme (Grant 11/01/10)

Exclusive License

The Corresponding Author has the right to grant on behalf of all authors and does grant on behalf of all authors, a worldwide licence to the Publishers and its licensees in perpetuity, in all forms, formats and media (whether known now or created in the future), to i) publish, reproduce, distribute, display and store the Contribution, ii) translate the Contribution into other languages, create adaptations, reprints, include within collections and create summaries, extracts and/or, abstracts of the Contribution, iii) create any other derivative work(s) based on the Contribution, iv) to exploit all subsidiary rights in the Contribution, v) the inclusion of electronic links from the Contribution to third party material where-ever it may be located; and, vi) licence any third party to do any or all of the above

Transparency declaration

The lead author, and manuscript guarantor, affirms that this manuscript is an honest, accurate, and transparent account of the study being reported; that no important aspects of the study have been omitted; and that any discrepancies from the study as planned (and, if relevant, registered) have been explained.

Data Sharing

Access to patient level data is provided by the CPRD for health research purposes and is dependent on approval of a study protocol by the MHRA Independent Scientific Advisory Committee (ISAC). More information on ISAC and the protocol submission process can be found at: www.cprd.com/isac (date accessed 18 April 2017).

For peer review only

Sponsor

The study was sponsored by Sheffield Clinical Commissioning Group. The authors has freedom to act in the writing and submission of the paper.

Acknowledgments

We gratefully acknowledge the hard work, support and advice from the following: Hilary Pinnock, (Professor, University of Edinburgh) for her advice; Gerry McCann, Zaynah Gurreebun, Rachael Williams, Robin May and Jennifer Campbell, Clinical Practice Research Datalink (Clinical Practice Research Datalink, CPRD) for their contribution to site recruitment and to Tjeer Van Staa (CPRD) for his advice on CPRD; Cara Mooney, David White (University of Sheffield) for support with study set up, site recruitment and site set up and close down; Dan Beever and Helen Wakefield for administrative and clerical support; Wei Sun Kua, for her Health Economic contribution; Jonathan Boote, as PPI Lead; Mike Bradburn and Neil Shephard for the statistical input and Amanda Loban for data management.

We are very grateful to the children and parents who took part in the three patient and public events through all stages of the study for their advice and input.

We would like to especially thank the GP adjudication panel Dr Mark Boon (Conisbrough Group Practice), Dr Karen Forshaw (Bentley Surgery), Dr Julie Hackney (The Avenue Surgery) for their advice, steer and valued contributions.

We offer special thanks to the members of our oversight committee: Trial Steering Committee: Dr Steve Holmes (Independent Chair, General Practitioner), Prof Andrew Wilson (Independent Academic General Practitioner, University of Leicester), Dr Martyn Lewis (Independent Statistician, Keele University), Zaida Bibi (Independent Parent Representative), and Camilla Mills (Independent Parent Representative).

The views expressed in this report are those of the authors and not necessarily those of the National Institute for Health Research Health Technology Assessment programme. Any errors are the responsibility of the authors.

For peer review only

Figure 1. CONSORT Diagram

Figure 2. Unscheduled medical contacts and prescriptions over time

Figure 3. Unscheduled respiratory medical contacts

For peer review only

Table 1: Descriptive statistics of patients and surgeries

a) Descriptive statistics of sex (frequencies and percentages reported) and age (mean, SD, median, interquartile range and range reported). Statistics produced at subject level.

Variable	Letter (N=5917)	No Letter (N=6262)	Total (N=12179)
Sex	Male (%)<br>3505 (59.24)<br><br>Female (%)<br>2412 (40.76)	Male (%)<br>3749 (59.87)<br><br>Female (%)<br>2513 (40.13)	Male (%)<br>7254 (59.56)<br><br>Female (%)<br>4925 (40.44)
Age	Mean (SD)<br>10.51 (3.29)<br>Median (IQR)<br>10.80 (7.88 - 15.97)<br>Range<br>4.05 - 15.97	Mean (SD)<br>10.55 (3.30)<br>Median (IQR)<br>10.89 (7.80 - 15.97)<br>Range<br>4.05 - 15.97	Mean (SD)<br>10.53 (3.30)<br>Median (IQR)<br>10.89 (7.80 - 15.97)<br>Range<br>4.05 - 15.97
Unscheduled Contacts September 2012	Mean (SD)<br>0.84 (1.20)<br>Median (IQR)<br>0.00 (0.00 – 1.00)<br>Range<br>0.00 - 10.00	Mean (SD)<br>0.88 (1.26)<br>Median (IQR)<br>0.00 (0.00 – 1.00)<br>Range<br>0.00 – 12.00	Mean (SD)<br>0.86 (1.23)<br>Median (IQR)<br>0.00 (0.00 – 1.00)<br>Range<br>12.00
Unscheduled Contacts September – December 2012	Mean (SD)<br>3.65 (3.34)<br>Median (IQR)<br>3.00 (1.00 – 5.00)<br>Range<br>0.00 – 31.00	Mean (SD)<br>3.78 (3.66)<br>Median (IQR)<br>3.00 (1.00 – 5.00)<br>Range<br>0.00 - 51.00	Mean (SD)<br>3.71 (3.51)<br>Median (IQR)<br>3.00 (1.00 – 5.00)<br>Range<br>0.0 51.00

b) Descriptive statistics of size (mean, SD, median, interquartile range and range reported). Statistics produced at surgery level.

Variable	Letter (N=70)	No Letter (N=71)	Total (N=141)
Size	Mean (SD)<br>85 (44)<br>Median (IQR)<br>80 (49 - 114)<br>Range<br>4-209	Mean (SD)<br>88 (64)<br>Median (IQR)<br>75 (41 - 107)<br>Range<br>10-293	Mean (SD)<br>86 (55)<br>Median (IQR)<br>76 (45-113)<br>Range<br>4-293

For peer review only

Table 2. Analysis of unscheduled and total medical contacts

A. For all children in the intent to treat population.

	Time Period	Treatment Arm* Intervention (%)	Control (%)	Odds-Ratio+	95% Confidence Interval	Treatment Arm* Intervention (Mean)	Control (Mean)	Incidence Ratio+	95% Confidence Interval
Unscheduled Contacts	Sep	45.2	43.7	1.09	0.96 to 1.25	0.81	0.81	1.02	0.94 to 1.12
	Sep-Dec	80.1	79.1	1.10	0.96 to 1.26	3.19	3.32	0.98	0.93 to 1.04
	Sep-Aug	93.1	93.3	0.97	0.82 to 1.15	9.08	9.37	0.97	0.95 to 1.04
Total Contacts	Sep	57.8	58.4	0.99	0.80 to 1.22	1.05	1.10	0.97	0.87 to 1.07
	Sep-Dec	89.3	88.4	1.06	0.89 to 1.27	4.31	4.43	0.95	0.90 to 1.02
	Sep-Aug	96.6	96.4	0.89	0.71 to 1.12	11.52	12.08	0.95	0.91 to 0.99

* the proportions and means are simple summary statistics
+ the odds-ratios and incidence ratios with the corresponding confidence intervals are from a formal statistical analysis allowing for covariates and the effect of clustering.

B. For children receiving preventer medication in the intent to treat population.

	Time Period	Treatment Arm* Intervention (%)	Control (%)	Odds-Ratio+	95% Confidence Interval	Treatment Arm* Intervention (Mean)	Control (Mean)	Incidence Ratio+	95% Confidence Interval
Unscheduled Contacts	Sep	46.3	45.4	1.07	0.94 to 1.23	0.83	0.84	1.01	0.92 to 1.10
	Sep-Dec	81.3	81.4	1.04	0.90 to 1.21	3.27	3.44	0.97	0.92 to 1.03
	Sep-Aug	93.9	94.6	0.84	0.69 to 1.02	9.31	9.71	0.98	0.92 to 1.14
Total Contacts	Sep	59.1	60.4	0.97	0.79 to 1.21	1.08	1.14	0.96	0.86 to 1.07
	Sep-Dec	90.4	90.5	0.98	0.81 to 1.19	4.43	4.70	0.95	0.89to 1.01
	Sep-Aug	97.1	97.3	0.81	0.64 to 1.01	11.82	12.53	0.96	0.90 to 1.12

* the proportions and means are simple summary statistics
+ the odds-ratios and incidence ratios with the corresponding confidence intervals are from a formal statistical analysis allowing for covariates and the effect of clustering.

For peer review only

Table 3. Analysis of prescription and scheduled contacts for August.

		Treatment Arm*				Treatment Arm*			
		Intervention (%)	Control (%)	Odds-Ratio$^+$	95% Confidence Interval	Intervention (Mean)	Control (Mean)	Incidence Ratio$^+$	95% Confidence Interval
Prescriptions	All Children	16.5	12.6	1.43	1.24 to 1.64	0.17	0.13	1.31	1.17 to 1.48
	Preventer	17.3	13.4	1.41	1 23 to 1.63	0.18	0.14	1.30	1.16 to 1.47
Scheduled	All Children	14.3	13.9	1.13	0.84 to 1.52	0.17	0.16	1.17	1.06 to 1.29
Contacts	Preventer	14.8	14.4	1.14	0.84 to 1.54	0.18	0.17	1.17	1.06 to 1.29

* the proportions and means are simple summary statistics

+ the odds-ratios and incidence ratios with the corresponding confidence intervals are from a formal statistical analysis allowing for covariates and the effect of clustering.

For peer review only

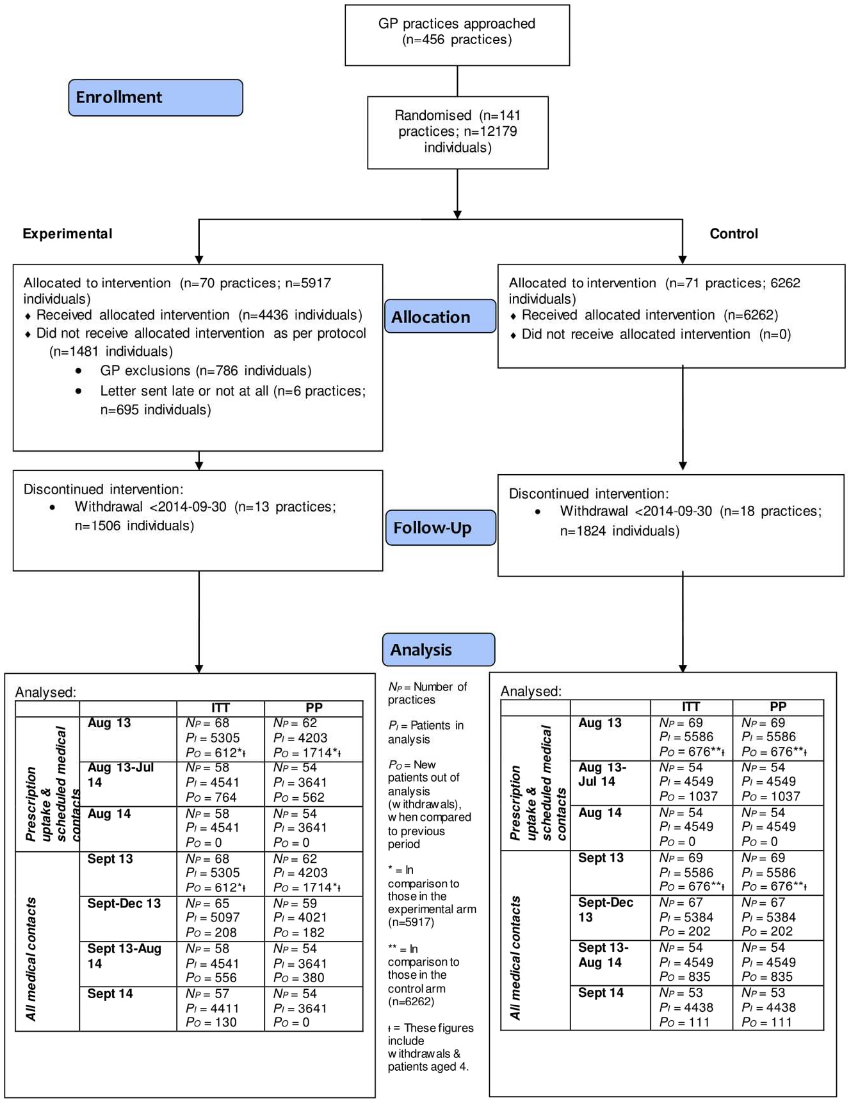

140x198mm (300 x 300 DPI)

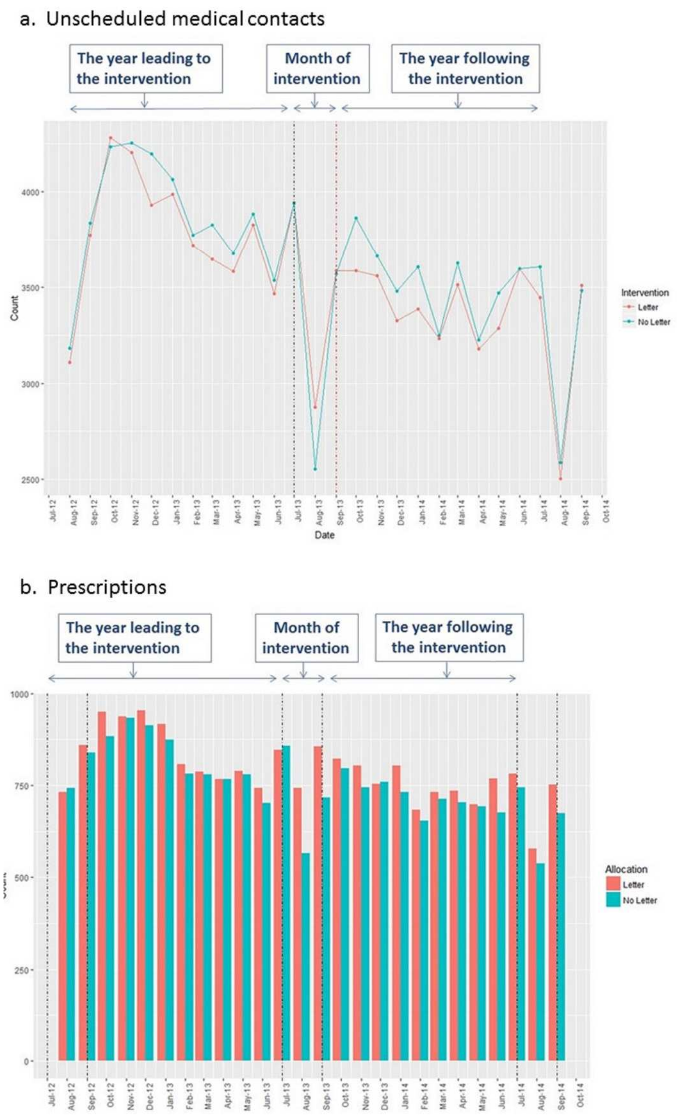

Figure 2. Unscheduled medical contacts and prescriptions over time

304x541mm (300 x 300 DPI)

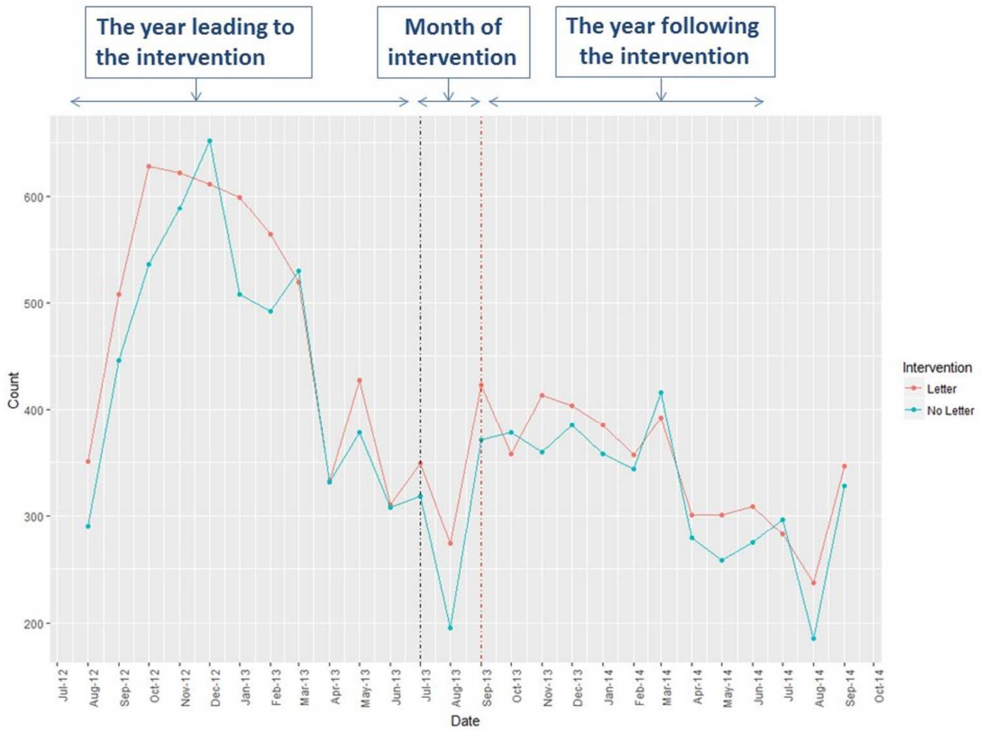

Figure 3. Unscheduled respiratory medical contacts

406x304mm (300 x 300 DPI)

1 APPENDICES

1.1 Appendix 1 . Trial Intervention

GP letterhead

< Address line 1>

< Address line 2>

< Address line 3>

< Address line 4>

<Insert Date>

Dear Parent

Please read this important letter regarding your child's asthma

It is really important that your child continues to take their asthma medication during the summer holidays. Returning to school is a time when asthma can get worse and make children and young people with asthma poorly. This may be due to contact with infections at the start of the new school year.

To reduce the chances of getting poorly when they return to school, your child should continue to take their asthma medication as prescribed by their GP or practice nurse. If your child has stopped taking their medication over the summer holidays it is important to start it again as soon as possible. If they are short of medication, or you are not sure of the proper dose, please get in touch with the practice.

Yours sincerely

<Name of Doctor>

For peer review only

For peer review only - http://bmjopen.bmj.com/site/about/guidelines.xhtml

1.2 Appendix 2. Changes to Protocol

Changes to Protocol		REC approval date	Approved by
Protocol Version 2 (14.05.15): This version included an additional secondary outcome to include data up to September 2014, to see if the effect from September 2013 is maintained when there is no study intervention thus extending the follow up period by one month (see section 2.1.5).	Agreed as a 2-month non-cost contact variation by HTA 02/02/2015	25th May 2014	NRES Committee Yorkshire & Humber – South Yorkshire

For peer review only

For peer review only - http://bmjopen.bmj.com/site/about/guidelines.xhtml

1.3 Appendix 3 - Practice Withdrawal and Adherence to Protocol

Table 4 provides the number of practices and the number of individuals aged 5-16 (the primary analysis population) included for each time period.

Table 4: Number of practices and individuals included within each time period

	**Letter**		**No letter**	
	**Practices**	**Individuals 5-16**	**Practices**	**Individuals 5-16**
Prescription uptake and scheduled medical contacts				
August 2013	68	5305	69	5586
August 2013-July 2014	58	4541	54	4549
August 2014	58	4541	54	4549
All medical contacts				
September 2013 (Primary study period)	68	5305	69	5586
September to December 2013 (extended study period)	65	5097	67	5384
September 2013-August 2014 (twelve month study period)	58	4541	54	4549
September 2014 (Echo sub-study)	57	4411	53	4438

For peer review only

1.4 Adherence to protocol

Of the 70 intervention practices, 2 did not send letters to any of the patients identified and 4 sent the intervention out late on the 6th, 8th, 12th and 23th of August. In addition, GPs were given discretion to withhold the letter from any children they believed were unsuitable candidates; among the remaining 64 practices (5222 individuals), letters were not sent to 786 children. These individuals were included in the primary ITT analyses but excluded from Per Protocol analyses.

For peer review only

For peer review only - http://bmjopen.bmj.com/site/about/guidelines.xhtml

1.5 Appendix 4 - Analysis of Respiratory Contacts

1.5.1 Analysis of unscheduled respiratory related contacts

The unscheduled medical respiratory related contacts are presented in Figure 3. The baseline data for the 12 months leading up to the intervention suggest that the practices randomised the intervention arm have more unscheduled respiratory contacts than the control arm. This feeds through into the summary statistics given in Table 5 which show more contacts in the intervention arm. When compared to Table 4 one can see that approximately 5% of medical contacts are respiratory-related in the analysis.

The adjusted analyses in Table 5 has the corresponding baseline term in the model. The analyses infer an increase in respiratory-related medical contacts. It should be noted though that the definition of a respiratory-related contact for the analysis included that a prescription for an asthma medication was given in the medical contact. The intervention has increased asthma prescriptions and so it is likely that a proportion of these contacts are associated with the increase in prescriptions.

To further explain the results, Table 6 breaks down of the type of respiratory-related contact by the time period. We can see here that in terms of total number of respiratory contacts the mean number of contacts per child is consistent between groups. It is likely that the intervention led to a greater proportion of respiratory contacts being coded as unscheduled. For September45% of all respiratory contacts in the intervention arm were coded as unscheduled compared with 35% in the control arm. This could be due to a prescription being collected in the contact – if the medical contact had no specific code to assign it as a scheduled contact, but had a prescription associated with it, then it would be coded as unscheduled.

For peer review only - http://bmjopen.bmj.com/site/about/guidelines.xhtml

Table 5. Analysis of unscheduled respiratory related medical contacts

	Time Period	Treatment Arm* Intervention (%)	Treatment Arm* Control (%)	Odds-Ratio+	95% Confidence Interval	Treatment Arm* Intervention (Mean)	Treatment Arm* Control (Mean)	Incidence Ratio+	95% Confidence Interval
All Children	Sep	5.3	4.2	1.30	1.03 to 1.66	0.06	0.05	1.30	1.02 to 1.66
	Sep-Dec	18.4	16.7	1.13	0.95 to 1.33	0.23	0.21	1.10	0.95 to 1.27
	Sep-Aug	38.0	35.3	1.05	0.87 to 1.33	0.57	0.53	1.04	0.90 to 1.20
Children Receiving Preventers	Sep	5.5	4.4	1.30	1.02 to 1.66	0.06	0.05	1.30	1.01 to 1.66
	Sep-Dec	19.2	17.4	1.13	0.96 to 1.34	0.24	0.22	1.10	0.95 to 1.28
	Sep-Aug	39.3	36.9	1.06	0.87 to 1.30	0.59	0.55	1.06	0.92 to 1.23

* the proportions and means are simple summary statistics

+ the odds-ratios and incidence ratios with the corresponding confidence intervals are from a formal statistical analysis allowing for covariates.

Table 6. Respiratory related medical contacts by type of contacts and time period

	Treatment Arm	Type of Contact Total	Type of Contact Scheduled	Type of Contact Unscheduled
Sep	Intervention	0.13	0.07	0.06
	Control	0.13	0.09	0.05
Sep-Dec	Intervention	0.56	0.33	0.23
	Control	0.56	0.35	0.21
Sep-Aug	Intervention	1.43	0.86	0.57
	Control	1.44	0.91	0.53

Note that total contacts=scheduled+unscheduled. There is rounding error in the table for row 2 as 0.134=0.088+0.046

For peer review only - http://bmjopen.bmj.com/site/about/guidelines.xhtml

For peer review only

Table 1: CONSORT 2010 checklist of information to include when reporting a cluster randomised trial

Section/Topic	Item No	Standard Checklist item	Extension for cluster designs	Page No *
**Title and abstract**				
	1a	Identification as a randomised trial in the title	Identification as a cluster randomised trial in the title	1
	1b	Structured summary of trial design, methods, results, and conclusions (for specific guidance see CONSORT for abstracts)[1,2]	See table 2	2
**Introduction**				
**Background and objectives**	2a	Scientific background and explanation of rationale	Rationale for using a cluster design	5
	2b	Specific objectives or hypotheses	Whether objectives pertain to the the cluster level, the individual participant level or both	4
**Methods**				
**Trial design**	3a	Description of trial design (such as parallel, factorial) including allocation ratio	Definition of cluster and description of how the design features apply to the clusters	5
	3b	Important changes to methods after trial commencement (such as eligibility criteria), with reasons		N/A
**Participants**	4a	Eligibility criteria for participants	Eligibility criteria for clusters	6
	4b	Settings and locations where the data were collected		8
**Interventions**	5	The interventions for each group with sufficient details to allow replication, including how and when they were actually administered	Whether interventions pertain to the cluster level, the individual participant level or both	6
**Outcomes**	6a	Completely defined pre-specified primary and secondary outcome measures, including how and	Whether outcome measures pertain to the cluster level, the individual participant level or both	5-6

		when they were assessed		
	6b	Any changes to trial outcomes after the trial commenced, with reasons		**15**
**Sample size**	7a	How sample size was determined	Method of calculation, number of clusters(s) (and whether equal or unequal cluster sizes are assumed), cluster size, a coefficient of intracluster correlation (ICC or *k*), and an indication of its uncertainty	9
	7b	When applicable, explanation of any interim analyses and stopping guidelines		**N/A**
**Randomisation:**				
**Sequence generation**	8a	Method used to generate the random allocation sequence		**8-9**
	8b	Type of randomisation; details of any restriction (such as blocking and block size)	Details of stratification or matching if used	8-9
**Allocation concealment mechanism**	9	Mechanism used to implement the random allocation sequence (such as sequentially numbered containers), describing any steps taken to conceal the sequence until interventions were assigned	Specification that allocation was based on clusters rather than individuals and whether allocation concealment (if any) was at the cluster level, the individual participant level or both	8-9
**Implementation**	10	Who generated the random allocation sequence, who enrolled participants, and who assigned participants to interventions	Replace by 10a, 10b and 10c	8-9
	10a		Who generated the random allocation sequence, who enrolled clusters, and who assigned clusters to interventions	8-9
	10b		Mechanism by which individual participants were included in clusters for the purposes of the trial (such as complete	9-10

For peer review only

			enumeration, random sampling)	
	10c		From whom consent was sought (representatives of the cluster, or individual cluster members, or both), and whether consent was sought before or after randomisation	12
**Blinding**	11a	If done, who was blinded after assignment to interventions (for example, participants, care providers, those assessing outcomes) and how		**8-9**
	11b	If relevant, description of the similarity of interventions		**N/A**
**Statistical methods**	12a	Statistical methods used to compare groups for primary and secondary outcomes	How clustering was taken into account	9-11
	12b	Methods for additional analyses, such as subgroup analyses and adjusted analyses		**9-11**
**Results**				
**Participant flow (a diagram is strongly recommended)**	13a	For each group, the numbers of participants who were randomly assigned, received intended treatment, and were analysed for the primary outcome	For each group, the numbers of clusters that were randomly assigned, received intended treatment, and were analysed for the primary outcome	11
	13b	For each group, losses and exclusions after randomisation, together with reasons	For each group, losses and exclusions for both clusters and individual cluster members	11
**Recruitment**	14a	Dates defining the periods of recruitment and follow-up		**11**
	14b	Why the trial ended or was stopped		**N/A**
**Baseline data**	15	A table showing baseline demographic and clinical	Baseline characteristics for the individual and cluster levels as	27

For peer review only

		characteristics for each group	applicable for each group	
**Numbers analysed**	16	For each group, number of participants (denominator) included in each analysis and whether the analysis was by original assigned groups	For each group, number of clusters included in each analysis	27
**Outcomes and estimation**	17a	For each primary and secondary outcome, results for each group, and the estimated effect size and its precision (such as 95% confidence interval)	Results at the individual or cluster level as applicable and a coefficient of intracluster correlation (ICC or k) for each primary outcome	28
	17b	For binary outcomes, presentation of both absolute and relative effect sizes is recommended		**28-29**
**Ancillary analyses**	18	Results of any other analyses performed, including subgroup analyses and adjusted analyses, distinguishing pre-specified from exploratory		**28-29**
**Harms**	19	All important harms or unintended effects in each group (for specific guidance see CONSORT for harms[3])		**N/A**
**Discussion**				
**Limitations**	20	Trial limitations, addressing sources of potential bias, imprecision, and, if relevant, multiplicity of analyses		**14-18**
**Generalisability**	21	Generalisability (external validity, applicability) of the trial findings	Generalisability to clusters and/or individual participants (as relevant)	14-18
**Interpretation**	22	Interpretation consistent with results, balancing benefits and harms, and considering other relevant evidence		**14-18**
**Other information**				
**Registration**	23	Registration number and		**3 and 22**

		name of trial registry	
**Protocol**	24	Where the full trial protocol can be accessed, if available	**20, Ref 15**
**Funding**	25	Sources of funding and other support (such as supply of drugs), role of funders	**3 and 23**

** Note: page numbers optional depending on journal requirements*

For peer review only

Table 2. Items to include when reporting a randomized trial in a journal or conference abstract

Item	Description	Reported on line number
Title	Identification of the study as randomized	Y
Authors *	Contact details for the corresponding author	Y
Trial design	Description of the trial design (e.g. parallel, cluster, non-inferiority)	Y
Methods		
Participants	Eligibility criteria for participants and the settings where the data were collected	Y
Interventions	Interventions intended for each group	Y
Objective	Specific objective or hypothesis	Y
Outcome	Clearly defined primary outcome for this report	Y
Randomization	How participants were allocated to interventions	Y
Blinding (masking)	Whether or not participants, care givers, and those assessing the outcomes were blinded to group assignment	N
Results		
Numbers randomized	Number of participants randomized to each group	N/A (Number of clusters provided)
Recruitment	Trial status	N
Numbers analysed	Number of participants analysed in each group	N/A (Number of clusters provided)
Outcome	For the primary outcome, a result for each group and the estimated effect size and its precision	Y
Harms	Important adverse events or side effects	N/A
Conclusions	General interpretation of the results	Y
Trial registration	Registration number and name of trial register	Y
Funding	Source of funding	Y

**this item is specific to conference abstracts*
